# Biphasic unbinding of a metalloregulator from DNA for transcription (de)repression in Live Bacteria

**DOI:** 10.1093/nar/gkaa056

**Published:** 2020-02-03

**Authors:** Won Jung, Kushal Sengupta, Brian M Wendel, John D Helmann, Peng Chen

**Affiliations:** 1 Department of Chemistry and Chemical Biology, Cornell University, Ithaca, NY 14853, USA; 2 Department of Microbiology, Cornell University, Ithaca, NY 14853, USA

## Abstract

Microorganisms use zinc-sensing regulators to alter gene expression in response to changes in the availability of zinc, an essential micronutrient. Under zinc-replete conditions, the Fur-family metalloregulator Zur binds to DNA tightly in its metallated repressor form to Zur box operator sites, repressing the transcription of zinc uptake transporters. Derepression comes from unbinding of the regulator, which, under zinc-starvation conditions, exists in its metal-deficient non-repressor forms having no significant affinity with Zur box. While the mechanism of transcription repression by Zur is well-studied, little is known on how derepression by Zur could be facilitated. Using single-molecule/single-cell measurements, we find that in live *Escherichia coli* cells, Zur's unbinding rate from DNA is sensitive to Zur protein concentration in a first-of-its-kind biphasic manner, initially impeded and then facilitated with increasing Zur concentration. These results challenge conventional models of protein unbinding being unimolecular processes and independent of protein concentration. The facilitated unbinding component likely occurs via a ternary complex formation mechanism. The impeded unbinding component likely results from Zur oligomerization on chromosome involving inter-protein salt-bridges. Unexpectedly, a non-repressor form of Zur is found to bind chromosome tightly, likely at non-consensus sequence sites. These unusual behaviors could provide functional advantages in Zur's facile switching between repression and derepression.

## INTRODUCTION

Zinc is an essential transition metal micronutrient for cells because it functions as enzyme co-factors, and structural or regulatory factors, but it can also become harmful when in excess (e.g. interfere with other ligand-protein interactions for enzymatic activities or with transporters for acquiring other essential metals) (1–4). Organisms have thus developed uptake, storage, export and regulation mechanisms to maintain the proper levels of zinc inside the cell (5–8). One of the primary mechanisms for this zinc homeostasis is transcriptional regulation via metalloregulators. For example, in *Escherichia coli*, Zur is an ultrasensitive Fur-family metalloregulator that senses cytoplasmic Zn^2+^ concentrations. When buffered Zn^2+^ levels exceed ∼0.2 fM (1 fM = 10^−15^ M) of nominally free zinc, zinc uptake systems are repressed. Conversely, when free Zn^2+^ levels exceed ∼1.2 fM, ZntR, a member of MerR-family metalloregulators, activates zinc export systems (9).

In *E. coli*, Zur, like other Fur-family members, is a homo-dimer and requires two zinc atoms per monomer to function as an active repressor: one at site A with Cys103, Cys106, Cys143 and Cys146 as ligands, where the bound zinc cannot be removed even through overnight incubation with 500 μM TPEN (a chelator that binds Zn^2+^ with 0.3 fM affinity (10,11)), and the other at site B with His77, Cys88, His96 and Glu111 as ligands (12,13). Zinc depletion causes Zur to transition from a high DNA-affinity closed conformation to an open conformation, which acts as a non-repressor, leading to derepression of four identified Zur's regulons including zinc uptake gene cluster *znuABC*, the periplasmic zinc trafficking protein *zinT*, a pair of ribosomal proteins *L31p and L36p*, and a periplasmic lysozyme inhibitor *pliG* (12,14–16). O’Halloran and coworkers have shown that *in vitro* the C103S mutation, which perturbs site A, leads to disruption of Zur's dimeric structure and loss of its repressor function, giving site A a more structural role (12,13). On the other hand, the C88S mutant, in which site B is perturbed, stays dimeric but does not show any observable affinity to cognate DNA up to 300 nM of protein concentration even in the presence of 50 μM Zn^2+^, which is 10^9^ times higher than the intracellular free Zn^2+^ concentration (∼femtomolar (9)); consistently, this mutant behaves as a non-repressor, giving site B a more sensing role (12,13). Studies on Zur in *B. subtilis* also showed the two types of zinc binding sites (17). Moreover, under excess zinc, the C88S mutant of *E. coli* Zur can bind cognate DNA but with an affinity of ∼100 nM, ∼30 times weaker than the wild-type Zur. The crystal structure of metallated repressor form of *E. coli* Zur in complex with a 33-bp cognate DNA derived from the *znuABC* promoter further identified that two Zur dimers can bind to DNA simultaneously with two Asp49−Arg52 salt-bridge interactions between the two dimers, and the binding of two dimers are highly cooperative as shown by gel-shift assays (12).

The current understanding of Zur's mode of action at its operator site is described by an on-off model in which its repressor form binds to its cognate operator sites tightly, and its non-repressor forms have insignificant affinity to operator sites (12,13,17–20). This is in contrast to ZntR (and its Cu^1+^ sensing homologue CueR), which operates via a DNA distortion mechanism in transcriptional regulation (21,22): its zinc-bound activator form and zinc-depleted repressor form both bind promoter operator sites tightly but distort the DNA structure differently to result in different RNA polymerase interactions that prefer either an open complex for activating transcription or a dead-end closed-like complex for repressing transcription (21,23).

Although the mechanism of transcription repression by Zur is well-studied, much less is known about how repression is reversed. Facile derepression is important, however, especially when cells encounter Zn-deficient growth environment. A simple scenario would be zinc dissociation to convert a metallated-Zur to its non-repressor form, which would then unbind from an operator site promptly, leading to derepression; yet it is unlikely as Zur binds Zn^2+^ with tight femtomolar affinity (9). Moreover, since binding of Zn^2+^ increased Zur's DNA-binding affinity, the converse must also be true and the Zur:Zn:DNA complex binds Zn^2+^ even tighter than Zur in solution. Another scenario would be the spontaneous unbinding of the metallated Zur from DNA, which is not expected to be very facile, either, as the metallated Zur binds to operator sites also tightly with nanomolar affinity (9,12).

The unbinding of regulatory proteins from their operator sites is usually a unimolecular reaction (i.e. spontaneous unbinding), whose first-order rate constant is independent of surrounding regulator concentration. However, recent *in vitro* and *in vivo* single-molecule studies of CueR and ZntR showed facilitated unbinding in which the first-order unbinding rate constant increases with increasing surrounding protein concentrations (24,25). Similar behaviors were observed for nucleoid associated proteins that bind double-stranded DNA nonspecifically (26), replication protein A that binds single-stranded DNA nonspecifically (27), and DNA polymerases (28,29). A mechanistic consensus arose and it involves multivalent contacts between the protein and DNA (30), which enables the formation of ternary complexes as intermediates that subsequently give rise to concentration-enhanced protein unbinding kinetics. Whether this facilitated unbinding mechanism applies to Zur (and Fur-family metalloregulators) is unknown, and Zur−DNA interaction kinetics remain to be characterized.

Here, we use single-molecule tacking (SMT; list of abbreviations is in Supplementary Table S8) coupled with single-cell quantification of protein concentration (SCQPC) to study Zur−DNA interactions in live *E. coli* cells. We found that the unbinding kinetics of Zur from the chromosome, in both its repressor and non-repressor forms, not only show facilitated unbinding with increasing cellular protein concentrations, can also exhibit a biphasic behavior, with an initial impeded unbinding followed by facilitated unbinding as protein concentrations increase. The impeded unbinding likely stems from Zur oligomerization on DNA, where inter-dimer salt bridges play a key role. In addition, the non-repressor form, previously thought to not bind DNA significantly, can bind to chromosome tightly, likely at non-consensus sites. Taken together, these mechanisms likely facilitate transcription switching between repression and derepression by Zur in cells depending on fluctuations in the environmental zinc concentrations.

## MATERIALS AND METHODS

### Bacterial strains and sample preparation

All strains were derived from the *E. coli* BW25113 strain as detailed in Supplementary Data Section 1. Zur^mE^ was either encoded at its chromosomal locus via lambda-red homologous recombination (31) or in a pBAD24 plasmid in a Δ*zur* deletion strain (32). Mutant forms of Zur (${\rm{Zur}}_{{\rm{C88S}}}^{{\rm{mE}}}$, ${\rm{Zur}}_{{\rm{D49A}}}^{{\rm{mE}}}$ or ${\rm{Zur}}_{{\rm{C88S,\ D49A}}}^{{\rm{mE}}}$; strain names are summarized in Supplementary Table S9) were generated via site-directed mutagenesis in pBAD24, which was introduced into the Δ*zur* strain. More details are in Supplementary Data Section 1 (plasmids, primers, and strains used are summarized in Supplementary Tables S1–S3).

All cell imaging experiments were done at room temperature in M9 medium supplemented with amino acids, vitamins, and 0.4% glycerol. 20 μM ZnSO_4_ was used for Zn replete conditions. The cells were immobilized on an agarose pad in a sample chamber (Supplementary Figure S2A). Details are in Supplementary Data Section 3.

### Biochemical analyses

Western blot was performed to confirm the intactness of the Zur^mE^ fusion protein (Supplementary Data Section 2.1). A strain that expresses Zur^mE^ from a pBAD24 plasmid was used to help with Western blot detection. Reverse transcription PCR analysis of *zur* regulon transcripts (e.g. *zinT* and *znuC*) was used to confirm the repressor or non-repressor function of Zur^mE^ or ${\rm{Zur}}_{{\rm{C88S}}}^{{\rm{mE}}}$ in cells, as well as to probe the dominant cellular forms of Zur under various zinc concentrations in the growth media (Supplementary Data Sections 2.2 - 2.4). The total Zn concentrations in the media were quantified by ICP-MS or a zinc quantification kit (Supplementary Data Section 2.3 and Supplementary Table S4).

### SMT and SCQPC

SMT and SCQPC were performed on an inverted fluorescence microscope, as reported (24) (Supplementary Figure S2B). For SMT, inclined epi-illuminated 405 and 561 nm lasers photoconverted and excited single mEos3.2 molecules, respectively. 561 nm excitation-imaging were in stroboscopic mode, with 4 ms laser excitation pulses separated by 40 ms time lapse, synchronized with the camera exposure, so that the mobile proteins still appear as diffraction-limited spots. A custom-written MATLAB software was used to identify diffraction-limited fluorescence spots and fit them with two-dimensional Gaussian functions, giving ∼20 nm localization precision (24,33). Time trajectories of positions and displacement length *r* between adjacent images were then extracted.

SCQPC was performed after SMT. The remaining proteins were firstly photoconverted to the red form by a long 405 nm laser illumination. The total cell red fluorescence was then imaged by the 561 nm laser to determine the protein copy number, provided the average fluorescence of a single mEos3.2 from the earlier SMT. This step was performed twice to ensure complete photoconversion of all the remaining proteins. The photoconversion efficiency of mEos3.2 (34) and dimeric state of Zur were accounted for. Cell volumes were determined by fitting their optical transmission image contours with the model geometry of a cylinder with two hemispherical caps (distributions of extracted cell geometric parameters are in Supplementary Figure S3).

### Data analyses

The data analysis procedures for resolving the diffusion states from single-molecule displacement length distribution, determining the unbinding rate constant from residence time distributions, quantifying the relative populations of different states, and cluster analysis are described in detail in the Supplementary Data Sections 4–9. Bootstrapping shows the statistical reliability of data (detailed in Supplementary Figure S18).

## RESULTS

### SMT and SCQPC uncover facilitated unbinding of repressor form of Zur from DNA in cells

To visualize individual Zur proteins in *E. coli* cells, we fused the photoconvertible fluorescent protein mEos3.2 (34–36) to Zur's C-terminus creating Zur^mE^, either at its chromosomal locus to have physiological expression or in an inducible plasmid in a Δ*zur* deletion strain to access a wider range of cellular protein concentrations (Supplementary Figure S4 and Supplementary Data Section 1). This Zur^mE^ fusion-protein is intact and is as functional a repressor as the WT under Zn replete growth conditions (Supplementary Figure S1).

**Figure 1.**
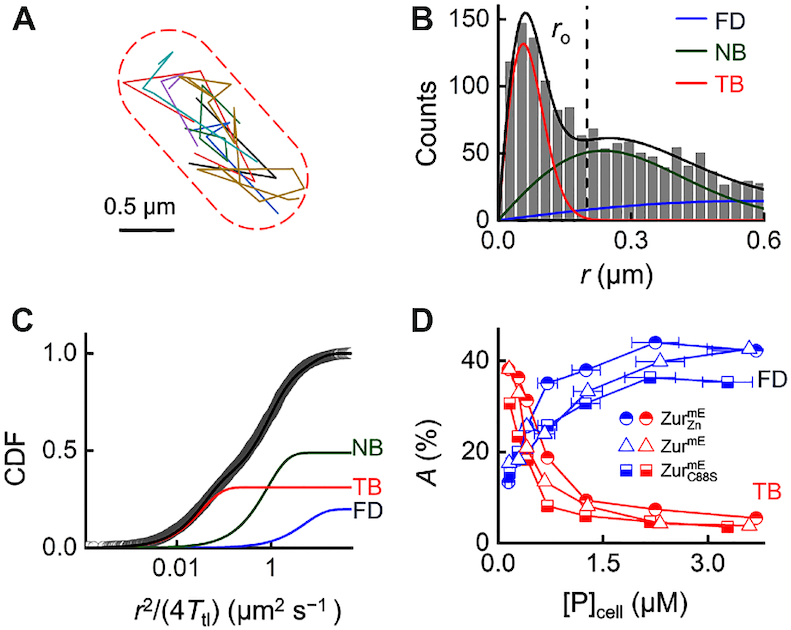
SMT of Zur in living cells. (**A**) Overlay of many position trajectories of single ${\rm{Zur}}_{{\rm{Zn}}}^{{\rm{mE}}}$ proteins in a live *E. coli* cell. Dash lines: cell boundary. (**B**) Histogram of displacement length *r* per time-lapse (40 ms) of >1,700 tracked ${\rm{Zur}}_{{\rm{Zn}}}^{{\rm{mE}}}$ proteins at ${[ {{\rm{Zur}}_{{\rm{Zn}}}^{{\rm{mE}}}} ]_{{\rm{cell}}}}$ = 290 ± 33 nM. Solid lines: the overall fitted distribution (black), and the resolved FD (blue), NB (green), and TB (red) diffusion states (Supplementary Data Section 4). Vertical dashed line: *r*_o_ = 0.2 μm for extracting residence times as in Figure 2A. (**C**) Cumulative-distribution-function (CDF) of *r* (plotted against $\frac{{{{{r}}^{\rm{2}}}}}{{{\rm{4}}{{{T}}_{{\rm{tl}}}}}}$) from (B). Lines: overall fit (Supplementary Equation S10) and three resolved diffusion states with effective diffusion constants (and fractional populations): *D*_FD_ = 6.7 ± 0.5 μm^2^ s^−1^ (18.1 ± 0.3%), *D*_NB_ = 0.82 ± 0.05 μm^2^ s^−1^ (47.2 ± 0.6%), and *D*_TB_ = 0.033 ± 0.003 μm^2^ s^−1^ (34.7 ± 0.6%). (**D**) Fractional populations of FD and TB states for ${\rm{Zur}}_{{\rm{Zn}}}^{{\rm{mE}}}$, Zur^mE^, and ${\rm{Zur}}_{{\rm{C88S}}}^{{\rm{mE}}}$ versus the cellular protein concentrations (note: NB states here are omitted to avoid crowdedness and are summarized in Supplementary Table S6).

Using sparse photoconversion and time-lapse stroboscopic imaging, we tracked the motions of photoconverted Zur^mE^ proteins individually in single *E. coli* cells at tens of nanometer precision until their mEos3.2 tags photobleached (Figure 1A). This SMT allows for measuring Zur^mE^’s mobility, which reports on whether the molecule is freely diffusing in the cell or bound to DNA. We repeated this photoconversion and SMT cycle 500 times (30 imaging frames per cycle) for each cell, during which we counted the number of tracked protein molecules. We then used the SCQPC protocol to quantify the remaining number of Zur^mE^ protein molecules in the same cell (24), eventually determining the total Zur^mE^ concentration in each cell (i.e. ${\rm{[Zu}}{{\rm{r}}^{{\rm{mE}}}}{\rm{]_{cell}}}$ in units of dimers). This single-cell protein quantitation allowed for sorting the cells into groups of similar protein concentrations and subsequently examining protein-concentration−dependent processes, without being limited by the cell-to-cell heterogeneity in protein expression.

We first examined Zur^mE^ under Zn^2+^ replete conditions (20 μM Zn^2+^ added in the M9 medium). Quantitation of mRNA of *zur* regulons (e.g. *znuC* and *zinT*) in cells with various zinc concentrations in the medium indicated that 20 μM Zn^2+^ can evoke maximal repression of *zur* regulons (Supplementary Data 2.3). Therefore, the Zur proteins in the cell should be dominated by the fully metallated repressor form; we denote this condition as ${\rm{Zur}}_{{\rm{Zn}}}^{{\rm{mE}}}$. To quantify ${\rm{Zur}}_{{\rm{Zn}}}^{{\rm{mE}}}$ mobility in cells, we determined the distribution of its displacement length *r* between successive images and the corresponding cumulative distribution function (CDF) of *r* for each cell group having similar ${[ {{\rm{Zur}}_{{\rm{Zn}}}^{{\rm{mE}}}} ]_{{\rm{cell}}}}$ (Figure 1B and C) (37–41). Global analysis of these CDFs across all cellular protein concentrations resolved minimally three Brownian diffusion states with effective diffusion constants of ∼6.6 ± 0.5, 0.82 ± 0.05 and 0.033 ± 0.003 μm^2^ s^−1^ (Figure 1B and C; Supplementary Table S5). From the spatial distribution of tracked ${\rm{Zur}}_{{\rm{Zn}}}^{{\rm{mE}}}$ proteins in the cell, we also did not discern any subcellular localization or protein aggregation, which would give immobile protein clusters; therefore, these two aspects are not the reasons for the presence of these three diffusion states. On the basis of their diffusion constants and previous studies of transcription regulator diffusion in *E. coli* (24,42–45), we assigned the fastest diffusion state as ${\rm{Zur}}_{{\rm{Zn}}}^{{\rm{mE}}}$ proteins freely diffusing (FD) in the cytoplasm, the medium diffusion state as those nonspecifically bound (NB) to and moving on chromosome, and the slowest state as those tightly bound (TB; note that TB does not imply the sequence specificity of the binding) to the chromosome, whose small effective diffusion constant reflects chromosome dynamics (43,46) and position localization uncertainties. The TB state of ${\rm{Zur}}_{{\rm{Zn}}}^{{\rm{mE}}}$ is also expected here because metallated Zur is known to bind specifically to consensus operator sites (Zur boxes) within Zur-regulated promoters. Control measurements on the free mEos3.2 further support the assignment of the FD state, as we reported (24).

The resolution of CDFs of *r* also gave the fractional populations of the three states amid all ${\rm{Zur}}_{{\rm{Zn}}}^{{\rm{mE}}}$ protein molecules as a function of its cellular concentrations (Figure 1D; Supplementary Table S6). With increasing ${\rm{\ }}{[ {{\rm{Zur}}_{{\rm{Zn}}}^{{\rm{mE}}}} ]_{{\rm{cell}}}}$, the fractional population of the FD state increases, while that of the TB state decreases. These trends further support their assignments because, with increasing cellular protein concentrations, more proteins compete for the limited number of tight binding sites on the chromosome, leading to smaller fractional populations of the TB state and larger fractions of the FD state of ${\rm{Zur}}_{{\rm{Zn}}}^{{\rm{mE}}}$ proteins.

**Figure 2.**
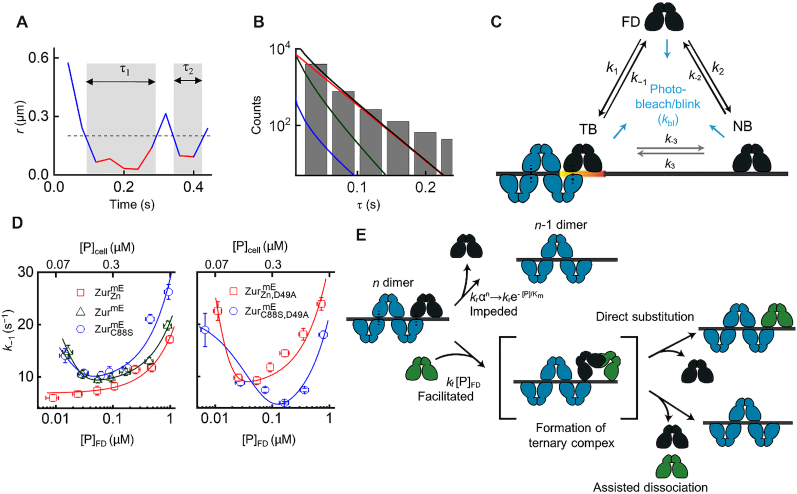
Biphasic unbinding kinetics of Zur from TB sites on chromosome. (**A**) Time trajectory of displacement length *r* per time-lapse from a single ${\rm{Zur}}_{{\rm{Zn}}}^{{\rm{mE}}}$ protein. Two microscopic residence time *τ* shown in gray shades; dashed horizontal line: displacement threshold *r*_o_ = 0.2 μm (i.e. vertical dashed line in Figure 1B). (**B**) Histogram of *τ* for ${\rm{Zur}}_{{\rm{Zn}}}^{{\rm{mE}}}$ at ${[ {{\rm{Zur}}_{{\rm{Zn}}}^{{\rm{mE}}}} ]_{{\rm{cell}}}}$ = 290 ± 33 nM. Black line: fitting with Supplementary Equation S15. Contributions of the three diffusion states are plotted, as color-coded in Figure 1B, C. (**C**) Three-state model of a single Zur protein interacting with DNA in a cell. *k*’s are the rate constants. (**D**) Protein-concentration-dependent *k*_−1_ for ${\rm{Zur}}_{{\rm{Zn}}}^{{\rm{mE}}}$, Zur^mE^, and ${\rm{Zur}}_{{\rm{C88S}}}^{{\rm{mE}}}$ (left) and the D49A salt-bridge mutants (right). Bottom/top axis refers to free/total cellular protein concentration, respectively. Lines are fits with Equation (2). All error bars are s.d. (derived from the goodness of the fit). (**E**) Schematic molecular mechanism for biphasic unbinding of Zur from a TB site. A bound Zur protein (dark blue) within an oligomer on DNA can unbind following either an impeded pathway (top) due to the presence of the other (*n* – 1) proteins in the oligomer or a facilitated pathway (bottom) upon binding another protein (green) to form an intermediate ternary complex, which then proceeds through direct substitution or assisted dissociation pathway. Black dashed lines denote salt-bridge interactions.

To probe the interaction dynamics between ${\rm{Zur}}_{{\rm{Zn}}}^{{\rm{mE}}}$ and DNA in the cell, we examined the *r* versus time *t* trajectories of individual ${\rm{Zur}}_{{\rm{Zn}}}^{{\rm{mE}}}$ proteins. These trajectories show clear transitions between large and small *r* values (Figure 2A): the small *r* values are expected to be dominated by instances of ${\rm{Zur}}_{{\rm{Zn}}}^{{\rm{mE}}}$ tightly bound to chromosome (i.e. TB state). We set an upper threshold *r*_0_ (=0.2 μm), below which >99.5% of the TB states are included based on the resolved distributions of *r* (Figure 1B), to select these small displacements and obtain estimates of the individual residence time *τ* of a single Zur protein at a chromosomal tight binding site (Figure 2A). Each *τ* starts when *r* drops below *r*_0_ and ends when *r* jumps above *r*_0_ (e.g. *τ*’s in Figure 2A), which are expected to reflect dominantly protein unbinding from DNA, or when the mEos3.2-tag photobleaches/blinks.

We analyzed trajectories from many cells of similar ${\rm{\ }}{[ {{\rm{Zur}}_{{\rm{Zn}}}^{{\rm{mE}}}} ]_{{\rm{cell}}}}$ to obtain their corresponding distribution of *τ* (Figure 2B). We used a quantitative three-state model (i.e. FD, NB and TB states; Figure 2C and Supplementary Figure S13) to analyze the distribution of *τ*, in which the contributions of FD and NB states are deconvoluted (Supplementary Equation S15; approximations and validations of this model in Supplementary Data Section 5) (24). This model also accounts for mE photobleaching/blinking kinetics, determined from the fluorescence on-time distribution of SMT trajectories (Supplementary Figure S8). This analysis gave *k*_−1_, the apparent first-order unbinding rate constant of ${\rm{Zur}}_{{\rm{Zn}}}^{{\rm{mE}}}$ from a tight binding site on the chromosome, for each group of cells having similar ${[ {{\rm{Zur}}_{{\rm{Zn}}}^{{\rm{mE}}}} ]_{{\rm{cell}}}}$.

Strikingly, *k*_−1_ for ${\rm{Zur}}_{{\rm{Zn}}}^{{\rm{mE}}}$ shows a facilitated unbinding behavior within the accessible cellular protein concentration range (∼80 nM to ∼2 μM)—it increases linearly with increasing free (or total) cellular protein concentrations (Figure 2D, left, red points). This behavior is also apparent in the simple averages of residence time 〈*τ*〉 or by analyzing the distributions of *τ* with an exponential decay function fit whose exponent merely considers mE photobleaching/blinking (Supplementary Figure S9A). This facilitated unbinding of ${\rm{Zur}}_{{\rm{Zn}}}^{{\rm{mE}}}$ is analogous to our previous findings on CueR and ZntR (two MerR-family metalloregulators) (24,25), thus extending the generality of this mechanism to a Fur-family metalloregulator.

### Concentration-dependent biphasic unbinding kinetics of non-repressor form of Zur from DNA

We next examined Zur^mE^ in cells grown in regular M9 medium (which contains ∼0.05 μM Zn; Supplementary Table S4); we refer to this condition as Zur^mE^. Under this condition, the cellular Zur proteins should have a significant fraction that has non-metallated site B and is in the non-repressor form. Indeed, mRNA quantitation shows that in this regular M9 medium, the *znuC* gene regulated by Zur is not as repressed as in the 20 μM Zn^2+^ replete conditions (Supplementary Data Section 2.4), supporting the presence of non-repressor forms of Zur in the cell. From SMT measurements, the same three diffusion states with effective diffusion constants of ∼4.9 ± 0.6, 0.92 ± 0.07 and 0.040 ± 0.004 μm^2^ s^−1^ are resolved in the CDFs of *r* across all cellular protein concentrations (Supplementary Table S5 and Supplementary Data Section 4); similar trends in their fractional populations-vs-cellular protein concentration are observed (Figure 1D; Supplementary Figures S6 and S7).

We again analyzed displacement-vs-time trajectories and the thresholded residence times to probe Zur-DNA interaction dynamics. Surprisingly, *k*_−1_ for Zur^mE^ shows a biphasic, impeded-followed-by-facilitated behavior: it initially decreases with increasing free (or total) cellular Zur concentration (i.e. impeded), reaching a minimum at ∼320 nM; it then increases toward higher protein concentrations (i.e. facilitated; Figure 2D, left, green points). This biphasic behavior is again apparent in the simple averages of residence time 〈*τ*〉 or by analyzing the distributions of *τ* that merely corrects for mE photobleaching/blinking (Supplementary Figure S9B). As the facilitated unbinding component was already observed above for ${\rm{Zur}}_{{\rm{Zn}}}^{{\rm{mE}}}$, which is dominated by the metallated repressor form of Zur, the new, impeded unbinding behavior of Zur^mE^ should come from contributions from the non-repressor form of Zur. The lack of such impeded unbinding behavior for ${\rm{Zur}}_{{\rm{Zn}}}^{{\rm{mE}}}$ (i.e. Zur^mE^ under Zn replete conditions) also supports that ${\rm{Zur}}_{{\rm{Zn}}}^{{\rm{mE}}}$ is dominated by the fully metallated repressor form of Zur, which binds to operator sites tightly.

To confirm whether the impeded unbinding behavior of Zur^mE^ indeed comes from the non-repressor form of Zur, we examined the zinc-binding site B mutant C88S (i.e., ${\rm{Zur}}_{{\rm{C88S}}}^{{\rm{mE}}}$) in cells grown in regular M9 medium. Gel shift assays have shown that this mutant remains as a dimer, acts as a non-repressor under physiologically relevant conditions, and does not show any observable affinity to cognate DNA (i.e. *K*_D_ > 300 nM at the *znuABC* promoter) (12). Our mRNA quantitation of Zur regulons in the cell further confirms ${\rm{Zur}}_{{\rm{C88S}}}^{{\rm{mE}}}$ to be largely in a non-repressor state even under Zn replete growth conditions (Supplementary Data Section 2.2).

Analysis of CDFs of *r* for ${\rm{Zur}}_{{\rm{C88S}}}^{{\rm{mE}}}$ still resolves the same three diffusion states (Supplementary Figure S5). The presence of a significant fraction of the tight DNA-binding state, even at low cellular protein concentrations, is surprising (e.g. ∼32% at [${\rm{Zur}}_{{\rm{C88S}}}^{{\rm{mE}}}$]_cell_ ∼ 150 nM compared with ∼38% for the metallated ${\rm{Zur}}_{{\rm{Zn}}}^{{\rm{mE}}}$; Figure 1D; 1 nM in an *E. coli* cell corresponds to ∼1 protein copy), as this C88S mutant is a non-repressor and does not bind to cognate operator sites in *E. coli* (12) (for *B. subtilis* Zur, its binding affinity to operator sites in the zinc limiting condition is ∼1000 times weaker than that in the presence of sufficient amount of Zn^2+^ (17)). We hypothesized that the TB state of ${\rm{Zur}}_{{\rm{C88S}}}^{{\rm{mE}}}$ must come from its binding to non-operator sites (i.e. non-consensus sequence sites or sites with a consensus distinct from the Zur box; see later). Strikingly, *k*_−1_ of ${\rm{Zur}}_{{\rm{C88S}}}^{{\rm{mE}}}$ shows a clear biphasic behavior with its increasing cellular concentration, like Zur^mE^, but it is overall larger than the *k*_−1_ of Zur^mE^, which in turn is larger than that of ${\rm{Zur}}_{{\rm{Zn}}}^{{\rm{mE}}}$ (Figure 2D, left), consistent with that ${\rm{Zur}}_{{\rm{C88S}}}^{{\rm{mE}}}$ behaves as a non-repressor while Zur^mE^ is a mixture and therefore is in-between non-repressor and repressor behaviors. Taken together, the non-repressor form of Zur indeed exhibits impeded unbinding initially with increasing cellular protein concentration, which is a first-of-its-kind discovery, and it also shows facilitated unbinding as well, leading to the overall biphasic unbinding behavior from DNA. And such biphasic unbinding behavior of the Zur non-repressor form is from tight-binding, non-consensus sequence sites in the chromosome distinct from Zur operator sites. (We could also rule out that this biphasic unbinding is not due to different sampling of various tight-binding sites on chromosome with increasing cellular protein concentrations; Supplementary Data Section 7.3.)

### Mechanism of biphasic unbinding of Zur from DNA

Amid the biphasic unbinding of Zur from DNA (Figure 2D, left), the concentration-facilitated unbinding at higher protein concentrations is analogous to those of CueR and ZntR (24). There it stems from an assisted dissociation pathway, in which an incoming protein from solution helps an incumbent protein on DNA to unbind, or a direct substitution pathway, in which the incoming protein directly replaces the incumbent one (Figure 2E, lower; Supplementary Figure S10) (24,25). The rates of both pathways depend linearly on the free protein concentration, and both likely occur through a common ternary protein_2_−DNA complex, in which the two homodimeric proteins each use one DNA-binding domain to bind to half of the dyad recognition sequence (30,47). As Zur is also a homodimer, Zur therefore could form this ternary complex and undergo assisted dissociation or direct substitution, leading to its concentration-facilitated unbinding from DNA.

Regarding the impeded unbinding of Zur's non-repressor form in the lower concentration regime, we propose that it likely results from protein oligomerization around the DNA binding site, in which the number of proteins in the oligomer increases with increasing protein concentration and the resulting protein-protein interactions contribute to additional stabilization, thereby decreasing protein unbinding rate (Figure 2E, upper; Supplementary Figure S11). (The facilitated unbinding later takes over when the protein concentration reaches a high enough level.) Two evidences support our oligomerization proposal: (1) Crystallography showed that two *E. coli* Zur dimers can bind to a short cognate DNA sequence (12). (2) DNA footprinting showed that *S. coelicoror* Zur forms oligomers around its recognition sites, containing greater than four dimers (48).

**Figure 3.**
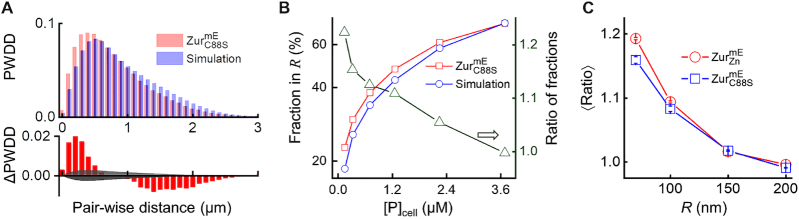
Spatial properties of Zur's residence sites. (**A**) Normalized pair-wise distance distributions (PWDDs) of residence sites for ${\rm{Zur}}_{{\rm{C88S}}}^{{\rm{mE}}}$ and for simulated random sites in the cell (top), and the difference of ${\rm{Zur}}_{{\rm{C88S}}}^{{\rm{mE}}}$ from simulation (bottom; gray area indicates the 95% confidence bounds). (**B**) Fraction of residence sites within a radius threshold *R* (=100 nm, left axis) for ${\rm{Zur}}_{{\rm{C88S}}}^{{\rm{mE}}}$ and for simulated random sites as a function of cellular protein concentration. Their ratio (${\rm{Zur}}_{{\rm{C88S}}}^{{\rm{mE}}}$ vs. simulation) is plotted against the right axis. (**C**) Dependence of the average ratio in *B* across all protein concentrations as a function of the radius threshold *R* for ${\rm{Zur}}_{{\rm{C88S}}}^{{\rm{mE}}}$ and ${\rm{Zur}}_{{\rm{Zn}}}^{{\rm{mE}}}$. Error bars are s.d.

To further support this oligomerization proposal, we examined the spatial distribution in the cell of Zur's residence sites at its TB state; these residence sites correspond to the *r*_0_-thresholded small displacements (Figure 2A; Supplementary Data Section 8). For comparison, we further simulated an equal number of sites randomly distributed in a cell of the same size (Supplementary Data Section 8.1 and Supplementary Figure S16). We then examined their pair-wise distance distributions (PWDD), in which Zur oligomerization at chromosomal binding sites should lead to more populations at shorter pair-wise distances. This PWDD for the non-repressor ${\rm{Zur}}_{{\rm{C88S}}}^{{\rm{mE}}}$ indeed shows a higher population at distances shorter than ∼500 nm relative to the simulated random sites (Figure 3A; Supplementary Figure S17A for${\rm{\ Zur}}_{{\rm{Zn}}}^{{\rm{mE}}}$). However, at the distance scale of a few hundred nanometers, the compaction of chromosome also contributes to the PWDD of residence sites (24). To decouple the contribution of protein oligomerization from chromosome compaction, we examined the fraction of residence sites within a radius threshold *R*. At small *R* (e.g. <100 nm), the contribution of Zur oligomerization to this fraction should dominate over chromosome compaction, as oligomerization is at molecular scale whereas the most compact chromosome in a *E. coli* cell is still around hundreds of nanometer in dimension (24,49). At any specified *R* (e.g. 200 nm), the fraction of ${\rm{Zur}}_{{\rm{C88S}}}^{{\rm{mE}}}$ residence sites within the radius *R* increases expectedly with increasing cellular protein concentrations (Figure 3B, red points), because higher protein concentrations gave higher sampling frequency of residence sites. More important, at smaller *R* (e.g. 100 nm), the fraction of ${\rm{Zur}}_{{\rm{C88S}}}^{{\rm{mE}}}$ residence sites is larger than that of simulated random sites (Figure 3B, red vs. blue points; Supplementary Figure S17B for${\rm{\ Zur}}_{{\rm{Zn}}}^{{\rm{mE}}}$), and their ratio is larger at lower protein concentrations (Figure 3B, green points, right *y*-axis). The average ratio of the fraction of ${\rm{Zur}}_{{\rm{C88S}}}^{{\rm{mE}}}$ residence sites over that of the simulated random sites is always greater than 1, and it becomes larger at smaller *R* down to <70 nm (Figure 3C; Supplementary Figure S17C for${\rm{\ Zur}}_{{\rm{Zn}}}^{{\rm{mE}}}$; note our molecular localization precision is ∼20 nm; Supplementary Data Section 3), supporting the oligomerization of the non-repressor ${\rm{Zur}}_{{\rm{C88S}}}^{{\rm{mE}}}$ at chromosomal tight binding sites at the nanometer scale.

We formulated a quantitative kinetic model to describe the biphasic unbinding of Zur's non-repressor form. It considers both oligomerization at a TB site and facilitated unbinding via a ternary protein_2_-DNA complex (Figure 2C and E; Supplementary Data Section 6). The microscopic unbinding rate constant ${{k}}_{{{ - 1}}}^{{{(n)}}}$ from a TB site with *n* non-repressor dimers bound as an oligomer comprises three terms (Supplementary Equation S21):

(1) $$\begin{equation*}{{k}}_{ - {{1}}}^{{{(n)}}}{\rm{\ = \ }}{{{k}}_{\rm{o}}}{\rm{\ + \ }}{{{k}}_{\rm{r}}}{{{\alpha }}^{{n}}}{\rm{\ + \ }}{{{k}}_{\rm{f}}}{{\rm{[P]}}_{{\rm{FD}}}}\end{equation*}$$

*k*_o_ is a first-order intrinsic unbinding rate constant. The *k*_r_*α^n^* term accounts for the impeded unbinding from protein oligomerization, where a first-order rate constant *k*_r_ is attenuated by the factor *α* (0 < *α* < 1) to the exponent of *n*, which depends on the cellular protein concentration and has a maximal value of *n*_0_, the oligomerization number. The third term describes the facilitated unbinding, with *k*_f_ being a second-order rate constant and [P]_FD_ being the concentration of freely diffusing Zur dimers in the cell, as reported for CueR/ZntR (24). In the limit of weak oligomerization and low free protein concentrations (approximations are verified experimentally; see Supplementary Data Section 6 and Supplementary Figure S12), the apparent unbinding rate constant *k*_−1_ from any TB site is (Supplementary Equation S25):

(2) $$\begin{equation*}{k_{ - 1}} = \left\langle {k_{ - 1}^{(n)}} \right\rangle = k_{\rm{o}}^{{\rm{off}}} + {k_{\rm{r}}}\left( {{e^{{\raise0.7ex\hbox{${ - {{\left[ {\rm{P}} \right]}_{{\rm{FD}}}}}$} \!\mathord{\left/ {\vphantom {{ - {{\left[ {\rm{P}} \right]}_{{\rm{FD}}}}} {{K_{\rm{m}}}}}}\right.} \!\lower0.7ex\hbox{${{K_{\rm{m}}}}$}}}} - 1} \right) + {k_{\rm{f}}}{\left[ {\rm{P}} \right]_{{\rm{FD}}}}\end{equation*}$$

**Figure 4.**
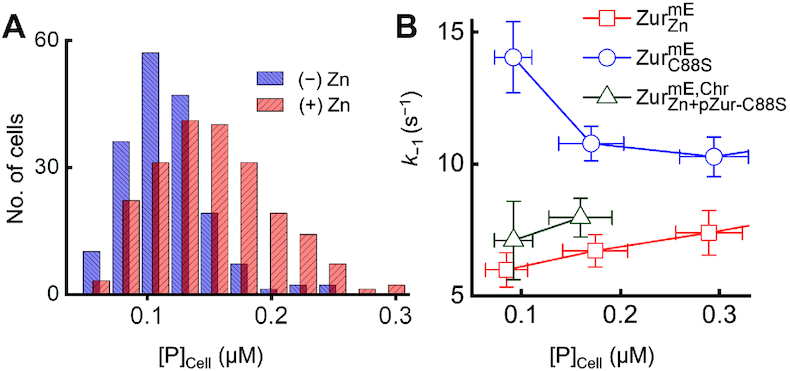
Zur behaviors within the physiological range of cellular protein concentrations. (**A**) Distribution of the chromosomally expressed Zur^mE^ concentration in the cell with (+) and without (−) 20 μM Zn^2+^ added in the medium. (**B**) Dependence of *k_–_*_1_ on the protein concentration in the cell for ${\rm{Zur}}_{{\rm{C88S}}}^{{\rm{mE}}}$, ${\rm{\ Zur}}_{{\rm{Zn}}}^{{\rm{mE}}}$ and for ${\rm{Zur}}_{{\rm{Zn}}}^{{\rm{mE}}}$ together with a plasmid expressing Zur_C88S_ (i.e. ${\rm{Zur}}_{{\rm{Zn + pZur - C88S}}}^{{\rm{mE,Chr}}}$) when the mE-tagged Zur is only encoded on the chromosome. The blue circles and red squares for ${\rm{Zur}}_{{\rm{C88S}}}^{{\rm{mE}}}$ and ${\rm{Zur}}_{{\rm{Zn}}}^{{\rm{mE}}}$ are part of data in Figure 2D (left). Error bars are s.d.

${K_m} = \frac{{k_{\rm{o}}^{{\rm{off}}}}}{{{k_1}( {1 - \alpha } )}}$; it has the units of protein concentration, reflecting the effective dissociation constant of the protein oligomer on the chromosome. ${{k}}_{\rm{o}}^{{\rm{off}}}$ = *k*_o_ + *k*_r_; it is a first-order spontaneous unbinding rate constant at the limit of zero concentration of freely-diffusing proteins. Equation 2 satisfactorily fits the biphasic unbinding kinetics of ${\rm{Zur}}_{{\rm{C88S}}}^{{\rm{mE}}}$ (Figure 2D, left), giving the associated kinetic parameters (Table 1 and Supplementary Table S7). In particular, *K*_m_ of ${\rm{Zur}}_{{\rm{C88S}}}^{{\rm{mE}}}$ is ∼12 nM, indicating that Zur's non-repressor form can oligomerize on chromosome at its physiological concentrations in the cells (∼120 nM; Figure 4A later).

**Table 1.** Kinetic and thermodynamic parameters for Zur–DNA interaction in *E. coli* cells (error bars are s.d.)

	Zur^mE^	${\rm{Zur}}_{{\rm{C88S}}}^{{\rm{mE}}}$	${\rm{Zur}}_{{\rm{Zn}}}^{{\rm{mE}}}$	${\rm{Zur}}_{{\rm{C88S,\ D49A}}}^{{\rm{mE}}}$	${\rm{Zur}}_{{\rm{Zn,\ D49A}}}^{{\rm{mE}}}$
*k*_1_(nM^−1^ s^−1^)^a^	0.80 ± 0.07	0.77 ± 0.08	0.46 ± 0.08	0.68 ± 0.24	0.55 ± 0.08
${{k}}_{\rm{o}}^{{\rm{off}}}$ (s^−1^)	25 ± 12	22 ± 21	5.4 ± 0.6	22 ± 2	36 ± 41
*k*_r_ (s^−1^)	16 ± 11	12 ± 20	n/o^b^	21 ± 1	27 ± 40
*k*_f_ (nM^−1^ s^−1^)	0.012 ± 0.002	0.018 ± 0.003	0.011 ± 0.014	0.021 ± 0.006	0.026 ± 0.004
*K*_m_ (nM)	14 ± 10	12 ± 17	n/o^b^	39 ± 18	7.6 ± 4.5
*K*_d1_ ( =${{k}}_{\rm{o}}^{{\rm{off}}}$/*k*_1_) (nM)^a^	31 ± 15	28 ± 27	12 ± 3	33 ± 12	67 ± 48
*K*_d2_ ( = *k*_-2_/*k*_2_) (nM)^a^	990 ± 80	830 ± 200	1300 ± 400	500 ± 160	1300 ± 300
*K*_d3_ ( = *k*_-3_/*k*_3_)^a^	0.011 ± 0.002	0.023 ± 0.007	0.022 ± 0.023	0.032 ± 0.062	0.008 ± 0.006
[D_0_]_NB_ (nM)^a^	2700 ± 200	2300 ± 500	2900 ± 700	2000 ± 500	3700 ± 800
[D_0_]_TB_⋅*n*_o_ (nM)^a^	100 ± 2	82 ± 8	130 ± 30	75 ± 12	92 ± 9

^a^ *n*_o_ = 5 was used in fitting; see Supplementary Figure S15 for *n*_o_ dependence of parameters.

[D_0_]_NB_: concentration of nonspecific binding sites in cell. [D_0_]_TB_: concentration of tight binding sites in cell.

^b^Not observed.

The same model also allowed for analyzing the relative populations of FD, NB and TB states of Zur across all cellular protein concentrations, giving additional thermodynamic and kinetic parameters (Table 1; Supplementary Table S7 and Figure S14). Strikingly, the dissociation constant *K*_d1_ of ${\rm{Zur}}_{{\rm{C88S}}}^{{\rm{mE}}}$ at TB sites of DNA is ∼28 nM, merely ∼2 times weaker than that of ${\rm{Zur}}_{{\rm{Zn}}}^{{\rm{mE}}}$ (*K*_d1_ ∼12 nM). This is *not* expected because the non-repressor form of Zur, in both *E. coli* and *B. subtilis*, was shown to have no significant affinity to Zur box recognized by its repressor form (12,17). Therefore, the high affinity of ${\rm{Zur}}_{{\rm{C88S}}}^{{\rm{mE}}}$ at the TB state suggests that inside cells, the non-repressor C88S mutant likely bind tightly to other, non-consensus sites in the chromosome, or consensus sites distinct from the Zur box. This former likelihood is supported by a ChIP-seq analysis in *B. subtilis*, which showed that Zur can bind tightly to many locations in the chromosome that do not share consensus with the known Zur box or with themselves (although it was undefined whether the detected bindings there were by metallated or non-metallated-Zur) (50). In addition, the extracted *k*_f_ for Zur^mE^ in the absence of added zinc is between those of ${\rm{Zur}}_{{\rm{Zn}}}^{{\rm{mE}}}$ and ${\rm{Zur}}_{{\rm{C88S}}}^{{\rm{mE}}}$ (differences here are larger than their errors), consistent with its behavior being a mixture of repressor and non-repressor forms of Zur.

### Molecular basis of impeded unbinding

Our model of Zur oligomerization at TB sites was based partly on the structure of two holo-Zur dimers bound to a cognate DNA, which showed two inter-dimer D49−R52 salt bridges (12). To probe the role of these salt bridges in Zur oligomerization, we made the D49A mutation, known to disrupt the interactions (12). For non-repressor ${\rm{Zur}}_{{\rm{C88S}}}^{{\rm{mE}}}$, the resulting mutant ${\rm{Zur}}_{{\rm{C88S,D49A}}}^{{\rm{mE}}}$ still exhibits the biphasic unbinding behavior, however the minimum of the apparent unbinding rate constant *k*_−1_ shifted to a higher cellular protein concentration (Figure 2D, right). Its *K*_m_ is 38.6 ± 17.9 nM, three times larger than that of ${\rm{Zur}}_{{\rm{C88S}}}^{{\rm{mE}}}$ (Table 1), indicating a weakened oligomerization affinity and thus a significant role of these salt bridges.

More strikingly, for ${\rm{Zur}}_{{\rm{Zn}}}^{{\rm{mE}}}$, which only showed facilitated unbinding (Figure 2D, left), the resulting mutant ${\rm{Zur}}_{{\rm{Zn,\ D49A}}}^{{\rm{mE}}}$ clearly shows biphasic unbinding with *K*_m_ = 7.6 ± 4.5 nM (Figure 2D, right; Table 1). Therefore, ${\rm{Zur}}_{{\rm{Zn}}}^{{\rm{mE}}}$, which is dominated by the fully-metallated repressor form, also possesses impeded unbinding kinetics—it was invisible for ${\rm{Zur}}_{{\rm{Zn}}}^{{\rm{mE}}}$ likely because its *K*_m_ is smaller than the low limit of accessible cellular protein concentrations (∼8 nM), but emerges after the D49A mutation, which further supports the importance of the salt bridges in Zur oligomerization and the impeded unbinding behaviors.

## DISCUSSION

We have uncovered that the Fur-family Zn^2+^-sensing transcription regulator Zur exhibits two unusual behaviors regarding regulator-chromosome interactions. First, the unbinding kinetics of both the repressor and non-repressor forms of Zur not only exhibit facilitated unbinding, a newly discovered phenomenon for a few DNA-binding proteins (26–28,51), but also show impeded unbinding, a *first-of-its-kind* phenomenon that likely results from Zur oligomerization on chromosome, helped by inter-dimer salt bridges. Overall, Zur has biphasic unbinding kinetics from chromosome with increasing cellular protein concentrations, drastically different from that protein unbinding being typically unimolecular processes whose first-order rate constants do not depend on the protein concentration. Second, the non-repressor form of Zur (e.g. C88S mutant), long-thought to have insignificant DNA affinity, can actually bind to chromosome tightly, likely at different locations from the consensus Zur box recognized by the repressor form of Zur. This tight chromosome binding by the non-repressor form suggests additional functional complexity beyond the typical regulator on-off model for transcription repression (or activation). It is worth noting here that analogously, *Campylobacter jejuni* Fur, in its apo-form, was shown to bind a DNA-motif distinct from that by its holo-form (52), and IscR, a member of the MarA/SoxS/Rob family of transcription regulators in *E. coli*, was shown to bind, in its apo non-repressor form, to DNA motifs different from its holo repressor form, while its apo-form was previously thought to have no significant binding to DNA (53,54).

To probe whether the biphasic unbinding of Zur occurs within the physiological cellular protein concentrations, we quantified cellular Zur^mE^ concentration when it is encoded only at the chromosomal locus. In minimal medium without added Zn, the cellular Zur^mE^, which has a significant population in the non-repressor form, ranges from ∼50 to 250 nM (mean = 119 ± 33 nM; Figure 4A). In this protein concentration range, the unbinding of non-repressor ${\rm{Zur}}_{{\rm{C88S}}}^{{\rm{mE}}}$ from TB sites is in the impeded regime and slows down by ∼42% from the lowest to the highest protein concentration (Figure 4B, blue points). When 20 μM Zn^2+^ is added, the cellular Zur^mE^, now largely in its repressor form, ranges from ∼60 to 300 nM (mean = 150 ± 48 nM; Figure 4A), reflecting an average of ∼28% protein concentration increase induced by Zn addition (Zur expression is also induced at the mRNA transcript level by the Zn addition; Supplementary Data Section 2.5). In this protein concentration range, ${\rm{Zur}}_{{\rm{Zn}}}^{{\rm{mE}}}$ is already in the facilitated unbinding regime, and its unbinding rate from a recognition site can increase by ∼36% (Figure 4B, red points).

**Figure 5.**
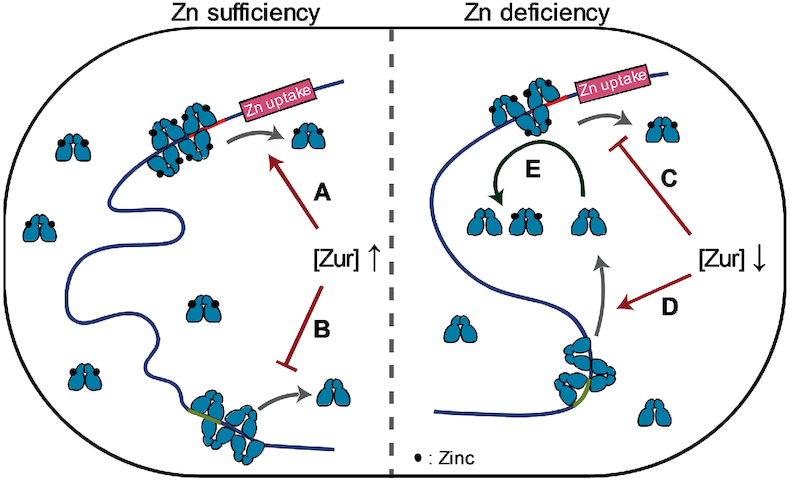
Functional model of repressor and non-repressor forms of Zur unbinding behaviors in *E. coli* upon encountering zinc sufficiency or deficiency. When zinc is sufficient, unbinding of the repressor form from operator site is facilitated (**A**) while that of the non-repressor form from storage site is impeded (**B**) due to higher cellular protein concentration. Upon zinc deficiency, the facilitated unbinding of the repressor form is attenuated (**C**) while the unbinding of the non-repressor form is less impeded (**D**) due to lower cellular protein concentration. Released non-repressors into cytosol could facilitate the repressor to unbind (**E**), helping transition to de-repression of zinc uptake.

Within the physiological protein concentration range, the opposite dependences of unbinding kinetics on the cellular protein concentration between non-repressor and repressor forms of Zur could provide functional advantages for an *E. coli* cell to repress or de-repress Zn uptake genes. When the cell is under Zn sufficient conditions that need strong repression of Zn uptake, the cellular concentration of Zur is higher and it is dominantly in the repressor form. The unbinding of repressor from operator sites could be facilitated by its increased concentration (Figure 5A), but the facilitated unbinding via direct substitution by another repressor has no functional consequence while facilitated unbinding via assisted dissociation will be immediately compensated by a rebinding of a repressor (the rebinding would occur within ∼0.11 s; Supplementary Data Section 7.2). For those cellular Zur in the non-repressor form, its unbinding from DNA is slowed, keeping them longer (i.e., stored) at non-consensus chromosomal sites (Figure 5B). On the other hand, when cell is under a Zn deficient environment that demands derepression of Zn uptake, the cellular Zur protein concentration is lower. Here unbinding of the repressor form would be slower (Figure 5C), which is undesirable for derepression, while the unbinding of the non-repressor form would be faster, releasing them from the non-consensus ‘storage’ sites on the chromosome into the cytosol (Figure 5D). If the cytosolic non-repressor form of Zur could possibly facilitate the unbinding of the repressor form from operator sites (e.g., through assisted dissociation), it would give a more facile transition to derepression. To support this possibility, we measured the apparent unbinding rate constant *k*_−1_ for chromosomally encoded ${\rm{Zur}}_{{\rm{Zn}}}^{{\rm{mE}}}$ in cells that contains a plasmid encoding an untagged non-repressor Zur_C88S_ mutant. When the expression of this Zur_C88S_ mutant is induced, *k*_−1_ of ${\rm{Zur}}_{{\rm{Zn}}}^{{\rm{mE}}}$ increases by ∼28% at any cellular ${\rm{Zur}}_{{\rm{Zn}}}^{{\rm{mE}}}$ concentration (Figure 4B, green vs. red points), indicating that non-repressor form of Zur can indeed facilitate the unbinding of repressor form from operator sites (Figure 5E).

Multivalent contacts with DNA, which underlie the facilitated unbinding, and significant interactions between proteins (e.g. via salt-bridge), which underlie Zur oligomerization and its impeded unbinding, are both common for protein-DNA and protein-protein interactions, respectively (25,27,29,30,51,55–61). For example, in *E. coli*, the binding of Fur (ferric uptake regulator), the prototype of Fur-family proteins, covers a large region on DNA (>100 bp including hexameric repeats (5′-NAT(A/T)AT-3′), mediated by lateral protein-protein interactions leading to oligomerization (62). Therefore, we postulate that the biphasic unbinding behavior from DNA discovered here for Zur could be broadly relevant to many other proteins in gene regulation.

## Supplementary Material

gkaa056_Supplemental_File
