# Supplementary material for: Biphasic unbinding of a metalloregulator from DNA for transcription (de)repression in Live Bacteria
Source: Nucleic Acids Res. 2020 Feb 3;48(5):2199–208. doi: 10.1093/nar/gkaa056 (PMC7049717; doi:10.1093/nar/gkaa056)
Supplement: gkaa056_Supplemental_File [file gkaa056_supplemental_file.pdf]

Supplementary information to

Biphasic Unbinding of a Metalloregulator from DNA for Transcription (De)repression in Live Bacteria

Won Jung[a], Kushal Sengupta[a], Brian M. Wendel[b], John D. Helmann[b], and Peng Chen[a,*]

[a] Department of Chemistry and Chemical Biology, Cornell University, Ithaca, New York 14853; and [b] Department of Microbiology, Cornell University, Ithaca, NY 14853
[*] Correspondence author: pc252@cornell.edu

Table of content

1. Construction of strains and plasmids 1
1.1 Tagging E. coli chromosomal genes with mEos3.2-FLAG (i.e., mE) via Lambda-Red 1
1.2 Making the Zur^{mE} gene fusions in the L-arabinose inducible plasmid pBAD24 1
1.3 Making the Zn-binding site B and salt-bridge removed mutants of Zur in pBAD24 2
2. Intactness and functionality of mE-tagged proteins in E. coli cells 3
2.1 Western blot shows that Zur^{mE} stays intact in the cell 3
2.2 RT (Reverse Transcription)-PCR shows that Zur^{mE} is functional and the mutant Zur^{mE}_{C88S} is a non-repressor in the cell 4
2.3 Validation of 20 μM Zn^{2+} as a zinc replete condition in M9 medium 5
2.4 Zur is a mixture of non-repressor and repressor forms in cells grown in the regular M9 medium. 5
2.5 20 μM Zn^{2+} induces Zur expression at mRNA transcript level 6
3. Sample preparation, single-molecule tracking (SMT) via stroboscopic imaging, and Single-cell quantification of protein concentration (SCQPC) 7
4. Determination of the minimal number of diffusion states and their fractional populations 11
4.1 Probability density function (PDF) and cumulative distribution function (CDF) of displacement length r per time-lapse 11
4.2 Assignments and validation of the three diffusion states of Zur. 12
4.3 Protein concentration dependence of fractional populations (A_{FD}, A_{NB}, and A_{TB}). 15
5. Determination of apparent unbinding rate constant k_{-1} from TB site 15
6. Mechanistic model of the biphasic concentration dependence of k_{-1}: impeded followed by facilitated unbinding 18
6.1 Cooperativity among oligomerized proteins can explain the impeded unbinding 19
6.2 Overall mechanistic model of Zur-DNA interactions in cells 22
7. Additional kinetic and thermodynamic parameters from the analysis of relative populations of FD, NB, and TB states 22
7.1 Analysis of relative populations of the FD, NB, and TB states using quasi-equilibrium model 22
7.2 Summary of extracted kinetic and thermodynamic parameters 26
7.3 Biphasic unbinding is not due to different sampling of various TB sites with increasing cellular protein concentrations 28
7.4 Validation of quasi-static system approximation for using a linear-combination of three-diffusion states 29
7.5 n_o dependence of extracted kinetic and thermodynamic parameters 29
8. Clustering analysis of residence sites 30
8.1 Pairwise distance distributions (PWDD) analysis of residence sites and the simulated random sites 30
8.2 Analysis of the fraction of residence sites within a threshold distance R to decouple the effect of chromosome condensation 31
9. Bootstrap of CDF analysis shows statistical reliability 32
10. List of abbreviations and strain names 33
Additional References 34

1. Construction of strains and plasmids

All plasmids, primers, and strains used are listed in Table S1, Table S2, and Table S3, respectively. Plasmids and genome extraction were performed using the QIAprep Spin Miniprep Kit (Qiagen), and Wizard Genomic Extraction Kit (Promega), respectively. PCR products as well as digested products including plasmids and inserts were recovered using the Wizard SV Gel and PCR Clean-Up System (Promega). The primers and all enzymes including restriction enzymes and ligase were purchased from the Integrated DNA Technologies and New England Biolabs, respectively. PCRs were performed using the AccuPrime Pfx DNA Polymerase Kit, and colony PCRs for screening were conducted using the Econo Taq DNA Polymerase Kit (Lucigen).

1.1 Tagging *E. coli* chromosomal genes with mEos3.2-FLAG (i.e., mE) via Lambda-Red

The Zur^{mE} strain was derived from the *Escherichia coli* BW25113 strain (CGSC# 7739 Keio Collection, Yale; genotype: (F-*Δ(araD-araB)567*, *ΔlacZ4787*(::rrnB-3), *λ-*, *rph-1*, *Δ(rhaD-rhaB)568*, *hsdR514*), and was generated by fusing the C-terminus of Zur with the monomeric, irreversibly photoconvertible fluorescent protein mEOS3.2 and FLAG tag for immunoblotting at the *zur* chromosomal locus via Lambda-Red homologous recombination technique.

For the linear DNA insert to tag chromosomal *zur* gene, primer pair H1-EZur-mEOS32-FP and H2-EZur-CAM-RP were used to obtain *H1-mEos3.2FLAG:cat-H2* via PCR from the *pUCmEOS3.2FLAG:cat* template. For the flanking homology regions (H1, and H2), H1 is the last 40 bp of the *zur* gene before the stop codon and H2 is the next 40 bp after the *zur* stop codon.

Transformation of the linear DNA insert into *E. coli* cells was performed via electroporation. We first prepared the electrocompetent *E. coli* BW25113 cells harboring the temperature-sensitive pKD46 plasmid. The SOB media [2% w/v Bacto Tryptone (Sigma-Aldrich, cat. #: T9410), 0.5 % w/v Bacto Yeast Extract (Sigma-Aldrich, cat.#:Y1625), 10 mM NaCl (Macron, 7581-12), 2.5 mM KCl (Fisher Scientific, P217-500), 10 mM $MgCl_2$ (Mallinckrodt, 5958-04), and 10 mM $MgSO_4$ (Fisher Scientific, M63-500) all in nanopure sterile water] containing ampicillin (100 µg/mL, USBiological) and 20 mM L-arabinose (Sigma-Aldrich, cat. #: A3256), which is a reagent that can induce the expression of the *bet*, *gam*, and *exo* **λ**-Red enzymes encoded in pKD46, was used for culturing. The cells were centrifuged and then washed twice with cold 10% glycerol (Macron, 5092-02) in nanopure sterile water. The cells were diluted to a final volume of 50 µL in 10% glycerol in nanopure sterile water. The linear DNA insert was then electroporated (2.5kV, using MicroPulser Elctroporator; cat.#: 1652100, Bio-Rad) into the prepared electrocompetent cells expressing the recombinase enzymes (exo, β, γ) from pKD46, and then recovered in SOC medium [SOB medium + 20 mM glucose (sigma-Aldrich, cat. #: G7528)]. After 4 hours incubation at 37 °C, the cells were plated onto LB-agar containing chloramphenicol (10 µg/mL, USBiological), and further incubated for 18 hours.

Successful transformation (i.e., insertion of *mEos3.2FLAG:cat* at the chromosomal target) was confirmed by colony PCR screening and gene sequencing. The temperature-sensitive pKD46 plasmid after homologous recombination was eliminated by incubating the cells at 42 °C for 18 h, which was confirmed by ampicillin-sensitivity. This resulting engineered strain containing *zur-mEos3.2-FLAG-cam* in genome is called Zur^{mE}.

1.2 Making the Zur^{mE} gene fusions in the L-arabinose inducible plasmid pBAD24

The gene of *zur-mEos3.2-FLAG* was cloned out from the purified genome of Zur^{mE} as a template, with primer pair EcoRI-EZur-pB24fp and Sal1-FLAG-mEOS32-pB24rp (Table S2). The copied PCR product was digested with EcoRI-HF and SalI-HF restriction enzymes and inserted into similarly digested pBAD24 plasmids using Quick Ligase enzyme to generate pZur_mE (Table S1). The plasmids were transformed into the cloning strain E. cloni 10G (Lucigen). Successful transformation was confirmed by antibiotic (100 µg/mL ampicillin) selection on a LB-agar plate, and insertion was screened by colony PCR and confirmed by DNA sequencing. Extracted pZur_mE was transformed into the Δ*zur* strain (JW5714-1

from Keio collection) giving DZ-pZurmE (Table S3). The Δ*zur* strain (not Zur^{mE}) was chosen as the base strain for plasmid expression to avoid the complexity that the chromosome and plasmid express different forms of Zur (i.e., Plasmid insert could be a mutant form, e.g., site B (zinc binding site) mutant or salt-bridge removed form).

1.3 Making the Zn-binding site B and salt-bridge removed mutants of Zur in pBAD24

Site-directed mutagenesis was performed using QuikChange mutagenesis kit (Stratagene) to make non-repressor and salt-bridge removed mutant form of Zur in pBAD24. We mutated a cysteine in the regulatory Zn binding site to serine (C88S) to make the non-repressor form of Zur, pApoZur_mE (note the "Apo" in the plasmid names refers to the C88S mutation), and an aspartate which is responsible for salt-bridge formation between dimers to alanine (D49A) to make the salt-bridge removed mutant of Zur, pZurD49A_mE(1). Primer pair (EZurC88S-fp and EZurC88S-rp for site B-mutant, and EZurD49A-fp and EZurD49A-rp for salt-bridge removed mutant) was used together with pZur_mE plasmid as the template (Table S1, Table S2). Sequential mutations were conducted to achieve double mutations (that is, C88S/D49A). Dpn1 digestion (NEB, R0176S) was performed to remove the methylated nonmutated parental plasmid. The plasmid containing the mutant-form of Zur was transformed into E. cloni 10G strain for propagation. Extracted mutant plasmids were confirmed by DNA sequencing. The sequence-confirmed plasmid containing pApoZur_mE, pZurD49A_mE, or pApoZurD49A was transformed into the Δ*zur* strain JW5714-1, resulting in DZ-pApoZurmE, DZ-pZurD49AmE, and DZ-pApoZurD49AmE strains, respectively (Table S3).

Table S1. Plasmids used or constructed in this study (note that "Apo" in the plasmid name refers to the C88S mutation that makes Zur into non-repressor form)

Plasmid name	Relevant characteristics, or insert	Source
pKD46	bet, gam, exo enzymes	Datsenko et al(2)
pBAD24	Base plasmid, L-arabinose inducible	Guzman et al(3)
pUCmEos3.2FLAG:cat	*mEos3.2* : *chloramphenicol*	Chen et al(4)
pZur_mE	*Zur-mEos3.2-FLAG*	This study
pApoZur_mE	*Zur(C88S)-mEos3.2-FLAG*	This study
pZurD49A_mE	*Zur(D49A)-mEos3.2-FLAG*	This study
pApoZurD49A_mE	*Zur(C88S,and D49A)-mEos3.2-FLAG*	This study

Table S2. Primers used in this study

Primer name	5'→3'
H1-EZur-mEOS32-FP	CCAGCATGATCACTCTGTGCAGGTGAAAAAGAAACCGCGTATGAGTGCGATTA AGCCAGA
H2-EZur-CAM-RP	TAATCCCTCCTGCCCGACGTGTACAAGGATGTACGCCCTCCGACGGCCAGTGAA TTCGA
EcoRI-EZur-pB24fp	CAGGAG GAATTCACCATGGAAAAGACCACAACGCA
Sal1-FLAG-mEOS32-pB24rp	AGTCAGGTCGACTTATTTATCATCATCATCTTTATAATCAGGACGTCGTCTGGCAT TGTC
EZurC88S-fp	CAGTTATGTGCTCTCTCATCTGTTCGATC
EZurC88S-rp	GATCGAACAGATGAGAGAGCACATAACTG
EZurD49A-fp	ATGATCTGCTTGCTTTACTGCGCG

EZurD49A-rp	CGCGCAGTAAAGCAAGCAGATCAT
Zur-rtpcr-fp	GGAGTTATTAGCGCAGGC
Zur-rtpcr-rp	GCCATCCTGCAGACTCAT
zinT18-fp	CAAACTGGCTGTTGCTTTAGG
zinT104-rp	TCTGTTAAGGGTTTGCCGTG
znuC220_up	CAGAAGCTGTATCTCGACACC
znuC297_dn	TTCTTTATGTGTACCAGGGCG

Table S3. Strains used or constructed in this study

Strains	Plasmid	Chromosomal Gene modification	Reference or source
BW25113	pKD46	Base strain	Keio collection
Zur^{mE}	none	Zur-mEos3.2-FLAG	This study
JW5714-1	none	Δ*zur*	Keio collection
DZ-pZurmE	pZur_mE	Δ*zur*	This study
DZ-pApoZurmE	pApoZur_mE	Δ*zur*	This study
DZ-pZurD49AmE	pZurD49A_mE	Δ*zur*	This study
DZ-pApoZurD49AmE	pApoZurD49A_mE	Δ*zur*	This study

2. Intactness and functionality of mE-tagged proteins in *E. coli* cells

2.1 Western blot shows that Zur^{mE} stays intact in the cell

We performed Western blot to check the intactness of the fusion protein (that is, Zur-mEos3.2-FLAG) in the cell. We detected the FLAG epitope (RPDYKDDDDK) at the C-terminal of the fusion protein, and anti-FLAG antibody was used for immunoblotting.

The DZ-pZurmE strain which could express Zur^{mE} from the pBAD24 inducible by L-arabinose, and a negative control strain DZ-pBAD containing the parent pBAD24 without insert were grown overnight (18 hr) in 6 mL LB with appropriate antibiotics. 50 µL of the samples were further grown to OD_{600} of 0.4 in 5 mL M9 medium with amino acids (8% v/v 50x GIBCO), vitamins (4% 100x GIBCO), glycerol (0.4%). L-arabinose was added to the final concentration of 1 mM and the culture was further incubated for 20 min to induce the plasmid expression. For the additional negative controls, samples without L-arabinose induction were prepared. 1 mL aliquots of the resulting cell cultures were collected by centrifugation, and the cell pellets were re-suspended in 200 µL 2X SDS lysis buffer. The lysed samples were run in SDS-PAGE with ECL Plex fluorescent rainbow protein molecular weight markers (GE Healthcare Life Science) in 1X MES buffer, and then transferred onto the Hybond-LEP PVDF membrane (GE Healthcare Life Sciences). The transferred membrane was blocked with 4% Amersham ECL Prime blocking reagent (GE Healthcare Life Sciences) in PBS-T (0.1% Tween-20, Sigma-Aldrich) wash buffer while shaking at RT for 2 hr. After blocking, the membrane was washed with PBS-T twice, and incubated with rabbit-derived anti-FLAG primary antibody (1:10,000 dilution, Rockland Immunochemical) for 2 hr. The membrane was rinsed with PBS-T 4 times and PBS buffer 3 times. The goat-derived Horseradish Peroxidase-conjugated Fab fragment anti-rabbit antibody (1:5,000 dilution, Rockland Immunochemical) was used as the secondary antibody, which could be probed with Pierce ECL 2 Western Blotting substrate (Fisher Scientific). Bio-Rad ChemiDoc MP Imaging System was used to detect peroxidase activity.

One dominant band of L-arabinose induced Zur-mEos3.2-FLAG was observed at MW ~ 46 kDa (that is, MW of Zur + MW of mEOS3.2-FLAG), and no discernable band was observed at MW ~ 27 kDa which is expected to be the MW of mEOS3.2-FLAG (Figure S1A). Therefore, the Zur-mEos3.2-FLAG is intact in the cell.

2.2 RT (Reverse Transcription)-PCR shows that Zur^{mE} is functional and the mutant $\mathrm{Zur}^{\mathrm{mE}}_{\mathrm{C88S}}$ is a non-repressor in the cell

RT-PCR assay was performed to measure the mRNA levels of *zur* regulons, which could directly reflect the functionality (i.e., repression ability) of the fusion protein. The *znuC* gene encoding the zinc uptake system that is weakly repressed by Zur and periplasmic zinc trafficking protein *zinT* that is strongly repressed by Zur, respectively, were chosen as the reference *zur* regulons(1,5).

The wild-type (BW25113), *zur*^{mE} (which expresses Zur^{mE} from *zur*'s chromosomal locus), Δ*zur*, DZ-pZurmE (which expresses Zur^{mE} from a plasmid in a Δ*zur* strain), and DZ-pApoZurmE (which expresses the non-repressor mutant $\mathrm{Zur}^{\mathrm{mE}}_{\mathrm{C88S}}$ from a plasmid in a Δ*zur* strain) strains (Table S3) were grown overnight (18 hrs) in 6 mL LB with appropriate antibiotics. 50 μL of the sample were further grown to OD_{600} of 0.4 in 5 mL LB or M9 imaging media, and then final concentration of 1 mM L-arabinose (and a final 20 or 200 μM $ZnSO_4$ if needed for zinc supply) was added to induce (if applicable) the plasmid expression for 30 min. Cells were washed and collected by centrifugation. Total RNA was purified using PureLink® RNA Mini Kit (Life Technologies) and quantified by NanoDrop spectrophotometer (Thermal Scientific) for normalization. Purified RNAs were converted to the cDNA using SuperScript® III First-Strand Synthesis SuperMix kit (Life Technologies) with random hexamer primers. The primer pair, zinT18-fp and zinT104-rp for *zinT* gene, or znuC220_up and znuC297_dn for *znuC,* was used for RT-PCR(1) (Table S2). The mRNA levels of *zinT* and *znuC* were assessed relative to that of the housekeeping gene (16S rRNA) as an internal reference(6). The samples were measured in triplicate in an optical 96-well plate (Life Technologies) in a reaction mixture with the SYBR Green reagent (Life Technologies) and imaged using Applied Biosystems (Thermo Fisher) Viia7 Sequence Detection System. Fluorescence was detected at the annealing phase in RT-PCR. The threshold cycles (C_T) were calculated using Viia7 software.

The relative expressions of *znuC* and *zinT* were plotted in Figure S1B-D using the comparative C_T method(7). In LB, a nutritionally rich medium, the transcription of *zinT* in the WT strain is repressed to ~22% of that of the Δ*zur* strain in the absence and presence of 200 μM Zn^{2+} in solution (Figure S1B, columns 1 vs 2, and 6 vs 7). The lost repression ability of Δ*zur* was recovered by plasmid expressed Zur^{mE} (Figure S1B, column 3 vs. 2), whereas the non-repressor mutant $\mathrm{Zur}^{\mathrm{mE}}_{\mathrm{C88S}}$ (i.e., C88S) could not repress as much as the wild-type strain (the *zinT* expression was 82% of that of Δ*zur* strain; Figure S1B, column 4 vs. 1 and 2), indicating that the plasmid expressed Zur^{mE} is functional and that the mutation C88S could remove the ability of Zur^{mE} to repress – the latter was expected from the previous report by Gilston et al(1). The same assay on the chromosomally tagged Zur^{mE} strain shows it has ~84% ($= \frac{[\mathrm{mRNA}]_{\Delta zur} - [\mathrm{mRNA}]_{zur^{mE}}}{[\mathrm{mRNA}]_{\Delta zur} - [\mathrm{mRNA}]_{\mathrm{WT}}} \times 100\%$) repression compared with the wild-type (Figure S1B, column 5 vs. 1), further supporting that Zur^{mE} is functional.

In the M9 imaging medium, with added Zn (20 or 200 μM Zn^{2+}), the transcription of both *znuC* and *zinT* of the WT strain was repressed to ~10-15% of that of the Δ*zur* strain (Figure S1C and D, columns 1b-c *vs.* 2b-c). Similarly, the lost repression ability of the Δ*zur strain* was recovered by plasmid expressed Zur^{mE} (Figure S1 C and D, columns 3b-c *vs* 2b-c), however the $\mathrm{Zur}^{\mathrm{mE}}_{\mathrm{C88S}}$ strain was unable to recover the repression (*znuC* and *zinT* expression was 80-85% of those of Δ*zur* strain) (Figure S1C and D, columns 4b-c *vs*. 2b-c). Chromosomally tagged Zur^{mE} also showed repression similar to the WT (Figure S1C and D, columns 5b-c *vs*. 1b-c). Altogether, these results indicate that Zur^{mE} is a functional repressor under Zn stress and the mutant $\mathrm{Zur}^{\mathrm{mE}}_{\mathrm{C88S}}$ is a non-repressor.

2.3 Validation of 20 µM Zn^{2+} as a zinc replete condition in M9 medium

In our imaging experiment, we used a concentration of 20 µM Zn^{2+} in the medium as the zinc replete condition and expected that the Zur proteins in the cell would be predominately in their Zn-metallated form (i.e., repressor-form). In this section, we used the RT-PCR assay with various zinc levels in the media and zinc quantification method to validate that 20 µM Zn^{2+} could indeed result in sufficient Zn-metallation of the Zur protein in the cell.

We first determined the actual Zn^{2+} concentration in various media using ICP-MS or a Zinc quantitation kit (Zinc Assay kit, Sigma-Aldrich, cat. #: MAK032) (Table S4). In ICP-MS, the samples were diluted in 2% HNO_3 and the total metal ions were analyzed by external calibration using a Perkin-Elmer ELAN DRC II ICP-MS. Gallium was used as an internal standard. In the zinc quantitation kit, the 50 mM of zinc standard solution and its serial dilutions were used for calibration. All samples were treated with TCA solution to free all bound zinc. Samples and standard zinc solution were prepared in the optical 96 plates (Costar) and measured the absorbance at 560 nm using the Synergy HT multi-detection microplate reader (Bio-tek). The results are summarized in (Table S4).

Table S4. Total Zn concentration quantitation in different media

	**[Zn] (µM)**	
**Sample**	**from ICP-MS**	**from the assay kit**
LB	9.7 ± 0.8	19.6 ± 2.3
Regular M9	0.047 ± 0.013	< 0.28
M9 imaging medium [a]	0.058 ± 0.017	
Chelexed M9 [b]	0.008 ± 0.002	

[a] Regular M9 medium supplemented with amino acids, vitamins, and 0.4% glycerol. See section 3 for the details of the supplements. [b] Chelexed M9 is the regular M9 medium that has been treated through Chelex resin (8) to remove Zn as much as possible; this medium was not used in this study but provided here as a comparison.

We measured levels of the same transcripts described in Section 2.2 with 20 µM of Zn^{2+} in the M9 imaging medium and compared the results with those at a very high concentration of zinc (200 µM). The transcript levels of both *zinT* and *znuC* genes in all relevant strains [i.e., WT, *zur*^{mE}, DZ-pZurmE (plasmid encoded Zur^{mE} in a Δ*zur* strain)) with 20 µM of Zn^{2+} in the M9 media were all comparable to those with 200 µM of Zn^{2+} in the M9 media (Figure S1C-D, columns 1b vs 1c, 3b vs 3c), all showing strong repression compared with the Δ*zur* strain (Figure S1C-D, columns 2b-c). Therefore, 20 µM of Zn^{2+} in the M9 imaging media can evoke strong repression of *zur* regulons, and this repression is already saturated (i.e., no further repression when Zn^{2+} concentration increases to 200 µM), supporting that under 20 µM Zn^{2+} in the M9 medium, Zur protein in the cell is predominantly in the metallated repressor form.

We further measured *zur* regulon transcript level (i.e., *zinT*) in the rich medium LB in the absence and presence of added 200 µM Zn^{2+} (Figure S1B). Compared with the Δ*zur* strain, *zinT* transcription is as repressed in LB as that in LB + 200 µM Zn^{2+} (Figure S1B, columns 1-2 and 6-7). This repression in LB comes from that LB already contains ~9.7 µM Zn^{2+} (Table S4). The fact that the added 200 µM Zn^{2+} in LB did not increase the repression level further support that adding 20 µM Zn^{2+} in M9 medium is sufficient to predominantly metallate Zur in the cell to evoke its regulon repression.

2.4 Zur is a mixture of non-repressor and repressor forms in cells grown in the regular M9 medium.

As discussed above, in WT cells grown in the presence of added 20 µM Zn^{2+} and further to 200 µM in the M9 medium, the transcription of *znuC,* a weakly repressed regulon of Zur, minimizes down to ~10% of that of the Δ*zur* strain (i.e., maximal repression; Figure S1C, columns 1b-c vs. 2b-c). In contrast,

in regular M9 imaging medium, the transcription of *znuC* is only partially repressed, at ~32% of that of the Δ*zur* strain (Figure S1C, column 1a vs. 2a). This partial repression of *znuC* indicates that in regular M9 medium, Zur proteins in the cell are not fully in the metallated repressor form and some fraction are still in the metal-deficient non-repressor form.

The transcription of *zinT*, a strongly repressed regulon of Zur, is already maximally repressed to ~10% in the WT strain in the regular M9 medium (which contains residual amount of Zn; Table S4), compared with that in the Δ*zur* strain (Figure S1D, column 1a vs 2a), and adding 20 or 200 μM Zn^{2+} does not further increase repression (Figure S1D, column 1b-c). This behavior of *zinT* results from the tighter binding of Zur repressor form to the *zinT* promoter, so even some cellular Zur protein are still in the non-repressor form, the available repressor form fraction of Zur could already evoke maximal repression of *zinT*. This is consistent with past studies(1) that Zur exhibits hierarchical repression on *zinT* and *zunC* (i.e., *zinT* > *znuC*).

2.5 20 μM Zn^{2+} induces Zur expression at mRNA transcript level

The expression of *zur* in zinc-dependent manner exhibits diversity in different organisms. In *S. coelicolor*(9) and *M. tuberculosis*(10,11), *zur* expression is upregulated with zinc level at the transcription and translation level, respectively, whereas in *E. faecalis*(12) and *P. aeruginosa*(13), *zur* is downregulated at the transcription and translation level, respectively. In contrast, auto-regulation of Zur was not observed in *Bacillus subtillis*(14). The zinc-dependent downregulation or upregulation of *zur* is due to direct autoregulation by Zur itself or an indirect regulation by some other regulators.

The expression of *E. coli* Zur in a zinc-dependent manner had not been reported previously. Here RT-PCR assay was performed to measure the mRNA levels of *zur*. The experimental procedure was exactly similar to the SI Section 2.2. The primer pair used for this study are Zur-rtpcr-fp and Zur-rtpcr-rp (Table S2). In the M9 imaging media (Figure S1E), adding 20 μM Zn^{2+} (or 200 μM) led to the increase in the *zur* mRNA level.

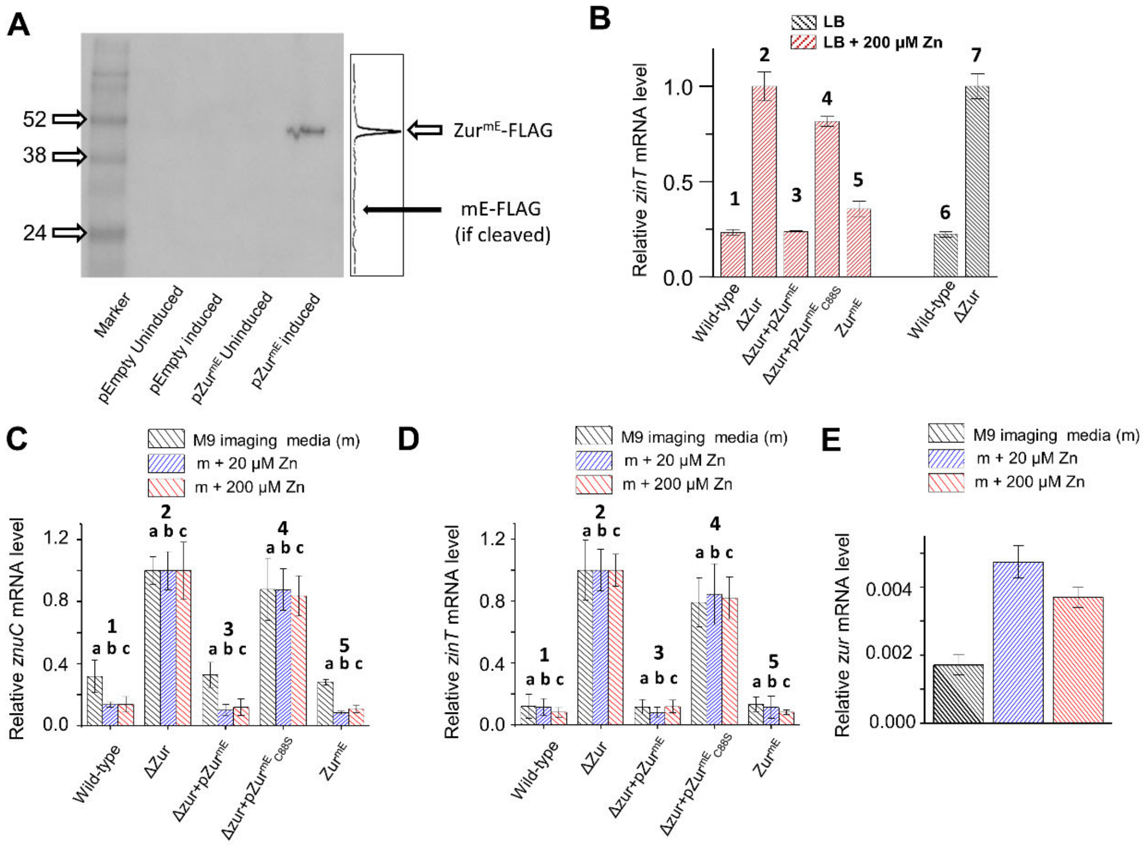

Figure S1. Intactness and functionality assay of Zur^{mE}. (A) Western blot of FLAG-tagged Zur^{mE} demonstrated that Zur^{mE} is intact in the cell. Only Zur^{mE} expressed from a pBAD24 plasmid with L-arabinose induction was detectable (5th column). A line profile of 5th column is shown to the right of the gel image. No discernable bands were observed in the negative controls, which are the parent empty pBAD24 (without the *zur*^{mE} insert; column 2-3) and un-induced pZur^{mE} (column 4). The expected size of Zur^{mE} is ~ 46 kDa (including the FLAG tag), and no cleavage product was observed (5th column). (B) RT-PCR assay for transcription level of *zur* regulon (*zinT*) demonstrated that Zur^{mE} is functional. Columns 1-5 were conducted in 200 µM of Zn^{2+} in LB while columns 6-7 were conducted without zinc stress in LB medium. All mRNA levels were normalized to that of the Δ*zur* strain. (C, D) RT-PCR assay for transcription levels of *zur* regulon *znuC* (C) and *zinT* (D) in the imaging M9 medium in the absence or presence (20 µM or 200 µM) of Zn^{2+}. All mRNA levels were normalized to that of the Δ*zur* strain. *(E)* RT-PCR assay for transcription level of *zur* in wild-type *E. coli* cells.

3. Sample preparation, single-molecule tracking (SMT) via stroboscopic imaging, and Single-cell quantification of protein concentration (SCQPC)

Sample preparation: For imaging experiments, strains were first streaked onto LB plates with appropriate antibiotics. Single colonies were inoculated and grown in LB medium overnight at 37 °C. The overnight cultured was diluted 1:100 in M9 medium supplemented with amino acids, vitamins, and 0.4% glycerol, and further grown to OD_{600} of 0.3. L-arabinose was added to induce plasmid expression for 0 - 20 min when applicable. $ZnSO_4$ solution was also added (for Zn stress) into the cell media to a final concentration of 20 µM. 2 mL of the cell culture was centrifuged and washed twice with the same M9 media, and further incubated at 37 °C for 30 - 60 min to help maturation of the mEos3.2 tag. Cell was collected by centrifugation and then immobilized on an agarose pad between a coverslip and a slide, and

sealed with epoxy-glue (Figure S2A). The coverslip was pre-treated with gold particle (100 nm) as position markers for stage-drift correction.

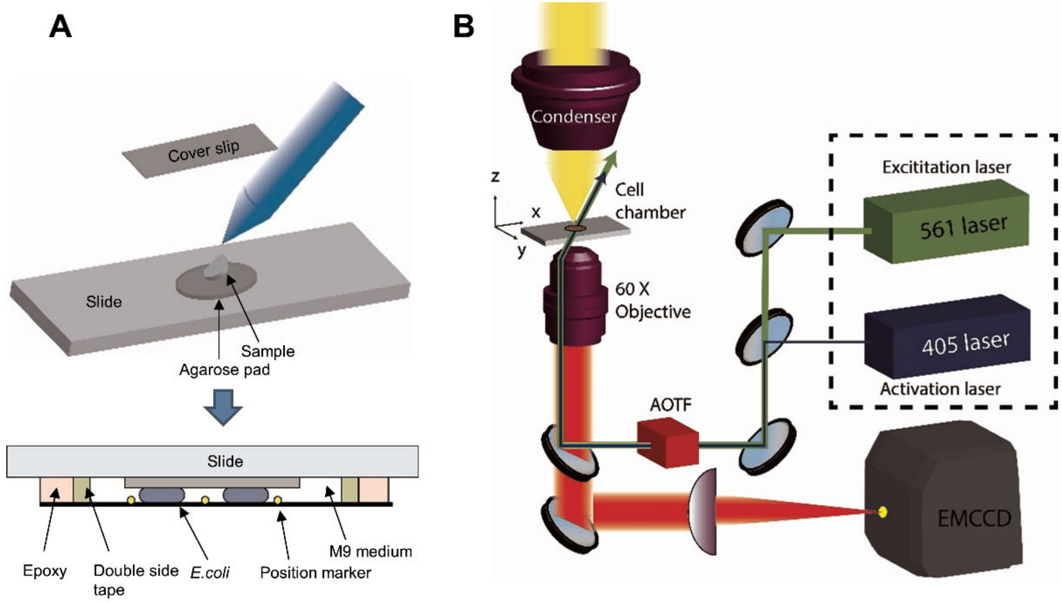

Figure S2. Slide preparation for imaging, and schematic diagram of the microscope setup. (A) *E. coli* cells were immobilized on 3% agarose gel pad. The sample on the pad was sandwiched between a glass slide and a coverslip which is pre-casted with gold nanoparticles as position markers, and sealed by double-sided tape and epoxy. (B) The 405-nm laser was used to photoconvert mEos3.2 to its red-fluorescent form, and the 561-nm laser was used to excite and track the red mEos3.2. An AOTF was synchronized with EMCCD camera. Figure adapted from the reference (4).

SMT and SCQPC. The procedure of SMT via stroboscopic imaging(15-20) and SCQPC were described in our previous work (Figure S2B)(4). For SMT, the cells were first illuminated with 405-nm laser for 20 ms to photoconvert a single mE-tagged protein, in which the laser intensity was tuned low enough (1-10 W/cm^2) to photoconvert one or none protein at a time. Then samples were illuminated with 30 pulses of 561-nm laser with 4 ms pulse duration and time lapse T_{tl} = 40 ms to image photoconverted mEos3.2 red fluorescence. The EMCCD camera exposure is slow, but synchronized with the 561 nm pulses. This stroboscopic imaging allowed us to obtain diffract-limited images of mobile molecules, whereas the time lapse was chosen fast enough to follow dynamic protein-DNA interactions. This photoconversion and imaging scheme was repeated for 500 cycles for each cell. An exemplary fluorescence image is shown in Figure 1A.

After the SMT step, SCQPC was carried out. Here the cells were illuminated with 405-nm laser for 2 min to photoconvert all remaining un-photoconverted mEos3.2 proteins to the red form, and the whole cell red fluorescence was imaged using 561-nm laser for 3000 frames at the same power density as the SMT imaging step. This step was repeated twice. The protein copy number inside cells were then determined by dividing the total cell fluorescence by the average fluorescence of a single mEos3.2, which was pre-determined from the earlier SMT. The camera EM-gain was adjusted during the SCQPC step to avoid saturation and remain in the linear dynamic regime of camera sensitivity, and this adjustment was used to correct for the actual recorded camera counts. The total copy number of protein in each cell was estimated using Equation S[1]:

$$N_{copy} = \frac{N_{\mathrm{SMT}} + N_{\mathrm{SCQPC}}}{PE_{\mathrm{mE}}\, OS_{\mathrm{Protein}}} \qquad \mathrm{S[1]}$$

where PE_{mE} is the photoconversion efficiency of mEos3.2(21,22) (= 0.42), OS_{protein} is the oligomeric state of protein (Zur is homodimer, therefore $OS_{\mathrm{protein}} = 2$), and N_{SMT} and N_{SCQPC} are mEos3.2 copy numbers determined in the SMT and SCQPC steps, respectively.

A custom-written MATLAB software called iQPALM (Image-based Quantitative Photo-Activated Localization Microscopy) was used to determine the centroid location of the candidate red fluorescence spots of individual mEos3.2 proteins. We first determined the cell boundary using the bright field optical transmission image. We further selected the cells with length of 2.7 ± 0.9 μm to decrease the possible contamination from dividing cells which potentially have more than one copy of chromosome (Figure S3, 2nd column). Then the cell boundaries in the region of interest (ROI) were superimposed onto the corresponding fluorescence image to select out the candidates of single-molecule fluorescence within the cell boundaries. The centroid of each candidate was determined by fitting the fluorescence spot with a 2-dimensional Gaussian function in Equation S2.

$$I(x,y) = A\exp\left[-\frac{(x-x_0)^2}{2\sigma_x^{\,2}} - \frac{(y-y_0)^2}{2\sigma_y^{\,2}}\right] + B \qquad \text{S[2]}$$

where $I(x,y)$ is the fluorescence intensity of the candidate at position (x,y), and A, B, (x_o, y_o), and σ_i are the amplitude, background, centroid, and standard deviation of i-direction of the Gaussian function. And the localization precision of its centroid was estimated in Equation S[3] and Equation S[4] (23,24).

$$Err_i = \sqrt{\frac{\sigma_i^{\,2}}{N} + \frac{a^2}{12N} + \frac{8\pi\sigma_i^{\,2}b^2}{a^2N^2}} \qquad \text{S[3]}$$

$$N = \frac{(cts/g)\times(S/QE)\times 3.65}{E_{hv}} \qquad \text{S[4]}$$

Here N is the number of detected photons, a is the pixel size, and b is the standard deviation of the background. cts, g, S, and QE are the total EMCCD counts of the fitted spot, the EM gain (in linear scale, unitless), sensitivity (electrons per count), and quantum yield (unitless) of the EMCCD camera in the spectral range of detected fluorescence, respectively, provided by Andor Technology. The value of 3.65 is a physical constant for electron creation in silicon (eV per electron) and E_{hv} (in eV) is the energy of a single detected fluorescence photon (chosen at wavelength 584 nm, the peak of mEos3.2 red fluorescence spectrum).

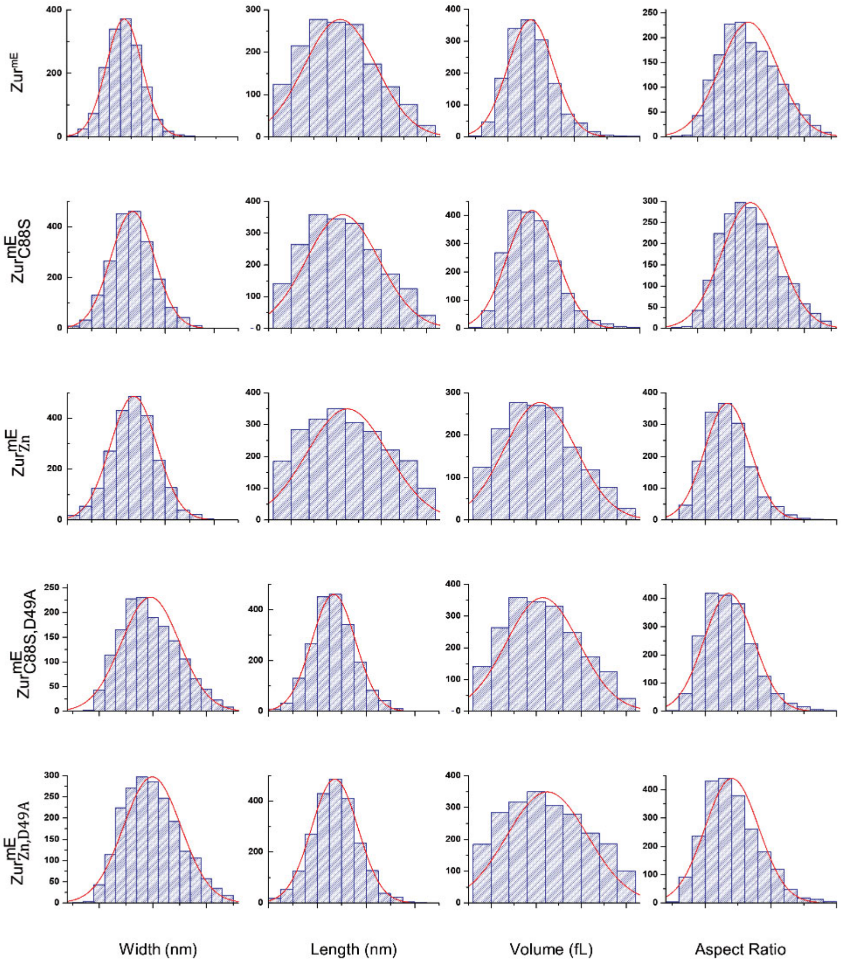

Figure S3. Distribution of cell geometric parameters. Width and length were obtained from the optical transmission image, and the cell volume was approximated to that of a model geometry in which cell shape in the transmission image was fitted with a cylinder with two hemispherical caps as described in our previous work(4). Each histogram is fitted with a Gaussian function (red curve).

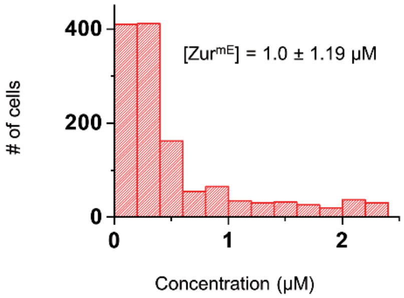

Figure S4. An example of the experimentally sampled distribution of the Zur^{mE} protein concentration among individual cells expressed from a pBAD24 plasmid. Expression level could be controlled by L-arabinose induction level. More experiments were performed on cells having lower protein concentrations so as to achieve sufficient statistics (the number of trajectories per cell obtained is less for cells having lower cellular protein concentrations). The average concentration with standard deviation is denoted in the panel.

4. Determination of the minimal number of diffusion states and their fractional populations

4.1 Probability density function (PDF) and cumulative distribution function (CDF) of displacement length *r* per time-lapse

The probability density function of displacement vector $\vec{r}$ at time t for N-dimensional Brownian diffusion is:

$$P_N(\vec{r},t)\mathrm{d}\vec{r} = \left(\sqrt{\frac{1}{4\pi Dt}}\right)^N \exp\left(-\frac{\vec{r}_i^{\,2}}{4Dt}\right)\mathrm{d}\vec{r} \qquad \text{S[5]}$$

where D is the diffusion constant. Then, the probability density function of the scalar displacement length r, PDF(r, t), is obtained by integrating $P_N(\vec{r},t)$ over all angular spaces (that is; $\mathrm{d}x\mathrm{d}y \Rightarrow 2\pi r\mathrm{d}r$ for 2D, and $\mathrm{d}x\mathrm{d}y\mathrm{d}z \Rightarrow 4\pi r^2\mathrm{d}r$ for 3D).

$$\mathrm{PDF}_{2\mathrm{D}}(r,t) = \frac{r}{2Dt}\exp\left(-\frac{r^2}{4Dt}\right) \qquad \text{S[6]}$$

$$\mathrm{PDF}_{3\mathrm{D}}(r,t) = \frac{r^2}{Dt\sqrt{4\pi Dt}}\exp\left(-\frac{r^2}{4Dt}\right) \qquad \text{S[7]}$$

The corresponding cumulative distribution function (CDF) of displacement length r is obtained by $\mathrm{CDF}(r,t) = \int_0^r \mathrm{PDF}(l,t)\mathrm{d}l$.

$$\mathrm{CDF}_{2\mathrm{D}}(r,t) = 1 - \exp\left(-\frac{r^2}{4Dt}\right) \qquad \text{S[8]}$$

$$\mathrm{CDF}_{3\mathrm{D}}(r,t) = \mathrm{erf}\left(\sqrt{\frac{r^2}{4Dt}}\right) - \sqrt{\frac{r^2}{\pi Dt}}\exp\left(-\frac{r^2}{4Dt}\right) \qquad \text{S[9]}$$

Fitting the histogram of displacement length r with PDF(r; t) or the cumulative distribution of r CDF(r; t) will both give the diffusion constant D. However, there is a practical advantage in using CDF than PDF. Using PDF analysis, the choice of the bin size in generating the histogram of r needs to be carefully evaluated, as it may affect the fitting outcome, while the cumulative distribution does not involve binning. Therefore, we chose to fit the CDF rather than PDF. Also, since we tracked the molecules in 2-D plane, we used the 2-D versions of PDF and CDF for next subsequent data analysis (e.g. Equation S[6] and S[8]).

Therefore, the effective diffusion constants and the fractional populations of corresponding states were extracted by analyzing CDF of displacement length r per time-lapse (T_{tl} = 40 ms). Experimentally, only the first displacement of each tracking trajectory was used for constructing CDF to avoid potentially biasing the sampling toward relatively long trajectories(25).

Fits were performed using least-square fitting method in MATLAB, and quality of the fit was assessed by residual analysis. A single component model could not satisfactorily fit the data (Figure S5A), indicating more than one diffusion state are present. We therefore used a linear combination of multiple diffusive terms for the CDF(r) (Equation S[10] and fitted the data until the residual falls within a satisfactory range (i.e., within 95% confidence level). Note that linear combination of CDFs assumes a quasi-static system as an approximation, which means the time-resolution of experimental observation should be fast

enough to sample each diffusion state (i.e., sampling is faster than the interconversion rates between the states). We later obtain interconversion rates between states and verify this approximation (Section 7.4).

$$\mathrm{CDF}(r) = \sum_i A_i \left(1 - \exp\left(-\frac{r^2}{4D_i T_{tl}}\right)\right) \qquad \mathrm{S}[10]$$

Here, D_i and A_i are the diffusion constant of i-th state and its fractional population, respectively, and $\sum_i A_i = 1$. The residual analysis of CDF indicated that three components in Equation S[10] were needed to satisfactorily fit the data, which means that three diffusion states are *minimally* necessary to characterize the mobility of Zur inside a cell (Figure S5C).

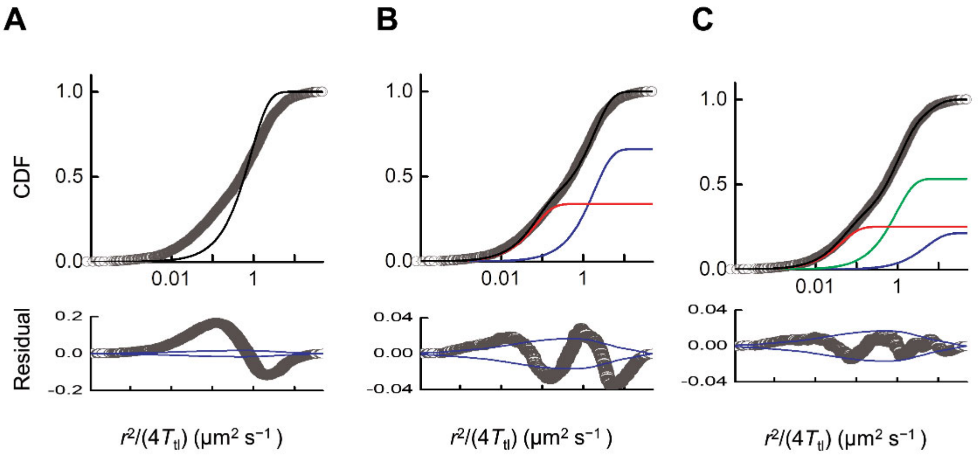

Figure S5. Upper panels are the CDFs of r (plotted against $\frac{r^2}{4T_{tl}}$) of $\mathrm{Zur}^{\mathrm{mE}}_{\mathrm{C88S}}$ at $[\mathrm{Zur}^{\mathrm{mE}}_{\mathrm{C88S}}]_{\mathbf{cell}}$ = 414 ± 33 nM fitted with (A) one, (B) two, and (C) three diffusion-state models. The grey circles are the experimental CDF data. The colored lines are individual components of CDF(r). The black line is the overall fit. Lower panels are residues of the CDF fits. Blue dashed lines are the 95% confidence bounds and the grey circles are the residuals.

4.2 Assignments and validation of the three diffusion states of Zur.

After sorting the individual cells into groups of similar range of cellular protein concentrations, we obtained the CDF(r) for each group and performed global CDF analysis across the cellular protein concentrations, in which the diffusion constants of respective diffusion states were shared while the factional populations were varied, since the effective diffusion constants are expected to be concentration independent. This global analysis of CDF was applied to all strains and conditions. Three terms in CDF(r) were always the minimal number of diffusion states to satisfactorily fit the CDF. Exemplary CDF fits over all cellular protein concentration ranges are shown in Figure S6, and the fitted D's and A's are summarized in Table S5 and Table S6.

The fastest diffusing state was assign as the FD state (i.e., freely diffusing Zur proteins in the cytosol, D_{FD}), the medium diffusing state as the NB state (i.e., nonspecifically bound to DNA, D_{NB}), and the slowest one as TB (i.e., tightly bound at a chromosomal site, either a consensus recognition site or a tight-binding site that does not have a consensus sequence, D_{TB}) whose motions reflect mostly the chromosome dynamics and localization uncertainties(15-17,20,26,27). Then Equation S[10] became:

$$\mathrm{CDF}(r) = A_{\mathrm{FD}}\left(1-\exp\left(-\frac{r^2}{4D_{\mathrm{FD}}T_{\mathrm{tl}}}\right)\right) + A_{\mathrm{NB}}\left(1-\exp\left(-\frac{r^2}{4D_{\mathrm{NB}}T_{\mathrm{tl}}}\right)\right) + (1 - A_{\mathrm{FD}} - A_{\mathrm{NB}})\left(1-\exp\left(-\frac{r^2}{4D_{\mathrm{TB}}T_{\mathrm{tl}}}\right)\right) \qquad \text{S[11]}$$

Note that, we assigned the slowest diffusion state as 'tight binding' instead of the term 'specific binding' used in a previous study(4), since the interaction of non-repressor form of Zur to the Zur box consensus sequence DNA is known to be much weaker than the repressor form of Zur and therefore we postulated that the tight-binding of $\mathrm{Zur}_{\mathrm{C88S}}$ to chromosome in the cell should be at a non-consensus DNA site. Moreover, we couldn't rule out the possibility that repressor form of Zur could also bind at these non-consensus sequence sites. Therefore, for repressor form of Zur, tight binding sites may include both consensus and non-consensus sites, and for $\mathrm{Zur}_{\mathrm{C88S}}$ tight binding sites only refer to the non-consensus sites.

The extracted effective diffusion constants, $D_{\mathrm{FD}} = 5.0 \pm 0.5$, $D_{\mathrm{NB}} = 0.82 \pm 0.05$, and $D_{\mathrm{TB}} = 0.040 \pm 0.003$ from the CDF analysis were closed to the reported values ($D_{\mathrm{FD}} = 3.7 \pm 0.2$, $D_{\mathrm{NB}} = 0.70 \pm 0.03$, and $D_{\mathrm{SB}} = 0.036 \pm 0.009$) of CueR and ZntR, two metalloregulators in *E. coli*(4) (all units are $\mu m^2/s$). The major difference in FD state stemmed from the time-lapse difference, which more significantly influences the effective diffusion constant of faster moving ones (here T_{tl} = 40 ms, but T_{tl} = 60 ms for the work on CueR and ZntR(4)). Note that the effective diffusion constant of free mEos3.2 is 11.4 ± 0.3 $\mu m^2\ s^{-1}$ at T_{tl} = 15 ms. Also, due to the crowded environment in the cytosol, the time-lapse dependence of D follows approximately a power law fashion (that is, $D = D_\alpha T_{\mathrm{tl}}^{\alpha}$)(4,28). These previous observations could provide the expected range of D_{FD}, ($4.5 \le D_{\mathrm{FD}} \le 6.5$), which directly validate our FD state assignment.

Note these diffusion constant values are not the intrinsic ones, as they are influenced by the cell confinement effect(29), which decreases the magnitude of the apparent diffusion constant, and by the time-lapse effect of imaging, where longer time lapse gives apparently smaller diffusion constants; both of these effects are most significant on the FD state, less on the NB state, and negligible on the TB state, and were evaluated quantitatively in a previous study of metal-responsive transcription regulators of a different family(4).

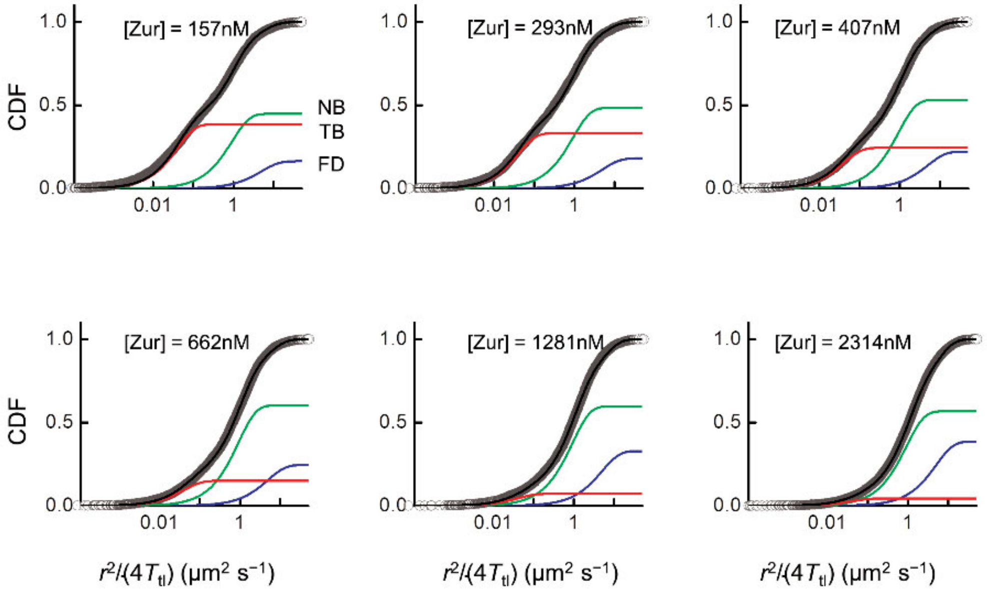

Figure S6. Exemplary global analysis of protein-concentration-sorted CDF of displacement length r (plotted against $r^2/(4T_{tl})$) for Zur^{mE}. The grey circles are the experimental CDF data; the black curve is the overall fit of three diffusion states of CDF(r), which are individually plotted as blue, green, and red curves for FD, NB, and TB states, respectively. The mean values of total cellular protein concentration [Zur^{mE}] are given in the respective panels.

Table S5. Extracted effective diffusion constants of Zur variants in live *E. coli.* cells

	D_{FD} (μm²/s)	D_{NB} (μm²/s)	D_{TB} (μm²/s)
Zur^{mE}	4.93 ± 0.59	0.92 ± 0.07	0.0395 ± 0.0044
Zur^{mE}_{C88S}	5.01 ± 0.46	0.82 ± 0.05	0.0396 ± 0.0034
Zur^{mE}_{Zn}	6.65 ± 0.51	0.82 ± 0.05	0.0327 ± 0.0028
$Zur^{mE}_{C88S, D49A}$	5.47 ± 0.96	0.82 ± 0.06	0.047 ± 0.008
$Zur^{mE}_{Zn, D49A}$	5.83 ± 0.60	0.85 ± 0.05	0.044 ± 0.008

Table S6. Extracted fractional populations of Zur variants in live *E. coli.* cells

	$[P]_{cell}$ (nM)	A_{FD} (%)	A_{NB} (%)	A_{TB} (%)
Zur^{mE}	157 ± 45	17.6 ± 0.3	44.2 ± 0.4	38.2 ± 0.4
	293 ± 36	18.3 ± 0.4	48.8 ± 0.4	32.9 ± 0.5
	407 ± 33	25.6 ± 0.6	53.5 ± 0.9	20.9 ± 0.7
	664 ± 145	24.0 ± 0.5	62.5 ± 0.6	13.5 ± 0.3
	1281 ± 207	33.3 ± 0.6	58.6 ± 0.5	8.1 ± 0.3
	2317 ± 348	39.8 ± 0.4	55.9 ± 0.4	4.3 ± 0.2
	3590 ± 602	42.6 ± 0.5	53.6 ± 0.4	3.8 ± 0.1
Zur^{mE}_{C88S}	150 ± 45	18.0 ± 0.3	50.3 ± 0.5	31.7 ± 0.4
	295 ± 36	21.7 ± 0.4	48.2 ± 0.6	30.1 ± 0.5
	414 ± 33	19.6 ± 0.6	56.9 ± 0.4	23.5 ± 0.7
	683 ± 136	27.7 ± 0.6	57.6 ± 0.3	14.7 ± 0.4
	1255 ± 214	38.0 ± 0.4	52.5 ± 0.5	9.5 ± 0.4
	2229 ± 338	41.5 ± 0.3	53.9 ± 0.4	4.6 ± 0.3
	3783 ± 683	43.4 ± 0.2	50.6 ± 0.3	5.9 ± 0.1
Zur^{mE}_{Zn}	148 ± 50	12.8 ± 0.2	49.6 ± 0.2	37.6 ± 0.4
	290 ± 33	18.1 ± 0.3	47.2 ± 0.6	34.7 ± 0.6
	414 ± 36	22.8 ± 0.3	42.9 ± 0.4	34.3 ± 0.5
	705 ± 140	36.1 ± 0.4	46.1 ± 0.4	17.8 ± 0.4
	1257 ± 205	39.8 ± 0.4	50.9 ± 0.3	9.3 ± 0.2
	2248 ± 336	44.3 ± 0.2	48.6 ± 0.2	7.1 ± 0.1
	3621 ± 531	42.7 ± 0.5	51.1 ± 0.4	6.2 ± 0.2
$Zur^{mE}_{C88S, D49A}$	164 ± 40	15.1 ± 0.3	54.6 ± 0.6	30.3 ± 0.6
	283 ± 33	19.5 ± 0.5	55.8 ± 0.6	24.7 ± 0.6
	417 ± 36	17.0 ± 0.8	63.5 ± 0.8	19.5 ± 0.3
	702 ± 133	25.2 ± 0.6	66.3 ± 0.6	8.5 ± 0.2
	1250 ± 205	29.4 ± 0.7	63.8 ± 0.5	6.8 ± 0.3
	2171 ± 360	35.9 ± 0.6	58.7 ± 0.5	5.4 ± 0.3
	3145 ± 207	31.6 ± 0.9	63.4 ± 1.2	5.0 ± 0.5
$Zur^{mE}_{Zn, D49A}$	169 ± 38	13.3 ± 0.4	54.2 ± 0.5	32.5 ± 0.3
	286 ± 36	20.5 ± 0.4	52.3 ± 0.5	27.2 ± 0.5
	410 ± 36	20.9 ± 0.5	58.0 ± 0.6	21.1 ± 0.8

	719 ± 133<br>1279 ± 210<br>2214 ± 352<br>3448 ± 457	24.6 ± 0.4<br>27.3 ± 0.4<br>31.8 ± 0.3<br>32.5 ± 0.2	57.0 ± 0.5<br>61.8 ± 0.2<br>61.2 ± 0.2<br>63.4 ± 0.2	18.4 ± 0.5<br>10.9 ± 0.3<br>7.0 ± 0.2<br>4.1 ± 0.1

4.3 Protein concentration dependence of fractional populations (A_{FD}, A_{NB}, and A_{TB}).

The resolved CDF also provided fractional populations (that is A_{FD}, A_{NB}, and A_{TB}) of corresponding diffusion states, as well as their absolute concentrations. Exemplary protein-concentration-dependent fractional populations and absolute concentrations are shown in Figure 1D and Figure S7. Complete results are listed in Table S6.

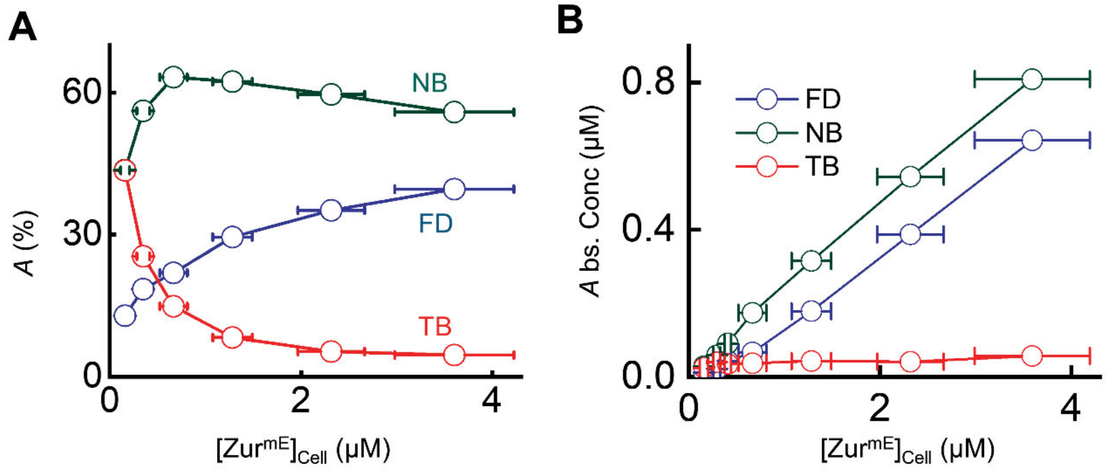

Figure S7. Exemplary protein-concentration-dependence of the fractional populations (A) and absolute concentrations (B) of Zur^{mE} strain diffusion states. The FD, NB, and TB states are plotted as blue, green, and red symbols, respectively. Error bars of protein concentrations are s.d.

With increasing cellular protein concentration, the fractional population of FD state, A_{FD}, increases while that of TB, A_{TB} decreases. These trends could further validate their assignments: at high protein concentrations, each protein molecule will spend more time freely diffusing than tightly bound to TB sites on chromosome since more protein molecules compete for the limited number of TB sites. On the other hand, the fraction of TB state out of the total protein should decrease with increasing concentration, again because there is limited number of TB sites on DNA. At the very high cellular protein concentrations, A_{TB} becomes negligible (< 5%), where we later extracted k_{NB}, the unbinding rate constant from NB sites (see Section 5).

5. Determination of apparent unbinding rate constant k_{-1} from TB site

The apparent unbinding rate constant k_{-1} of Zur was determined by analyzing the distributions of the microscopic residence time τ at TB sites, extracted from single-molecule displacement-vs-time trajectories, as we (4,30) have reported. When Zur is bound to a site on the chromosome tightly, its motion is stalled, which is reflected by the corresponding small displacement length r. Therefore, we could obtain the microscopic residence time τ by thresholding the displacement-vs-time trajectory with an upper limit r_o (=200 nm), which was chosen to include >99.5% of the TB state, as reflected in the PDF(r; t) (Figure 1B);

a complete τ should start upon transition from a large r to below r_o, and terminate upon transitions to above r_o.

Experimentally, the extracted residence time τ by r_o-thresholding could also be terminated by photobleaching/blinking of the mE tag. The photobleaching/blinking rate constant k_{bl} was independently determined from analyzing the distribution of the fluorescence on-times in the tracking trajectories (Figure S8A). Since we used stroboscopic imaging in which the 561 nm excitation laser was only illuminating during the integration time T_{int} (4 ms), and remained dark for the rest time within each time-lapse $\underline{T}_{tl}$ (40 ms), the apparent decay constant from the fluorescence on-time distribution needed to be corrected by the ratio of T_{int} and $\underline{T}_{tl}$. Therefore, the distribution of on-time was fitted with the equation below.

$$f_{on}(t) = N\exp\left(-k_{bl}\frac{T_{int}}{T_{tl}}t\right) \quad \text{S[12]}$$

N is a normalization constant. An exemplary fluorescence intensity trajectory and the histogram of the fluorescence on-times are shown in Figure S8A and B. The extracted k_{bl} is 263.2 ± 11.5 s^{-1}, consistent with the reported value (257 ± 9 s^{-1}) under similar 561 nm excitation conditions (4).

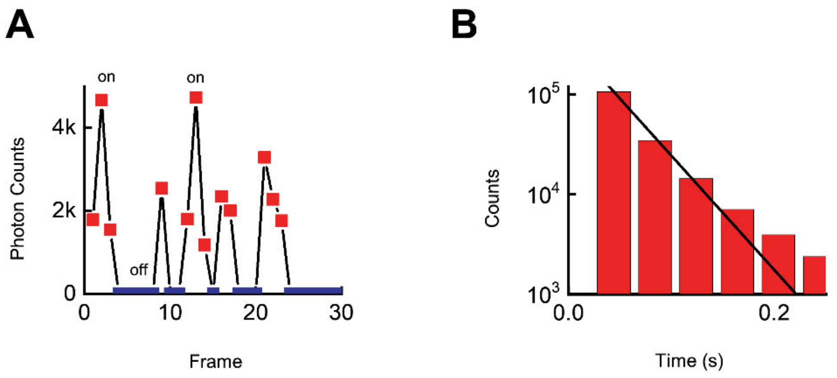

Figure S8. Determination of photobleaching/blinking rate constant k_{bl}. (A) An example of single-molecule mE fluorescence vs. time trajectory of $\mathrm{Zur}^{\mathrm{mE}}_{\mathrm{C88S}}$ in one imaging cycle. Red/blue square represents on/off time. The on time frames are first identified by their pixel counts to be above the threshold which is the mean value plus four standard deviation of the pixel counts of the whole image; then the identified on frames were analyzed by the PSF fitting procedure to get the integrated fluorescence intensity of each fitted PSF, as described in Section 3. One frame = 40 ms. (B) The distribution of fluorescence on-time for $\mathrm{Zur}^{\mathrm{mE}}_{\mathrm{C88S}}$ (red bar) and the fitting with Equation S[12] (black curve) to obtain k_{bl}.

Diffusion process is probabilistic at the microscopic level, however. Even proteins freely diffusing (FD) or non-specifically bound (NB) to chromosome, which have relatively large effective diffusion constant (5.0 $\mu m^2/s$ and 0.82 $\mu m^2/s$, respectively, for $\mathrm{Zur}^{\mathrm{mE}}_{\mathrm{C88S}}$), have finite probabilities to have small displacement length (e.g., Figure 1B), and thus will contribute to the distribution of residence time τ thresholded by r_o. The proportions of FD and NB states below r_o (= 200 nm) are 4.9%, and 26.3%, respectively (e.g., PDF(r) in Figure 1B), as compared with that >99.5% of the TB state. To deconvolute the contributions from FD and NB states to the residence time distribution, we first calculated the survival probability $S(r_o, t)$, which is the probability for a protein that was at the origin to survive within a circle of radius r_o within time t, as we previously derived(4). This probability is a product of multiple terms, each representing an independent process that affects this survival, including diffusion, dissociation of protein from chromosome, and photobleaching/blinking of mE (Equation S[13]).

$$S(r_o,t)=\left[1\text{-exp}\left(-\frac{r_o^2}{4Dt}\right)\right]\exp(-k_{\text{eff}}t) \qquad \text{S[13]}$$

Here, the term in [ ] corresponds to the survival probability due to diffusion, and k_{eff} is the sum of the unbinding rate constant (applicable for the TB and the NB state only) and the effective photobleaching/blinking rate constant (i.e., $k_{\text{bl}}\frac{T_{\text{int}}}{T_{\text{tl}}}$). The overall survival probability within r_0 for a Zur protein is a linear combination of survival probabilities of each state weighted by its fractional population.

$$S_{\text{all}}(r_o,t)=A_{\text{FD}}S_{\text{FD}}(r_o,t)+A_{\text{NB}}S_{\text{NB}}(r_o,t)+A_{\text{TB}}S_{\text{TB}}(r_o,t) \qquad \text{S[14]}$$

Then, the respective probability distribution function of the thresholded residence time τ, for the FD, NB, and TB states (that is, $\varphi_{\text{FD}}(\tau)$, $\varphi_{\text{NB}}(\tau)$, and $\varphi_{\text{TB}}(\tau)$) can be obtained by taking a time-derivative of the survival probability (i.e., $\varphi(\tau)=-\frac{\partial S(t)}{\partial t}|_{t=\tau}$):

$$\varphi_{\text{all}}(\tau) = A_{\text{FD}}\varphi_{\text{FD}}(\tau)+A_{\text{NB}}\varphi_{\text{NB}}(\tau)+A_{\text{TB}}\varphi_{\text{TB}}(\tau) \qquad \text{S[15]}$$

$$\varphi_{\text{FD}}(\tau)=\left[\frac{r_o^2}{4D_{\text{FD}}\tau^2}\exp\left(-\frac{r_o^2}{4D_{\text{FD}}\tau}\right)+k_{\text{eff}}^{\text{FD}}\left(1\text{-exp}\left(-\frac{r_o^2}{4D_{\text{FD}}\tau}\right)\right)\right]\exp\left(-k_{\text{eff}}^{\text{FD}}\tau\right) \qquad \text{S[16]}$$

$$\varphi_{\text{NB}}(\tau)=\left[\frac{r_o^2}{4D_{\text{NB}}\tau^2}\exp\left(-\frac{r_o^2}{4D_{\text{NB}}\tau}\right)+k_{\text{eff}}^{\text{NB}}\left(1\text{-exp}\left(-\frac{r_o^2}{4D_{\text{NB}}\tau}\right)\right)\right]\exp\left(-k_{\text{eff}}^{\text{NB}}\tau\right) \qquad \text{S[17]}$$

$$\varphi_{\text{TB}}(\tau)=k_{\text{eff}}^{\text{TB}}\exp\left(-k_{\text{eff}}^{\text{TB}}\tau\right) \qquad \text{S[18]}$$

Here, $k_{\text{eff}}^{\text{FD}} = k_{\text{bl}}\frac{T_{\text{int}}}{T_{\text{tl}}}$, $k_{\text{eff}}^{\text{ND}} = k_{\text{bl}}\frac{T_{\text{int}}}{T_{\text{tl}}} + k_{-2}$ and $k_{\text{eff}}^{\text{TB}} = k_{\text{bl}}\frac{T_{\text{int}}}{T_{\text{tl}}} + k_{-1}$ (see Figure 2C for definition of rate constants). Note, since r_o (=200 nm) was chosen to include >99.5% displacement length of the TB state, the time-derivative of the diffusion-term in $S_{\text{TB}}(r_o,t)$ became negligible. Therefore, only the exponential term survived for the TB state (that is, $S_{\text{TB}}(r_o,t) = \exp\left(-k_{\text{eff}}^{\text{TB}}t\right)$). k_{-2} is the unbinding rate constant from the NB sites. k_{-2} was extracted from the highest cellular concentration regime by fitting the residence time distribution with Equation S[19], which is a reduced version of Equation S15 in which A_{TB} is <5% and $A_{\text{TB}}\varphi_{\text{TB}}(\tau)$ term becomes negligible:

$$\varphi_{all}(\tau) = A_{\text{FD}}\varphi_{\text{FD}}(\tau)+A_{\text{NB}}\varphi_{\text{NB}}(\tau) \qquad \text{S[19]}$$

k_{-1} was extracted by fitting the residence time distribution with Equation S[15] with predetermined D's, A's, k_{bl}, and k_{-2}. All determined rate constants are summarized in Table S7. This method of extracting k_{-1} from residence time distribution was rigorously verified by using simulation data, in our previous study(4).

We further obtained the simple average of the residence time $\langle\tau\rangle$, and the single exponential fit of the residence time distribution (i.e., fitting the distribution with $y = \exp\left[-\left(\frac{1}{\tau_{\text{PP}}}+k_{\text{bl}}\frac{T_{\text{int}}}{T_{\text{tl}}}\right)t\right]$, which includes the time constant τ_{PP}, for which $1/\tau_{\text{pp}}$ approximates the unbinding rate constant, and the correction for photobleaching/blinking process) for each group of cells with similar cellular protein concentrations. Since $\langle\tau\rangle$ and τ_{PP} are inversely related to the apparent unbinding rate constant k_{-1}, the dependence of $\langle\tau\rangle$ and τ_{PP} on cellular protein concentration might have a biphasic behavior (increasing-followed-by-decreasing) for $\text{Zur}_{\text{C88S}}^{\text{mE}}$ and Zur^{mE} with increasing cellular protein concentrations, or a monotonous decreasing behavior for $\text{Zur}_{\text{Zn}}^{\text{mE}}$. Figure S9 clearly showed the expected behaviors, supporting the robustness of the biphasic

unbinding behavior of Zur from DNA (or the facilitated unbinding of repressor form of Zur within the accessible protein concentration range).

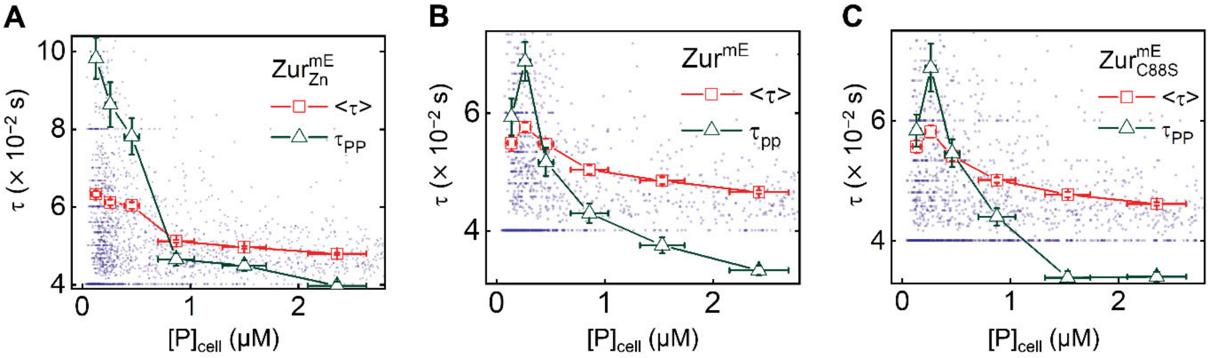

Figure S9. Correlation of $\langle\tau\rangle$ with average protein concentration in each cell for Zur^{mE}_{Zn} (A), Zur^{mE} (B) and Zur^{mE}_{C88S} (C) (each blue dot = 1 cell) from a total of ~1300 cells each. The individual cells are then grouped by similar protein concentration ranges and averaged within each group to obtain the averages (open red squares connected by red lines). Further correction of photobleaching/blinking kinetics of the mE tag gives the corresponding constant τ_{PP} from a single exponential fit (open green triangles connected by green lines). *x, y* error bars are s.d. and s.e.m.

6. Mechanistic model of the biphasic concentration dependence of k_{-1}: impeded followed by facilitated unbinding

Using single-molecule tracking, our previous study showed that the apparent first-order unbinding rate constants of the metalloregulator CueR and ZntR show linear dependences on their cellular free protein concentration (i.e., facilitated unbinding), highly analogous to the facilitated unbinding phase of Zur observed here. For CueR/ZntR, we proposed that the facilitated unbinding results from the formation of a ternary complex intermediate, in which two protein dimers are bound to the recognition site, each of which uses one DNA-binding domain attaching to half of the dyad recognition sequence, leading to assisted dissociation and direct substitution eventually(4,31) (Figure S10). Similar facilitated unbinding (i.e., concentration-enhanced) was also observed for the nonspecific dsDNA binding protein nucleoid associate proteins, ssDNA binding protein replication protein A, and DNA polymerases(32-35), and the same mechanism involving a ternary complex was invoked, reaching a mechanistic consensus as we recently reviewed (36). This ternary complex was also kinetically resolved for CueR interacting with an engineered DNA structure in a previous study of ours(37). We think the facilitated unbinding phase of Zur observed here also follows the same mechanism (Figure S10), for which the effective first-order unbinding rate constant can be written as $k_f[P]_{FD}$, where k_f is a 2nd-order kinetic rate constant and $[P]_{FD}$ is the freely diffusing Zur concentration in the cell.

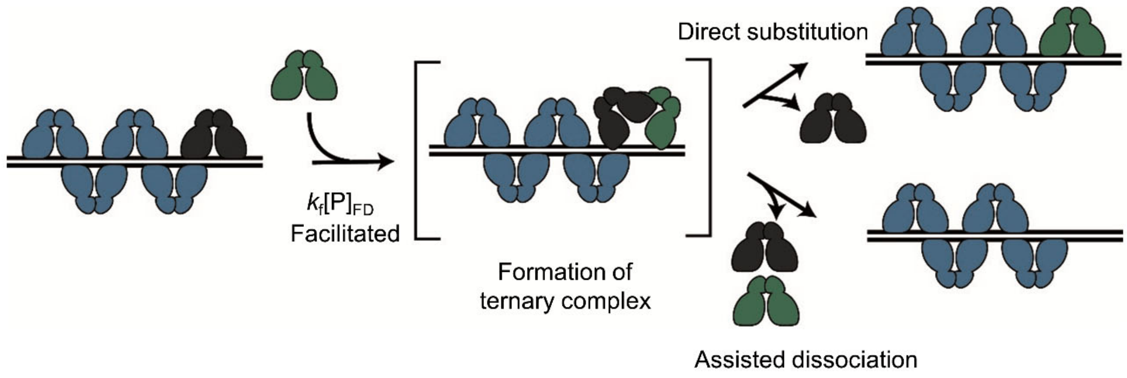

Figure S10. Proposed mechanism for facilitated unbinding involving a ternary complex as an intermediate, in which its formation rate is linearly proportional to free-protein concentration in the cell. Then it proceeds to direct substitution or assisted dissociation pathway, similarly as we(4,31) and others(32,33) described previously. The scheme shown here is replicated in Figure 2E, lower, in the main text.

6.1 Cooperativity among oligomerized proteins can explain the impeded unbinding

In this section, we formulate a quantitative mechanistic model to account for the impeded unbinding behavior of Zur. The crystal structure of *E. coli* Zur (in metallated repressor form) on a short 31 bp DNA oligo was solved(1), which showed that two Zur dimers can bind simultaneously to DNA, in which a pair of salt bridges were identified between the two dimers. Later, footprint assay showed that Zur from *Streptomyces coelicolor* can oligomerize on DNA, in which the oligomerization number is likely significantly greater than two(38) (note that the oligomerization number of the crystal structure of *E. coli* Zur on DNA is 2, i.e., two dimers). Therefore, it is reasonable to postulate that Zur in *E. coli* also has strong oligomerization tendency, i.e., the oligomerization number n_o can be greater than 2, considering that there is ample space on the chromosome. Here we propose that unbinding rate of Zur is attenuated due to the intermolecular interactions between the dimers that oligomerize at a TB site, as shown schematically in Figure S11.

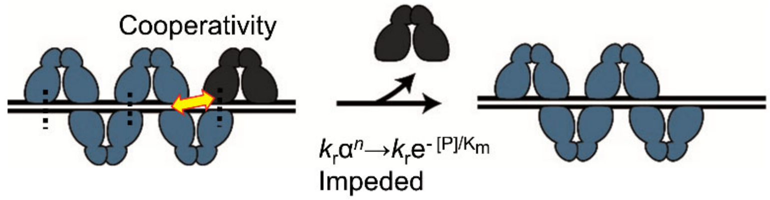

Figure S11. Proposed mechanism for impeded unbinding involving oligomerization at a TB site, where the oligomerized proteins have a positive cooperativity via salt-bridge interactions(black dash lines) for stabilization to make unbinding of a protein slow down. The unbinding rate constant is attenuated exponentially by the number of proteins in the oligomer (Equation S[21]), leading to an exponential decay dependence on the free-protein concentration in the cell (Equation S[21]). The scheme shown here is replicated in Figure 2E, upper, in the main text.

Based on the proposed mechanism in Figure S11, the microscopic kinetic scheme of protein binding-unbinding at any single TB site can be written as:

$$P_{n-1}D + P \underset{k_{-1}^{(n)}}{\overset{k_1}{\rightleftarrows}} P_nD \qquad \text{S[20]}$$

where, the binding of a dimeric protein P to a TB site that already has n-1 proteins bound on DNA occur with a rate constant k_1, and the corresponding unbinding rate constant is $k_{-1}^{(n)}$, which depends on n, the number of proteins bound at the TB site, which can vary from 1 to the oligomerization number n_0.

To account for the impeded unbinding kinetics, we model that a component of microscopic unbinding rate constant $k_{-1}^{(n)}$ is attenuated by a factor of α^n, where $0 < \alpha < 1$. Along with the facilitated unbinding component $k_f[P]_{FD}$ and an intrinsic unbinding component k_0, the overall microscopic unbinding rate constant at a TB site can be written as.

$$k_{-1}^{(n)} = k_o + k_r\alpha^n + k_f[P]_{FD} \qquad \text{S[21]}$$

If we assume that the (n-1)'th binding event does not affect n'th binding event (which is consistent with our model that the binding rate constant k_1 is independent of n) and the dependence of the unbinding of n'th protein on the prior n-1 proteins is a small perturbation, the distribution of n will follow Poisson distribution $\Phi(n) = \frac{\langle n\rangle^n}{n!}\exp[-\langle n\rangle]$, where $\langle n\rangle$ is the mean value of n.

Then the observable unbinding rate constant k_{-1} is given by

$$k_{-1} = \sum_{n=0}^{n_0} \Phi(n) k_{-1}^{(n)} = k_o + k_r e^{-\langle n\rangle} \sum_{n=0}^{n_0} \frac{(\alpha\langle n\rangle)^n}{n!} + k_f[P]_{FD} \qquad \text{S[22]}$$

$$\approx k_o + k_r e^{-(1-\alpha)\langle n\rangle} + k_f[P]_{FD}$$

Note that if n_0 is very large or $\alpha\langle n\rangle$ is very small, $\sum_{n=0}^{n_0} \frac{(\alpha\langle n\rangle)^n}{n!}$ in Equation S[22] reduces to $e^{\alpha\langle n\rangle}$, since $e^x = \sum_{n=0}^{\infty} \frac{x^n}{n!}$.

Since $\langle n\rangle$ is not a directly measurable quantity in our experiment, we need a relation of $\langle n\rangle$ with experimental observables, e.g., protein concentration. Intuitively, $\langle n\rangle$ should become larger when the free protein concentration $[P]_{FD}$ increases since in the absence of protein, $\langle n\rangle$ should be 0, and at very high concentration, $\langle n\rangle$ should approach n_o. And we found that at the low protein concentration limit, $\langle n\rangle$ is linearly proportional to $[P]_{FD}$, which is shown below.

The derivation of linear proportionality between $\langle n\rangle$ and $[P]_{FD}$ started from the relative population analysis between FD and TB states, which will be described in Section 7 below. In Equation S[34], $\frac{[PD]_{TB}}{[P]_{FD}} = \frac{k_1[D_0]_{TB}}{k_{-1}} \frac{\partial \ln F_{TB\leftarrow FD}(x_{TB\leftarrow FD})}{\partial x_{TB\leftarrow FD}}$, here $[PD]_{TB}$, $[P]_{FD}$, and $[D_0]_{TB}$ are the cellular protein concentrations of TB and FD states, and the cellular concentration of TB sites, respectively, and $F_{TB\leftarrow FD}(x_{TB\leftarrow FD}) \equiv \sum_{i=0}^{n_0} x_{TB\leftarrow FD}^i$,

where $x_{\mathrm{TB}\leftarrow\mathrm{FD}} \equiv \frac{k_1}{k_{-1}}[\mathrm{P}]_{\mathrm{FD}}$. We used a low-concentration-limit approximation where $F_{\mathrm{TB}\leftarrow\mathrm{FD}}\left(x_{\mathrm{TB}\leftarrow\mathrm{FD}}\right) \overset{[\mathrm{P}]_{\mathrm{FD}}\to 0}{=} 1 + x_{\mathrm{TB}\leftarrow\mathrm{FD}} = 1 + \frac{k_1[\mathrm{P}]_{\mathrm{FD}}}{k_{\mathrm{o}}^{\mathrm{off}}}$ (note $k_{\mathrm{o}}^{\mathrm{off}}$, defined as $k_{\mathrm{o}}^{\mathrm{off}} \equiv k_{\mathrm{o}} + k_{\mathrm{r}}$, is the limiting value of k_{-1} when $[\mathrm{P}]_{\mathrm{FD}}$ approaches zero and is also equivalent to the y-intercept of k_{-1} -vs-$[\mathrm{P}]_{\mathrm{FD}}$ shown in Figure 2D; $k_{\mathrm{o}}^{\mathrm{off}}$ replaces k_{-1} here under the approximation of low concentration; see also Equation S[25] later). Then we have:

$$\frac{[PD]_{\mathrm{TB}}}{[P]_{\mathrm{FD}}} = \frac{k_1[D_0]_{\mathrm{TB}}}{k_{-1}} \frac{\partial \ln F_{\mathrm{TB}\leftarrow\mathrm{FD}}(x_{\mathrm{TB}\leftarrow\mathrm{FD}})}{\partial x_{\mathrm{TB}\leftarrow\mathrm{FD}}} \overset{[\mathrm{P}]_{\mathrm{FD}}\to 0}{=} \frac{k_1[D_0]_{\mathrm{TB}}}{k_{\mathrm{o}}^{\mathrm{off}}\left(1+\frac{k_1[P]_{\mathrm{FD}}}{k_{\mathrm{o}}^{\mathrm{off}}}\right)} \overset{[\mathrm{P}]_{\mathrm{FD}}\to 0}{=} \frac{k_1[D_0]_{\mathrm{TB}}}{k_{\mathrm{o}}^{\mathrm{off}}} \qquad \text{S[23]}$$

By definition, $\langle n\rangle = \frac{[\mathrm{PD}]_{\mathrm{TB}}}{[\mathrm{D}_0]_{\mathrm{TB}}}$, therefore by substituting Equation S[23] into $\langle n\rangle$, we can obtain the relation between $\langle n\rangle$ and $[\mathrm{P}]_{\mathrm{FD}}$.

$$\langle n\rangle = \frac{[\mathrm{PD}]_{\mathrm{TB}}}{[\mathrm{D}_0]_{\mathrm{TB}}} \overset{[\mathrm{P}]_{\mathrm{FD}}\to 0}{=} \frac{k_1}{k_{\mathrm{o}}^{\mathrm{off}}}[\mathrm{P}]_{\mathrm{FD}} \qquad \text{S[24]}$$

Equation S[24] predicts a linear relation between $[\mathrm{PD}]_{\mathrm{TB}}$ and $[\mathrm{P}]_{\mathrm{FD}}$ at the low protein concentration regime. This linear relation can be verified experimentally, as shown in Figure S12.

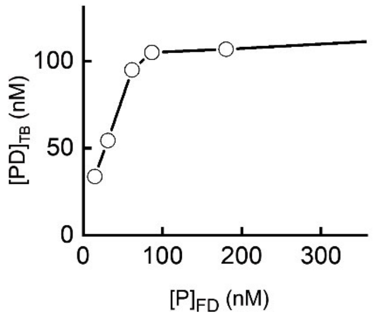

Figure S12. Dependence of $[\mathrm{PD}]_{\mathrm{TB}}$ for $\mathrm{Zur}^{\mathrm{mE}}_{\mathrm{C88S}}$ on $[\mathrm{P}]_{\mathrm{FD}}$. Linear dependence at the low free concentration regime (up to ~100 nM) was observed, which verifies Equation S[24].

Then we plug in Equation S[24] into Equation S[22], which leads to:

$$k_{-1} = k_{\mathrm{o}}^{\mathrm{off}} + k_{\mathrm{r}}\left(e^{-\frac{[\mathrm{P}]_{\mathrm{FD}}}{K_{\mathrm{m}}}} - 1\right) + k_{\mathrm{f}}[\mathrm{P}]_{\mathrm{FD}} \qquad \text{S[25]}$$

where $K_{\mathrm{m}} \equiv \frac{k_0^{\mathrm{off}}}{k_1(1-\alpha)}$, and $k_{\mathrm{o}}^{\mathrm{off}} \equiv k_{\mathrm{o}} + k_{\mathrm{r}}$, which represents a protein-concentration-independent unbinding rate constant. Equation S[25] gives the overall free protein concentration dependence of k_{-1}. By fitting the experimentally measured unbinding rate constant, we can obtain $k_{\mathrm{o}}^{\mathrm{off}}$, k_{r}, k_{f}, and K_{m}. Note that K_{m}

has the concentration unit, and thus it represents the effective affinity of oligomerization (that is, the lower the K_m, the stronger the oligomerization tendency). This equation shows that k_{-1} approaches k_o^{off} when $[P]_{FD} \to 0$, which was used to derive Equation S[23]. We also obtained $[P]_{FD}^{min}$, which is the free-protein concentration at which k_{-1} reaches the minimum by $\frac{\partial k_{-1}}{\partial [P]_{FD}}|_{[P]_{FD}=[P]_{FD}^{min}}=0$.

$$[P]_{FD}^{min} = K_m \ln\left(\frac{k_r}{k_f K_m}\right) \qquad \text{S[26]}$$

All fitted parameters are listed in Table S7.

6.2 Overall mechanistic model of Zur-DNA interactions in cells

Based on the evidence of oligomerization of Zur on TB sites and three diffusion states comprising FD, NB, and TB states, we formulated a minimal mechanistic model for the overall Zur-DNA interaction in the cell (Figure 2C and Figure S13 below).

Among the three states, this model has interconverting kinetic processes. Here, k_1 is a rate constant from FD to TB state, in which the apparent binding rate is linearly scaled with the effective concentration of vacant regulator TB sites $[D]_{TB}$ in the cell (that is, $k_1[D]_{TB}$). The apparent unbinding rate constant k_{-1} includes three terms: the spontaneous, impeded, and facilitated unbinding as described in the previous section. The interconverting rate constants between FD and NB via binding nonspecifically to DNA are k_2 and k_{-2}, in which the apparent rate for binding is also linearly scaled with the effective concentration of vacant non-specific DNA binding sites $[D]_{NB}$ in the cell (that is, $k_2[D]_{NB}$). k_3 and k_{-3} are the interconversion rate constants between TB and NB. They are assumed to be very small because the number of TB sites is very small relative to that of NB sites, which means when a regulator is at a NB site, TB sites are far away making the direct transition less probable compared with unbinding from DNA. All the kinetic processes can be terminated by the photobleaching/blinking process of mEos3.2 tag with the rate constant k_{bl}.

7. Additional kinetic and thermodynamic parameters from the analysis of relative populations of FD, NB, and TB states

7.1 Analysis of relative populations of the FD, NB, and TB states using quasi-equilibrium model

In this section, we will introduce how to extract additional kinetic and thermodynamic quantities by analyzing the relative populations of the FD, NB, and TB states resolved by the analysis of the displacement distribution from single-molecule tracking of mE-tagged Zur in the cell. The fastest diffusing state is assign as FD (i.e., freely diffusing Zur proteins in the cytosol), the medium diffusing state as NB (i.e., nonspecifically bound to DNA), and the slowest one as TB (i.e., tightly bound at a chromosomal site, either a consensus recognition site or any other tight-binding site that does not have a consensus sequence) in the cell.

Both kinetic and thermodynamic analyses are based on the kinetic model in Figure S13, which includes the three states with the corresponding interconversion rate constants. Note that Zur protein can oligomerize at any TB site, with the number of Zur homodimers at any time at a site being n, which cannot be greater than n_o, the oligomerization number.

We assume that since the protein can sample the FD, NB, and TB states rapidly in the cell, we can approximate a quasi-equilibrium of binding and unbinding of regulators on DNA. This approximation is valid when the time scale of interconversion between states (~ms, see Section 7.4 and Table S7) are much faster than the experimental imaging time (~30 min to 1 hour for each cell), during which the cellular Zur protein concentration does not vary much.

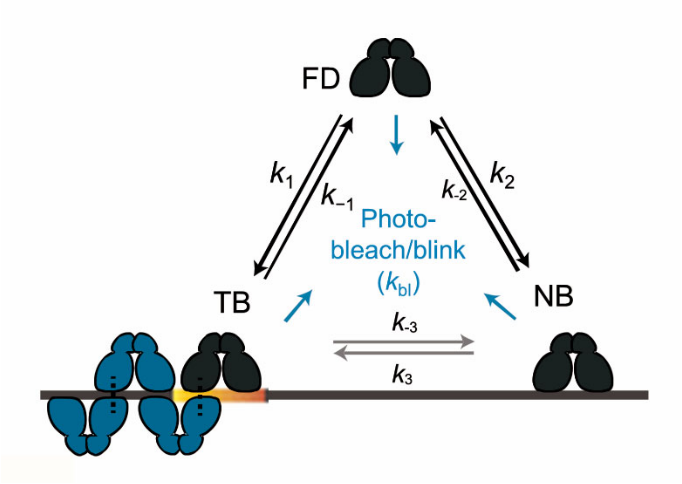

Figure S13. Overall mechanistic model of Zur-DNA interactions in a cell. Interconversion rate constants are denoted *k'*s. Figure here is the same as Figure 2C in the main text.

7.1.1 Determination of binding rate constant (i.e., k_1) and affinity (i.e., $K_{D1} \equiv k_o^{off}/k_1$) to tight binding sites from relative populations of TB and FD states, $[PD]_{TB}/[P]_{FD}$

In this section, we will derive the equation to extract the binding rate constant (k_1) and affinity (K_{D1}) to the tight binding sites from relative populations of FD and TB states, focusing on the quasi-equilibrium between the two states. The sequence of derivation is analogous (but with modifications) to that of Brunauer-Emmett-Teller (BET) (39) adsorption isotherm model for multilayer adsorption of molecules onto surface. Mechanistically, modeling of our protein-DNA interaction as a BET adsorption process is reasonable because the model explicitly describes sequential binding and unbinding of multiple proteins. The differences between the BET model and our model are that: 1) BET model assumes infinite number of layers for molecular adsorption, whereas our model has an upper limit being the oligomerization number (n_o) at each TB site. 2) BET model has two types of binding rate constant (to form the first layer and the rest), whereas our model has one binding rate constant. As the derivation of BET model did, one can define $\theta_0, \theta_1, \theta_2, \cdots, \theta_n, \cdots, \theta_{n_0}$, where n_o is the maximum number of Zur dimers at each TB site.

$$\theta_n = \frac{\text{The number of TB sites with } n \text{ dimers adsorbed}}{\text{Total number of TB sites}} \qquad \text{S[27]}$$

Note that the value of θ_n can vary from 0 to 1. At quasi-equilibrium, $\frac{d\theta_n}{dt} = 0$. With binding rate constant and unbinding rate constant, k_1 and k_{-1}, respectively, we can obtain the expression of θ_n as a function of k_1 and k_{-1}, which is shown below.

$$0 = \frac{d\theta_0}{dt} = k_{-1}\theta_1 - k_1\theta_0[\mathrm{P}]_{\mathrm{FD}}, \quad \theta_1 = \frac{k_1}{k_{-1}}[\mathrm{P}]_{\mathrm{FD}}\theta_0 \qquad \text{S[28]}$$

$$0 = \frac{d\theta_1}{dt} = -k_{-1}\theta_1 + k_1\theta_0[\mathrm{P}]_{\mathrm{FD}} + k_{-1}\theta_2 - k_1\theta_1[\mathrm{P}]_{\mathrm{FD}},$$

$$\theta_2 = \frac{k_1}{k_{-1}}[\mathrm{P}]_{\mathrm{FD}}\theta_1 = \left(\frac{k_1}{k_{-1}}[\mathrm{P}]_{\mathrm{FD}}\right)^2\theta_0 \qquad \text{S[29]}$$

$$\therefore\ \theta_n = \left(\frac{k_1}{k_{-1}}[\mathrm{P}]_{\mathrm{FD}}\right)^n\theta_0 = x^n\theta_0,\ \left(x_{\mathrm{TB}\leftarrow\mathrm{FD}} \equiv \frac{k_1}{k_{-1}}[\mathrm{P}]_{\mathrm{FD}}, 0 \le n \le n_0\right) \qquad \text{S[30]}$$

Here, the unbinding rate constant k_{-1} is a sum of the spontaneous unbinding rate constant, the impeded unbinding term, and the facilitated unbinding term, and it depends on protein concentration (i.e., $k_{-1} = \langle k_{-1}^{(n)} \rangle = k_{\mathrm{o}}^{\mathrm{off}} + k_{\mathrm{r}}\left(e^{-[\mathrm{P}]_{\mathrm{FD}}/K_{\mathrm{m}}} - 1\right) + k_{\mathrm{f}}[\mathrm{P}]_{\mathrm{FD}}$,from Equation S[25] in Section 6.1) and all other parameters are macroscopic quantities, which are discussed in Section 7.2.

Note that, this model uses k_{-1}, not $k_{-1}^{(n)}$, as an approximation. The microscopic unbinding rate constant $k_{\mathrm{d}}^{(n)}$ depends on n, but assuming its behavior is ergodic and once it samples ergodically all possible values of n over a period of time [note our experimental imaging time (~30 min to 1 h) is orders of magnitude longer than the timescale of protein binding/unbinding (~100 ms, see Section 7.4), it should converge to k_{-1}.

Then we can derive the average protein coverage at any TB site $\theta_{\mathrm{TB}\leftarrow\mathrm{FD}}$.

$$\theta_{\mathrm{TB}\leftarrow\mathrm{FD}} = \sum_{i=0}^{n_0} i\theta_i = \frac{\sum_{i=0}^{n_0} i\theta_i}{1} \overset{\sum_{i=0}^{n_0}\theta_i=1}{=} \frac{\sum_{i=0}^{n_0} i\theta_i}{\sum_{i=0}^{n_0}\theta_i} = \frac{\theta_0 \sum_{i=0}^{n_0} i x_{\mathrm{TB}\leftarrow\mathrm{FD}}^{i}}{\theta_0 \sum_{i=0}^{n_0} x_{\mathrm{TB}\leftarrow\mathrm{FD}}^{i}} = \frac{x_{\mathrm{TB}\leftarrow\mathrm{FD}} \sum_{i=1}^{n_0} i x_{\mathrm{TB}\leftarrow\mathrm{FD}}^{i-1}}{\sum_{i=0}^{n_0} x_{\mathrm{TB}\leftarrow\mathrm{FD}}^{i}}$$
$$= \frac{x_{\mathrm{TB}\leftarrow\mathrm{FD}}\left(1 + 2x_{\mathrm{TB}\leftarrow\mathrm{FD}} + 3x_{\mathrm{TB}\leftarrow\mathrm{FD}}^2 + \cdots + n_0 x_{\mathrm{TB}\leftarrow\mathrm{FD}}^{n_0-1}\right)}{\left(1 + x_{\mathrm{TB}\leftarrow\mathrm{FD}} + x_{\mathrm{TB}\leftarrow\mathrm{FD}}^2 + \cdots + x_{\mathrm{TB}\leftarrow\mathrm{FD}}^{n_0}\right)} \quad \text{S[31]}$$

$$\theta_{\mathrm{TB}\leftarrow\mathrm{FD}} = \frac{x_{\mathrm{TB}\leftarrow\mathrm{FD}} \frac{\partial F_{\mathrm{TB}\leftarrow\mathrm{FD}}\left(x_{\mathrm{TB}\leftarrow\mathrm{FD}}\right)}{\partial x_{\mathrm{TB}\leftarrow\mathrm{FD}}}}{F_{\mathrm{TB}\leftarrow\mathrm{FD}}\left(x_{\mathrm{TB}\leftarrow\mathrm{FD}}\right)} = x_{\mathrm{TB}\leftarrow\mathrm{FD}} \frac{\partial \ln F_{\mathrm{TB}\leftarrow\mathrm{FD}}\left(x_{\mathrm{TB}\leftarrow\mathrm{FD}}\right)}{\partial x_{\mathrm{TB}\leftarrow\mathrm{FD}}}, \text{ where} \quad \text{S[32]}$$
$$F_{\mathrm{TB}\leftarrow\mathrm{FD}}\left(x_{\mathrm{TB}\leftarrow\mathrm{FD}}\right) \equiv 1 + x_{\mathrm{TB}\leftarrow\mathrm{FD}} + x_{\mathrm{TB}\leftarrow\mathrm{FD}}^2 + \cdots + x_{\mathrm{TB}\leftarrow\mathrm{FD}}^{n_0} = \sum_{i=0}^{n_0} x_{\mathrm{TB}\leftarrow\mathrm{FD}}^{i}$$

The value of $\theta_{\mathrm{TB}\leftarrow\mathrm{FD}}$ can vary from 0 to n_0. Since the total number (or concentration) of proteins bound at all TB sites equals the total number (or concentration) of TB sites multiplied by the average protein coverage at any TB site (i.e., $\theta_{\mathrm{TB}\leftarrow\mathrm{FD}}$,), one can write

$$[\mathrm{PD}]_{\mathrm{TB}} = \theta[\mathrm{D}_0]_{\mathrm{TB}} = \frac{k_1[\mathrm{P}]_{\mathrm{FD}}[\mathrm{D}_0]_{\mathrm{TB}}}{k_{-1}} \frac{\partial \ln F_{\mathrm{TB}\leftarrow\mathrm{FD}}(x_{\mathrm{TB}\leftarrow\mathrm{FD}})}{\partial x_{\mathrm{TB}\leftarrow\mathrm{FD}}} \quad \text{S[33]}$$

$$\frac{[\mathrm{PD}]_{\mathrm{TB}}}{[\mathrm{P}]_{\mathrm{FD}}} = \frac{k_1[\mathrm{D}_0]_{\mathrm{TB}}}{k_{-1}} \frac{\partial \ln F_{\mathrm{TB}\leftarrow\mathrm{FD}}(x_{\mathrm{TB}\leftarrow\mathrm{FD}})}{\partial x_{\mathrm{TB}\leftarrow\mathrm{FD}}} \quad \text{S[34]}$$

Equation S[34] shows how the ratio of $[\mathrm{PD}]_{\mathrm{TB}}$ and $[\mathrm{P}]_{\mathrm{FD}}$ relate to k_1 and k_{-1}. By fitting $[\mathrm{PD}]_{\mathrm{TB}}/[\mathrm{P}]_{\mathrm{FD}}$ *vs* $[\mathrm{P}]_{\mathrm{FD}}$ with Equation S[34], we can extract k_1 and $K_{\mathrm{D1}}(\equiv k_{\mathrm{o}}^{\mathrm{off}}/k_1)$

7.1.2 Determination of K_{D3} (=k_{-3}/k_3) and $[D_0]_{TB}$ from $[PD]_{TB}/[PD]_{NB}$.

In this section, we will derive the connection between K_{D3} ($\equiv k_{-3}/k_3$), $[D_0]_{TB}$, and the relative populations of NB and TB states. The derivation of the equation for the ratio of $[PD]_{TB}$ and $[PD]_{NB}$ is analogous to that for the ratio of $[PD]_{TB}$ and $[P]_{FD}$, but the quasi-equilibrium under consideration is between the TB and NB states. The effective rate from TB to NB is $k_{-3}[D]_{NB}$, where $[D]_{NB}$ is the effective concentrations of vacant nonspecific DNA site. Since $[D]_{NB} = [D_0]_{NB}-[PD]_{NB}$ where $[D_0]_{NB}$ is the effective concentration of total nonspecific binding sites in the cell, we can obtain the average protein coverage at any TB site that is in equilibrium with the NB state, $\theta_{TB\leftarrow NB}$

$$\theta_{TB\leftarrow NB} = \sum_{i=0}^{n_0} i\theta_{i,TB\leftarrow NB} = \frac{\sum_{i=0}^{n_0} i\theta_{i,TB\leftarrow NB}}{1} \overset{\sum_{i=0}^{n_0}\theta_{i,TB\leftarrow NB}=1}{=} \frac{\sum_{i=0}^{n_0} i\theta_{i,TB\leftarrow NB}}{\sum_{i=0}^{n_0}\theta_{i,TB\leftarrow NB}} = \frac{\theta_{0,NB}\sum_{i=0}^{n_0} i x_{TB\leftarrow NB}^{i}}{\theta_{0,NB}\sum_{i=0}^{n_0} x_{TB\leftarrow NB}^{i}}$$

$$= \frac{x_{NB}\sum_{i=1}^{n_0} i x_{TB\leftarrow NB}{}^{i-1}}{\sum_{i=0}^{n_0} x_{TB\leftarrow NB}{}^{i}} = \frac{x_{TB\leftarrow NB}\left(1+2x_{TB\leftarrow NB}+3x_{TB\leftarrow NB}{}^{2}+\cdots+n_0 x_{TB\leftarrow NB}{}^{n_0-1}\right)}{\left(1+x_{TB\leftarrow NB}+x_{TB\leftarrow NB}{}^{2}+\cdots+x_{TB\leftarrow NB}{}^{n_0}\right)} \qquad \text{S[35]}$$

Here, $x_{TB\leftarrow NB} \equiv \frac{k_3}{k_{-3}\left([D_0]_{NB}-[PD]_{NB}\right)}[PD]_{NB}$, analogous to that in Equation S[30] earlier. Let $F_{TB\leftarrow NB}(x) \equiv 1+x_{TB\leftarrow NB}+x_{TB\leftarrow NB}{}^{2}+\cdots+x_{TB\leftarrow NB}{}^{n_0} = \sum_{i=0}^{n_0} x_{TB\leftarrow NB}{}^{i}$, then we have

$$\theta_{TB\leftarrow NB} = \frac{x_{TB\leftarrow NB}\frac{\partial F_{TB\leftarrow NB}(x_{TB\leftarrow NB})}{\partial x_{TB\leftarrow NB}}}{F_{TB\leftarrow NB}(x_{TB\leftarrow NB})} = x_{NB}\frac{\partial \ln F_{TB\leftarrow NB}(x_{TB\leftarrow NB})}{\partial x_{TB\leftarrow NB}} \qquad \text{S[36]}$$

Since the total number of proteins bound at all TB sites equals to the product of the total number of TB sites and the average protein coverage at any TB site (i.e., $\theta_{TB\leftarrow NB}$), we can write:

$$[PD]_{TB} = \theta_{TB\leftarrow NB}[D_0]_{TB} = \frac{k_3[PD]_{NB}[D_0]_{TB}}{k_{-3}\left([D_0]_{NB}-[PD]_{NB}\right)}\frac{\partial \ln F_{TB\leftarrow NB}(x_{TB\leftarrow NB})}{\partial x_{TB\leftarrow NB}} \qquad \text{S[37]}$$

$$\frac{[PD]_{TB}}{[PD]_{NB}} = \frac{k_3[D_0]_{TB}}{k_{-3}\left([D_0]_{NB}-[PD]_{NB}\right)}\frac{\partial \ln F_{TB\leftarrow NB}(x_{TB\leftarrow NB})}{\partial x_{TB\leftarrow NB}} \qquad \text{S[38]}$$

Equation S[38] shows how the ratio of $[PD]_{TB}$ and $[PD]_{NB}$ relates to K_{D3} ($\equiv k_{-3}/k_3$) and $[D_0]_{TB}$. By fitting $[PD]_{TB}/[PD]_{NB}$ *vs* $[PD]_{NB}$ with Equation S[38], one can extract K_{D3} ($\equiv k_{-3}/k_3$) and $[D_0]_{TB}$.

7.1.3 Determination of K_{D2} ($\equiv k_{-2}/k_2$) and $[D_0]_{NB}$ from $[PD]_{NB}/[P]_{FD}$

Based on the same quasi-equilibrium approximation between the FD and NB states, the following relation holds:

$$\frac{[\mathrm{PD}]_{\mathrm{NB}}}{[\mathrm{P}]_{\mathrm{FD}}} = \frac{k_2 [\mathrm{D}]_{\mathrm{NB}}}{k_{-2}} \qquad \text{S[39]}$$

By substituting $[\mathrm{D}]_{\mathrm{NB}}$ with $[\mathrm{D_0}]_{\mathrm{NB}}$-$[\mathrm{PD}]_{\mathrm{NB}}$ in Equation S[39] and rearranging the equation leads to the final formula for K_{D2} (=k_{-2}/k_2) and $[\mathrm{D_0}]_{\mathrm{NB}}$.

$$\frac{[\mathrm{PD}]_{\mathrm{NB}}}{[\mathrm{P}]_{\mathrm{FD}}} = \frac{[\mathrm{D_0}]_{\mathrm{NB}}}{K_{\mathrm{D2}} + [\mathrm{P}]_{\mathrm{FD}}} \qquad \text{S[40]}$$

Equation S[40] shows the relation between $[\mathrm{PD}]_{\mathrm{NB}}/[\mathrm{P}]_{\mathrm{]FD}}$, K_{D2} ($\equiv k_{-2}/k_2$), and $[\mathrm{D_0}]_{\mathrm{NB}}$. By fitting $[\mathrm{PD}]_{\mathrm{NB}}/[\mathrm{P}]_{\mathrm{FD}}$ *vs* $[\mathrm{PD}]_{\mathrm{NB}}$ with Equation S[40], one can extract K_{D2} ($\equiv k_{-2}/k_2$) and $[\mathrm{D_0}]_{\mathrm{NB}}$.

7.1.4 Example of above population analysis

From the experimentally measured cellular protein concentration $[\mathrm{P}]_{\mathrm{cell}}$, we could obtain $[\mathrm{P}]_{\mathrm{FD}}$, $[\mathrm{PD}]_{\mathrm{NB}}$, $[\mathrm{PD}]_{\mathrm{TB}}$ via $[\mathrm{P}]_{\mathrm{cell}}\times A_{\mathrm{FD}}$, $[\mathrm{P}]_{\mathrm{cell}}\times A_{\mathrm{NB}}$, and $[\mathrm{P}]_{\mathrm{cell}}\times A_{\mathrm{TB}}$, respectively, where A_{FD}, A_{NB}, and A_{TB} are the fractional populations of each diffusion state determined from the CDF analysis in Section 4. We could then obtain additional kinetic parameters (e.g., k_1 and k_2) and thermodynamic parameters (e.g., K_{D1} and K_{D2}) from $[\mathrm{PD}]_{\mathrm{TB}}/[\mathrm{P}]_{\mathrm{FD}}$ vs. $[\mathrm{P}]_{\mathrm{FD}}$, $[\mathrm{PD}]_{\mathrm{NB}}/[\mathrm{P}]_{\mathrm{FD}}$ vs. $[\mathrm{P}]_{\mathrm{FD}}$, and $[\mathrm{PD}]_{\mathrm{TB}}/[\mathrm{P}]_{\mathrm{NB}}$ vs. $[\mathrm{PD}]_{\mathrm{NB}}$ (Equation S[34], S[38], and S[40] using values of $k_{\mathrm{o}}^{\mathrm{off}}$, k_{r}, k_{f}, and K_{m} pre-determined from analysis of k_{-1} (e.g., Figure 2D). In the fitting routine, we used a fixed value (i.e., 5) for the oligomerization number (n_{o}), which is the maximum number of proteins at a TB site and should be greater than two (e.g., in the case of dimer of dimer is n_{o}=2). The dependence of the extracted parameters on the value of n_0 will be discussed in Section 7.5.

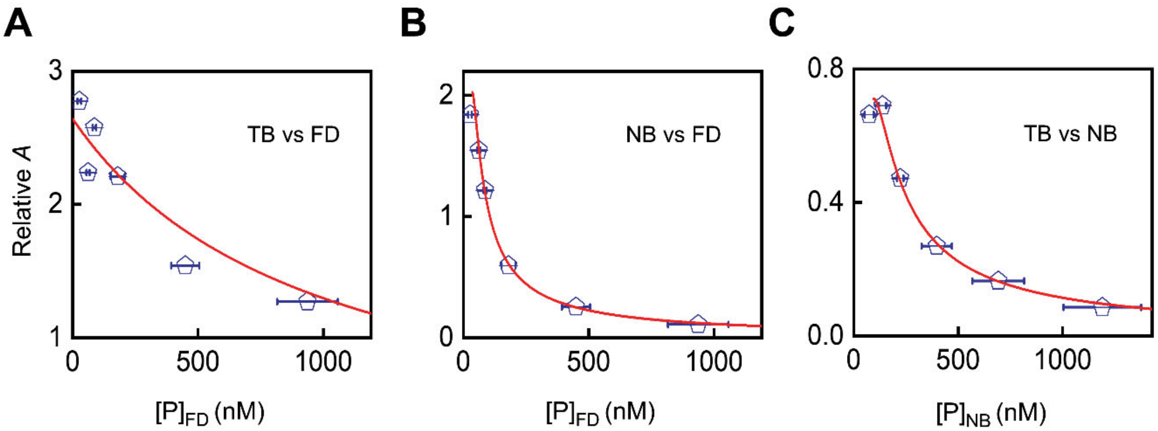

Figure S14. Example of relative population analysis of different diffusion states of $\mathrm{Zur}^{\mathrm{mE}}_{\mathrm{C88S}}$ in cells. The fractional population data of (*A*) $[\mathrm{PD}]_{\mathrm{TB}}/[\mathrm{P}]_{\mathrm{FD}}$ vs. $[\mathrm{P}]_{\mathrm{FD}}$, (*B*) $[\mathrm{PD}]_{\mathrm{NB}}/[\mathrm{P}]_{\mathrm{FD}}$ vs. $[\mathrm{P}]_{\mathrm{FD}}$, and (*C*) $[\mathrm{PD}]_{\mathrm{TB}}/[\mathrm{P}]_{\mathrm{NB}}$ vs. $[\mathrm{PD}]_{\mathrm{NB}}$ are plotted as blue open circles. Red curves represent the fits with corresponding equations. Error bars are s.d.

7.2 Summary of extracted kinetic and thermodynamic parameters

We extracted the kinetic and thermodynamic parameters for all $\mathrm{Zur}^{\mathrm{mE}}$ variants and summarized in Table S7. Error bars of parameters are standard deviation and obtained from 95% confidence bound of curve fitting.

Table S7. Kinetic and thermodynamic parameters for Zur-DNA interaction in *E. coli* cells

Parameters	$\mathrm{Zur}^{\mathrm{mE}}$	$\mathrm{Zur}^{\mathrm{mE}}_{\mathrm{C88S}}$	$\mathrm{Zur}^{\mathrm{mE}\,b}_{\mathrm{Zn}}$	$\mathrm{Zur}^{\mathrm{mE}}_{\mathrm{C88S,\,D49A}}$	$\mathrm{Zur}^{\mathrm{mE}\,b}_{\mathrm{Zn,\,D49A}}$
k_1($\mathrm{nM^{-1}\,s^{-1}}$) [a]	0.80 ± 0.07	0.77 ± 0.08	0.46 ± 0.08	0.68 ± 0.24	0.55 ± 0.08
k_o^{off} ($\mathrm{s^{-1}}$)	25 ± 12	22 ± 21	5.4 ± 0.6	22 ± 2	36 ± 41
k_r ($\mathrm{s^{-1}}$)	16 ± 11	12 ± 20	no. obs.[c]	21 ± 1	27 ± 40
k_f ($\mathrm{nM^{-1}\,s^{-1}}$)	0.012 ± 0.002	0.018 ± 0.003	0.011 ± 0.014	0.021 ± 0.006	0.026 ± 0.004
K_m (nM)	14 ± 10	12 ± 17	no. obs. [c]	39 ± 18	7.6 ± 4.5
$[\mathrm{P}]_{\mathrm{FD}}^{\mathrm{min}}$ (nM)	65 ± 8	47 ± 16	< 8.8	130 ± 10	37 ± 6
K_{d1} ($=k_o^{\mathrm{off}}/k_1$) (nM) [a]	31 ± 15	28 ± 27	12 ± 3	33 ± 12	67 ± 48
K_{d2} ($=k_{-2}/k_2$) (nM) [a]	990 ± 80	830 ± 200	1300 ± 400	500 ± 160	1300 ± 300
K_{d3} ($=k_{-3}/k_3$) [a]	0.011 ± 0.002	0.023 ± 0.007	0.022 ± 0.023	0.032 ± 0.062	0.008 ± 0.006
$[\mathrm{D}_0]_{\mathrm{TB}}$(nM) [a]	20 ± 1	16 ± 2	26 ± 7	15 ± 2	19 ± 2
$[\mathrm{D}_0]_{\mathrm{NB}}$ (nM) [a]	2700 ± 200	2300 ± 500	2900 ± 700	2000 ± 500	3700 ± 800
$[\mathrm{D}_0]_{\mathrm{TB}}\cdot n_o$(nM) [a]	100 ± 2	82 ± 8	130 ± 30	75 ± 12	92 ± 9

[a] $n_o = 5$ was used in fitting.
[b] 20 μM of Zn^{2+} was used for zinc stress condition.
[c] Not observed.

Based on the extracted kinetic and thermodynamic parameters, we observed that:

1) **Non-repressor form of Zur can bind to chromosome tightly (almost as tight as repressor form of Zur).** The binding affinity of $\mathrm{Zur_{C88S}}$ ($K_{d1} = 27.9 \pm 26.7$ nM) to TB sites is comparable to that of repressor form of Zur ($K_{d1} = 11.7 \pm 2.9$ nM), and much stronger than that to NB sites ($K_{d2} = 829 \pm 200$ nM). The strong binding affinity of non-repressor form of Zur likely comes from its binding to non-consensus sites because previous study showed that non-repressor form of Zur does not bind to consensus operator sites(1).

2) **The chromosome has similar binding capability for non-repressor and repressor form of Zur, with a few tens of tight-binding sites.** The value of $[\mathrm{D}_0]_{\mathrm{TB}}\cdot n_o$ for all Zur variants ranges from 80 to 120 nM. Assuming $n_0 \geq 2$ for the oligomerization number, the concentration of Zur binding sites in the cell would be <60 nM, corresponding to <60 sites.

3) **Salt-bridge mutation (D49A) weakens the oligomerization tendency.** The K_m of repressor form of Zur (i.e., $\mathrm{Zur}^{\mathrm{mE}}_{\mathrm{Zn}}$) was not measurable since it is lower than our experimentally accessible cellular protein concentration (~8.8 nM), whereas that of $\mathrm{Zur}^{\mathrm{mE}}_{\mathrm{Zn,\,D49A}}$ became measurable and its value is 7.6 ± 4.5 nM. The K_m of non-repressor form of Zur (i.e., $\mathrm{Zur}^{\mathrm{mE}}_{\mathrm{C88S}}$) is 11.7 ± 17.4 nM whereas that of $\mathrm{Zur}^{\mathrm{mE}}_{\mathrm{C88S,\,D49A}}$ is 38.6 ± 17.9 nM, reflecting a 2~3 times weakened oligomerization tendency.

4) **Binding rate constants were observed to be similar among all Zur variants while the unbinding rate constants varied significantly.** Therefore, regulation of unbinding kinetics, rather than the binding kinetics, is a more critical factor in determining the binding affinity. For example, the binding rate constant k_1 to a TB site for $\mathrm{Zur}^{\mathrm{mE}}_{\mathrm{C88S}}$ is 0.77 ± 0.08 $\mathrm{nM^{-1}\,s^{-1}}$, slightly larger than that of $\mathrm{Zur}^{\mathrm{mE}}_{\mathrm{Zn}}$ (0.46 ± 0.08 $\mathrm{nM^{-1}\,s^{-1}}$), indicating that non-repressor form of Zur's weaker binding to chromosome than repressor form of Zur is more dictated by their unbinding instead of binding rate constants.

5) **Weakened oligomerization (i.e., larger K_m) leads to faster facilitated unbinding (i.e., larger k_f).** The k_f for salt-bridge mutants of repressor and non-repressor (0.026 ± 0.004 $\mathrm{s^{-1}}$, and 0.021 ± 0.006 $\mathrm{s^{-1}}$, respectively) are greater than that for non-mutated forms of repressor and non-repressor (0.011 ± 0.014

s^{-1}, and 0.018 ± 0.003 s^{-1}, respectively). This is perhaps due to easier formation of the ternary complex where two Zur dimers each bind partly to DNA (Figure 2E, lower).

6) The parameters extracted for Zur^{mE} in the absence of added zinc are between those of Zur^{mE}_{C88S} and Zur^{mE}_{Zn}, consistent with **Zur^{mE}'s behavior being a mixture of repressor and non-repressor forms of Zur.**

7) **Rebinding by repressors to a vacant TB site occurs within ~0.11 s** obtained from the inverse of binding rate (which equal to k_1 multiplied by the average physiological free-diffusing concentration $[Zur^{mE}_{Zn}]_{FD}$; Figure 4A).

7.3 Biphasic unbinding is not due to different sampling of various TB sites with increasing cellular protein concentrations

It should be noted that the measured unbinding/binding kinetic and thermodynamic parameters in Table S7 are properties averaged over all possible binding sites of Zur in the chromosome, since our single-molecule tracking cannot selectively measure one particular site in the cell. Past in vitro work has shown that *E. coli* Zur, in its repressor form, has different binding affinities to different promoter sites(1). For example, its K_d for simultaneous/cooperative two dimer binding to *zinT* and *znuC* promoter recognition sites are 0.053 ± 0.01 and 8.2 ± 0.7 (× 10^{-18} M^2), respectively(1). These two-dimer-binding K_d's would correspond to apparent one-dimer binding affinities of ~0.23 nM and ~2.9 nM, respectively.

This variable binding affinity of Zur to different binding sites could potentially lead to different sampling among these sites when the free Zur protein concentration in the cell varies in the range of comparable to or lower than Zur binding affinities — at concentrations a few times higher than the binding affinities, all binding sites would be fully occupied — which could be perceived as a possible cause for the biphasic unbinding behaviors we observed in the cell. Here we list the evidences and rationales against this possibility.

1) The reported effective binding affinity of Zur repressor one dimer binding to *zinT* and *znuC* is sub to a few nM(1). Our measured K_{d1}, the binding affinity to the tight-binding sites, for various forms of Zur in the cell are ~10 nM to ~30 nM (Table S7). All these affinities are lower than or comparable to the lower limit of our experimental accessible cellular Zur concentration (~70 to 95 nM in total Zur concentration, and ~8.8 to 15.5 nM in free Zur concentration; e.g., Figure 2D in the main text). Therefore, under our experimental conditions, all TB sites are mostly occupied and thus their relative samplings are mostly unchanged throughout the cellular Zur concentration range we studied.

2) If all sites *were only* to possess facilitated unbinding, it will never give rise to impeded unbinding behaviors even if different sites are sampled differently with increasing Zur concentrations in the cell.

3) The facilitated unbinding behaviors occur toward *higher* concentrations of Zur in the cell, under which all binding sites would be at even higher probability of being dominantly occupied, giving invariant sampling of all sites in the cell in our measurements.

4) The impeded unbinding behaviors occur at lower cellular concentration range of Zur. This impeded unbinding, if it were to have resulted from different sampling of binding sites, it would mean that with increasing protein concentration, the measurement samples more of those sites that have slower unbinding rates. This is counter intuitive (or maybe unreasonable), because, at higher protein concentrations, the weaker binding sites would get more populated and sampled, but these weaker ones would typically have faster unbinding kinetics instead of slower, assuming weaker affinity is associated with faster unbinding.

7.4 Validation of quasi-static system approximation for using a linear-combination of three-diffusion states

With extracted kinetic parameters, we can validate quasi-static system approximation in which we introduced in Section 4.2 for using a linear-combination of CDFs. Recall, to linearly combine three diffusion states of CDF, time-resolution in experiment should be comparable or faster than the interconversion rates between states. Using $\mathrm{Zur}_{\mathrm{Zn}}^{\mathrm{mE}}$ as an example, at the highest cellular protein concentration $[\mathrm{Zur}_{\mathrm{Zn}}^{\mathrm{mE}}]_{\mathrm{cell}}$ of ~2200 nM (corresponding to $[\mathrm{Zur}_{\mathrm{Zn}}^{\mathrm{mE}}]_{\mathrm{FD}}$ ~970 nM) where all binding/unbinding steps are fastest, we examined the apparent interconversion rates between the states and their corresponding time scales. The computed unbinding rates of TB (k_{-1}) and NB (k_{-2}) are 17.2 and 12.1 s^{-1} (corresponding timescales are ~58 and 83 ms, respectively). The apparent binding rates of TB ($k_1[\mathrm{D}]_{\mathrm{TB}}$) and NB ($k_2[\mathrm{D}]_{\mathrm{NB}}$) are ~1.39 and 12.33 s^{-1} (corresponding timescales are 722 and 81 ms, respectively). Imaging time lapse of 40 ms is faster than these timescales of interconversion between states, which can validate the quasi-static system approximation.

We evaluated the contribution of data artifacts into parameters quantification potentially arising from imaging time-lapse length in our previous study (4). There, we performed three-state diffusion simulation inside a cell-boundary with known input parameters. Using the same data analysis procedure on the simulated results showed that even at the fastest inter-converting conditions (interconversion rate constants ~16.8 s^{-1}, faster/comparable to what's observed for Zur), the extracted rates was ~14.4 ± 1.0 s^{-1}, in agreement with the input value. With a range of inputs in simulation, the extracted rate differed by ~15%, but holds the same trends when protein concentration is varied. Therefore, we expected that the contribution of data artifacts is < 15% and our data analysis procedure with the quasi-static system approximation is valid and reliably reproduces the trend of its dependence on the cellular protein concentration.

7.5 n_o dependence of extracted kinetic and thermodynamic parameters

Previous *in vitro*, and *in silico* studies identified four *zur*-regulons including zinc uptake gene cluster (*znuABC*), ribosomal protein (*L31p* and *L36p*), periplasmic zinc trafficking protein (*zinT*), and lysozyme inhibitor (*pliG*) (1,5,40,41). These 4 known consensus sites essentially set the lower limit for $[\mathrm{D}_0]_{\mathrm{TB}}$ for the repressor form of Zur (i.e., ~4 nM; note 1 copy corresponds to ~1 nM in a *E. coli* cell). We obtained 80 - 120 nM for $[\mathrm{D}_0]_{\mathrm{TB}}\cdot n_o$ for all Zur variants indicating that n_o is <20. In our fitting routine, we had to choose a value of n_0 in advance, required by our numerical fitting algorithm, and we chose to use a fixed oligomerization number $n_o = 5$. Here we evaluate the effect of the value of n_0 on the fitting results. Two parameters, k_1 and $[\mathrm{D}_0]_{\mathrm{TB}}\cdot n_o$ were chosen to show the n_0 dependence (note, k_1 is the parameter in the summation; $F_{\mathrm{TB}\leftarrow\mathrm{FD}}\left([\mathrm{P}]_{\mathrm{FD}}\right)=\sum_{i=0}^{n_0}\left(\frac{k_1}{k_{\mathrm{d}}}[\mathrm{P}]_{\mathrm{FD}}\right)^i$ in Equation S[34], meaning that this parameter is directly influenced by n_o in fitting). If these two asymptotically approach certain values, other thermodynamic quantities such as K_{d2} and $[\mathrm{D}_0]_{\mathrm{NB}}$ should approach their asymptotic values since parameters in fitting are all shared (see Equation S[34], Equation S[38] and Equation S[40]). Figure S15 shows that in the range where n_o >3, the extracted k_1 and $[\mathrm{D}_0]_{\mathrm{TB}}\cdot n_o$ approach to asymptotic values within their error bars. Therefore, the oligomerization number n_o does not significantly influence the extracted parameters.

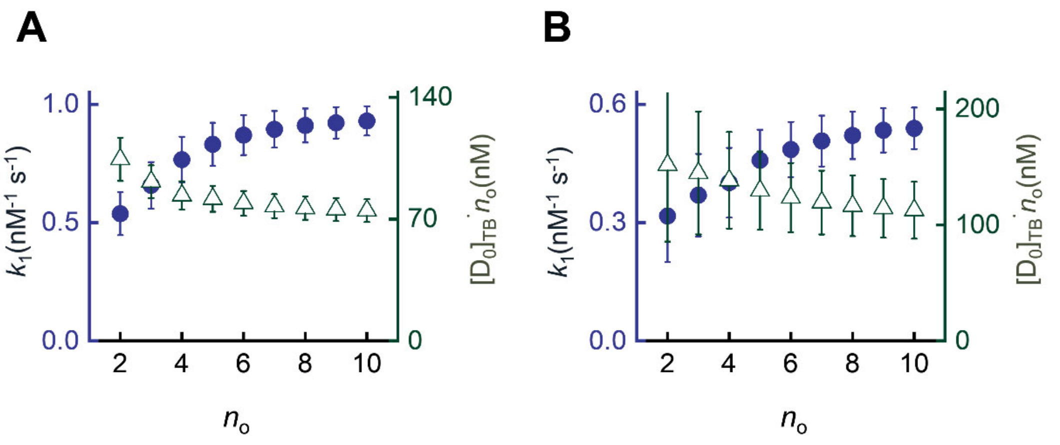

Figure S15. n_o dependence of the extracted k_1 (blue circles) and $[D_0]_{TB}\cdot n_o$ (green triangles) for (A) Zur^{mE}_{C88S} and (B) Zur^{mE}_{Zn}. Error bars are s.d.

8. Clustering analysis of residence sites

8.1 Pairwise distance distributions (PWDD) analysis of residence sites and the simulated random sites

Residence sites are the locations associated with the residence times of Zur^{mE} obtained by r_o-thresholding the displacement vs. time trajectories (Section 5), which are dominated by Zur binding to the tight binding sites on the chromosome. An example of residence sites in a cell are plotted in Figure S16A with the corresponding cell outline, which was determined from the cell's optical transmission image.

Zur^{mE} clustering was first examined by computing the pairwise distance distribution (PWDD) of the residence sites. The pairwise distance is the Euclidean distance between positions (i.e., sites). Only the 1st position within each residence time was sampled in computing the PWDD to avoid oversampling toward relatively longer residence times. Residence sites in each group of cells having a similar protein concentration were compiled to minimize skewing the PWDD toward cells of higher cellular protein concentrations that have higher densities of sampled points. Within each group of cells of a similar protein concentration, those cells having more clustered residence sites are expected to have shorter pair-wise distances.

As a control, identical numbers of randomized locations were simulated using a home-written MATLAB program within each model cell geometry (i.e., a cylinder with 2 hemisphere caps) whose length and width were taken as the averages of the cells in each group, measured from the optical transmission images (Section 3). Sampling was repeated thirty times and averaged for statistical saturation. The simulation was also done in 3-dimension, and a subsequent 2-D projection was used to directly compare with the 2-D experimental data (Figure S16B).

The PWDDs of residence sites for both the Zur^{mE}_{C88S} and Zur^{mE}_{Zn} show a peak at a shorter distance than that from the simulation of randomly distributed locations, supporting the clustering of Zur proteins in the cell, presumably due to oligomerization at tight binding sites on the chromosome (Figure 3A and Figure S17A).

We used data grouping to determine the confidence level of the experimental PWDDs. To do so, we divided the experimental pair-wise distances randomly into 10 groups, each containing equal number of pair-wise distances. We then computed the PWDD for each of the 10 groups, and computed the average

and standard deviation of these 10 PWDDs. From the standard deviation, we computed the 95% confidence bounds, which are shown as the gray area in the lower panels of Figure 3A and Figure S17A.

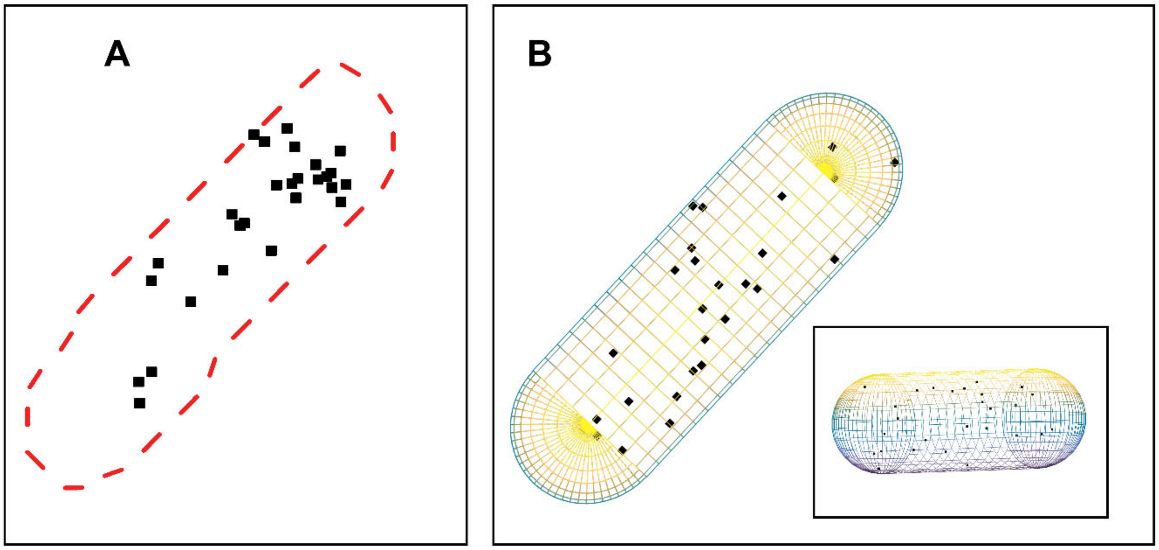

Figure S16. (*A*) An example of residence sites (black squares) of Zur^{mE}_{C88S} in a cell. The cell outline is indicated by the dashed red line. (*B*) *xy*-projected view of randomly sampled 3-D points within a model cell of the same length and width. Inset: Same positions with a *yz*-projected view.

8.2 Analysis of the fraction of residence sites within a threshold distance *R* to decouple the effect of chromosome condensation

Our previous research on the metalloregulator CueR and ZntR used the averaged pairwise distance between residence sites, $\langle d_{ij} \rangle$ as a measure for the extent of chromosome compactness, where cells with more compacted chromosome have shorter $\langle d_{ij} \rangle$(4); that analysis was based on that CueR/ZntR has a large number of recognition sites scattered across the chromosome randomly and that CueR/ZntR do not show any oligomerization behaviors. The quantified number of $\langle d_{ij} \rangle$ was ~0.68 μm for compacted chromosome, and 0.88 μm for less compacted chromosome. Since the extent of chromosome compactness differs from cell to cell, the $\langle d_{ij} \rangle$ are broadly distributed among cells implying that chromosome compactness (i.e., condensation) could contribute to shorter distances in the pair-wise distance distributions of residence sites.

To decouple the contribution of protein oligomerization from chromosome condensation in the PWDD analysis, we computed the fraction of residence sites within a radius threshold distance *R* and compared with that of simulated random sites. Figure 3B and Figure S17B present the fraction of residence sites with different values of *R* as a function of cellular protein concentration. For both the experimental and simulated residence sites, the fraction of residence sites within *R* increases with increasing protein concentration, because higher concentrations gave higher densities of sampled residence sites in a cell. When *R* became shorter from 0.2 μm down to 0.07 μm, the increase was slower (Figure S17).

On the other hand, the difference of the fraction within *R* between the experimental data and simulated random sites becomes larger as *R* is smaller (Figure S17B, red vs. blue). We calculated the averaged ratio within *R* of the fraction of observed residence sites over that of the simulated random sites; this average ratio shows a clear increase with decreasing *R* (Figure S17C). This trend supports the

occurrence of oligomerization of Zur^{mE} at TB sites because protein oligomerization is a molecular scale process (i.e., within short distances) whereas chromosome compactness is much larger in dimension (i.e., around hundreds of nanometer), and at decreasing threshold distance *R*, protein oligomerization would dominate over chromosome condensation in producing clustering of residence sites.

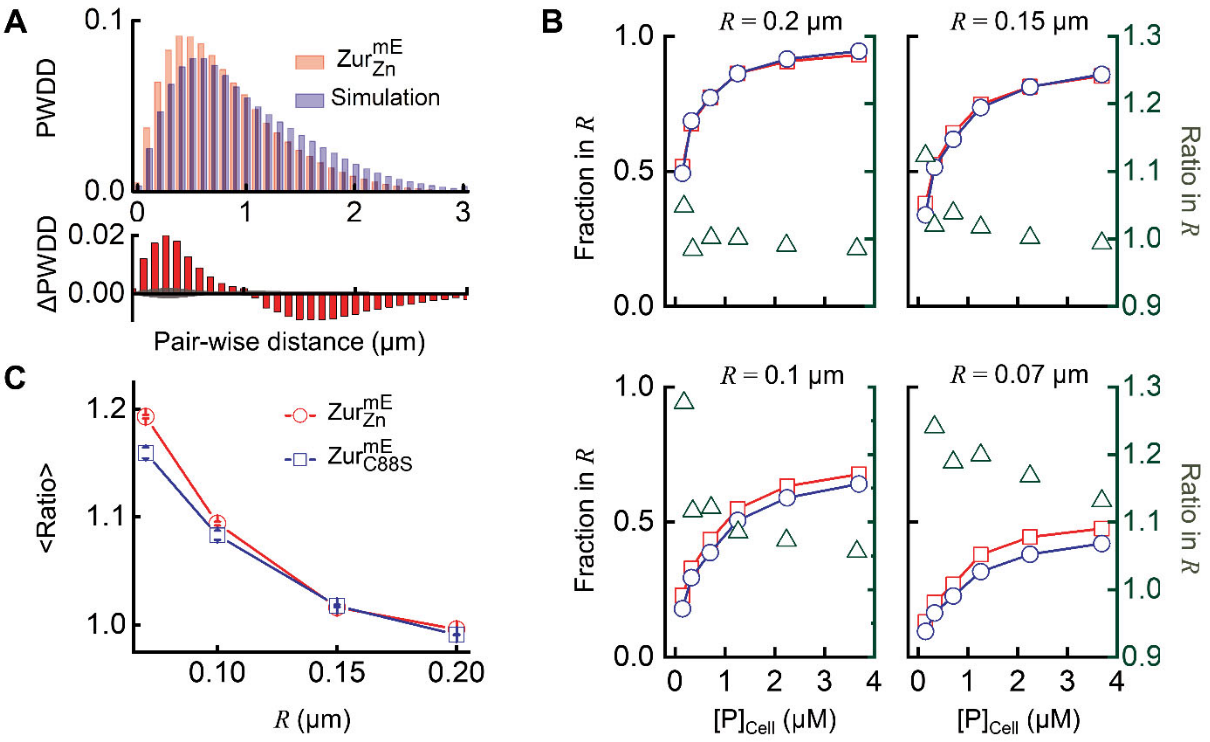

Figure S17. Clustering analysis of Zur's residence sites in cells. (A) Normalized PWDD of residence sites for Zur$^{mE}_{Zn}$, and of simulated random localizations (top) at [Zur$^{mE}_{Zn}$] = 705 ± 140 nM, and the difference of the normalized PWDD of Zur$^{mE}_{Zn}$ from that of simulation (bottom; the gray area indicates 95% confidence). (B) The fraction of residence sites within the threshold distance *R* (left axis) for Zur$^{mE}_{Zn}$ (red curve) and simulated random locations (blue curve) vs cellular protein concentration, as well as their ratio (green triangle, right axis). Each panel represents a different *R* value. (C) Dependence of the averaged ratio in (*B*) on *R* for both Zur$^{mE}_{Zn}$ and Zur$^{mE}_{C88S}$.

9. Bootstrap of CDF analysis shows statistical reliability

We performed bootstrap analysis to show that our extracted results from analyzing the CDF of displacement length *r* are statistically reliable. We first randomly sampled 25%, 50%, and 75% from all displacement lengths and performed the CDF analysis as described in Section 4.1. Then we obtained the ratio of extracted diffusion constants (*D*'s) and its fractional populations (*A*'s) from randomly sampled data over those final results in Table S5 and Table S6. Figure S18 clearly shows that the results extracted from randomly sampled *r* are within 2% of error from the final results. Note that error bars in CDF fitting of extracted diffusion constants and the corresponding fraction populations in Table S5 and Table S6 correspond to ~7% and ~3% of its values, respectively. These results indicate that even using just 25% of the experimental data, we can determine diffusion constants of these states and their fractional populations within 2% error, supporting that our data are statistically saturated and reliable.

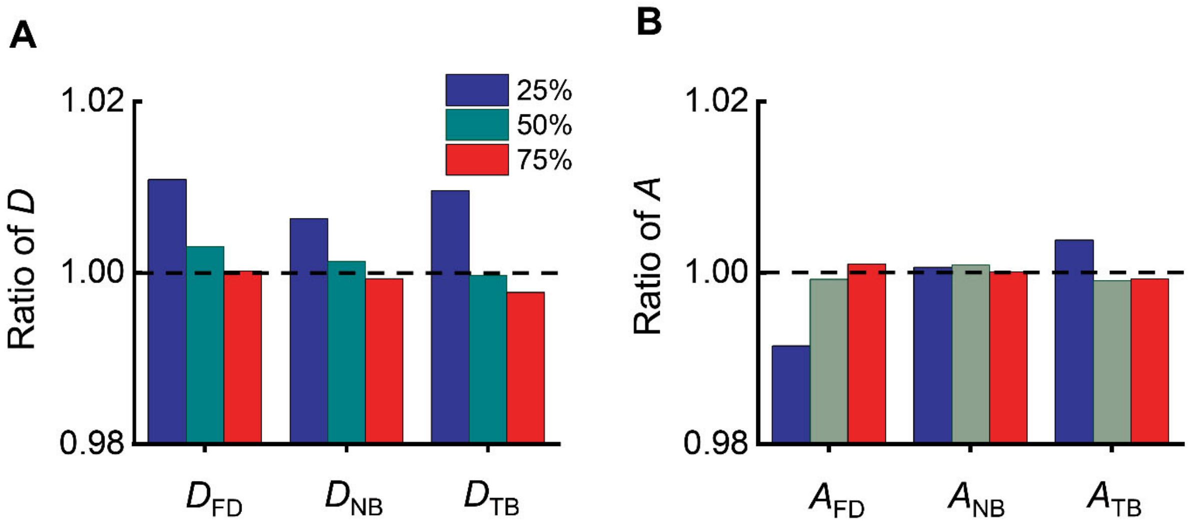

Figure S18. Statistical saturation in CDF analysis. (***A***) Ratio of extracted diffusion constants from randomly sampled 25% (blue), 50% (green), and 75% (red) of displacement lengths (*r*) over those from using overall 100% displacement lengths for Zur^{mE}, and (***B***) its corresponding fractional populations at $[Zur^{mE}] = 407 \pm 33$ nM.

10. List of abbreviations and strain names

Table S8. Abbreviations used in this study

Abbreviation	Complete term
SMT	Single-molecule tracking
SCQPC	Single cell quantification of protein concentration
WT	Wild-type
Zur	Zinc uptake regulator
TPEN	N,N,N′,N′-tetrakis(2-pyridinylmethyl)-1,2-ethanediamine
PDF	Probability distribution function
CDF	Cumulative distribution function
FD	Freely diffusing
NB	Non-specifically bound
TB	Tightly bound
PWDD	Pair-wise distance distribution
RT-PCR	Reverse-transcription polymerase chain reaction
iqPALM	Image-based quantitative photo-activated localization microscopy

Table S9. Strain names in this study

Strain name	Description
Zur^{mE}	DZ-pZurmE strain
Δ*zur*	JW5714-1 strain
Zur^{mE}_{C88S}	DZ-pApoZurmE strain
Zur^{mE}_{D49A}	DZ-pZurD49AmE strain
$Zur^{mE}_{C88S,\ D49A}$	DZ-pApoZurD49AmE strain
Zur^{mE}_{Zn}	Zur^{mE} under 20 μM Zn^{2+} replete conditions
$Zur^{mE,Chr}_{Zn+pZur\text{-}C88S}$	Zur^{mE}_{Zn} with a plasmid expressing Zur_{C88S} and mE-tagged Zur is only encoded on the chromosome

Additional References

1. Gilston, B.A., Wang, S., Marcus, M.D., Canalizo-Hernandez, M.A., Swindell, E.P., Xue, Y., Mondragon, A. and O'Halloran, T.V. (2014) Structural and mechanistic basis of zinc regulation across the E. coli Zur regulon. *PLoS Biol.*, **12**, e1001987.
2. Datsenko, K.A. and Wanner, B.L. (2000) One-step inactivation of chromosomal genes in Escherichia coli K-12 using PCR products. *Proc. Natl. Acad. Sci. U. S. A.*, **97**, 6640-6645.
3. Guzman, L.M., Belin, D., Carson, M.J. and Beckwith, J. (1995) Tight regulation, modulation, and high-level expression by vectors containing the arabinose PBAD promoter. *J. Bacteriol.*, **177**, 4121-4130.
4. Chen, T.Y., Santiago, A.G., Jung, W., Krzeminski, L., Yang, F., Martell, D.J., Helmann, J.D. and Chen, P. (2015) Concentration- and chromosome-organization-dependent regulator unbinding from DNA for transcription regulation in living cells. *Nat. Commun.*, **6**, 7445.
5. Panina, E.M., Mironov, A.A. and Gelfand, M.S. (2003) Comparative genomics of bacterial zinc regulons: enhanced ion transport, pathogenesis, and rearrangement of ribosomal proteins. *Proc. Natl. Acad. Sci. U. S. A.*, **100**, 9912-9917.
6. Gao, W., Zhang, W. and Meldrum, D.R. (2011) RT-qPCR based quantitative analysis of gene expression in single bacterial cells. *J. Microbiol. Methods*, **85**, 221-227.
7. Schmittgen, T.D. and Livak, K.J. (2008) Analyzing real-time PCR data by the comparative C(T) method. *Nature protocols*, **3**, 1101-1108.
8. Cho, Y.E., Lomeda, R.A., Ryu, S.H., Lee, J.H., Beattie, J.H. and Kwun, I.S. (2007) Cellular Zn depletion by metal ion chelators (TPEN, DTPA and chelex resin) and its application to osteoblastic MC3T3-E1 cells. *Nutr Res Pract*, **1**, 29-35.
9. Shin, J.H., Oh, S.Y., Kim, S.J. and Roe, J.H. (2007) The zinc-responsive regulator Zur controls a zinc uptake system and some ribosomal proteins in Streptomyces coelicolor A3(2). *J. Bacteriol.*, **189**, 4070-4077.
10. Milano, A., Branzoni, M., Canneva, F., Profumo, A. and Riccardi, G. (2004) The Mycobacterium tuberculosis Rv2358-furB operon is induced by zinc. *Res. Microbiol.*, **155**, 192-200.
11. Canneva, F., Branzoni, M., Riccardi, G., Provvedi, R. and Milano, A. (2005) Rv2358 and FurB: two transcriptional regulators from Mycobacterium tuberculosis which respond to zinc. *J. Bacteriol.*, **187**, 5837-5840.
12. Latorre, M., Low, M., Garate, E., Reyes-Jara, A., Murray, B.E., Cambiazo, V. and Gonzalez, M. (2015) Interplay between copper and zinc homeostasis through the transcriptional regulator Zur in Enterococcus faecalis. *Metallomics*, **7**, 1137-1145.
13. Ellison, M.L., Farrow, J.M., 3rd, Parrish, W., Danell, A.S. and Pesci, E.C. (2013) The transcriptional regulator Np20 is the zinc uptake regulator in Pseudomonas aeruginosa. *PLoS One*, **8**, e75389.
14. Prestel, E., Noirot, P. and Auger, S. (2015) Genome-wide identification of Bacillus subtilis Zur-binding sites associated with a Zur box expands its known regulatory network. *BMC Microbiol.*, **15**, 13.
15. Elf, J., Li, G.W. and Xie, X.S. (2007) Probing transcription factor dynamics at the single-molecule level in a living cell. *Science*, **316**, 1191-1194.
16. English, B.P., Hauryliuk, V., Sanamrad, A., Tankov, S., Dekker, N.H. and Elf, J. (2011) Single-molecule investigations of the stringent response machinery in living bacterial cells. *Proc. Natl. Acad. Sci. U. S. A.*, **108**, E365-373.
17. Mehta, P., Jovanovic, G., Lenn, T., Bruckbauer, A., Engl, C., Ying, L. and Buck, M. (2013) Dynamics and stoichiometry of a regulated enhancer-binding protein in live Escherichia coli cells. *Nat. Commun.*, **4**, 1997.
18. Bakshi, S., Bratton, B.P. and Weisshaar, J.C. (2011) Subdiffraction-limit study of Kaede diffusion and spatial distribution in live Escherichia coli. *Biophys. J.*, **101**, 2535-2544.

19. Mazza, D., Abernathy, A., Golob, N., Morisaki, T. and McNally, J.G. (2012) A benchmark for chromatin binding measurements in live cells. *Nucleic Acids Res.*, **40**, e119.
20. Javer, A., Long, Z., Nugent, E., Grisi, M., Siriwatwetchakul, K., Dorfman, K.D., Cicuta, P. and Cosentino Lagomarsino, M. (2013) Short-time movement of E. coli chromosomal loci depends on coordinate and subcellular localization. *Nat. Commun.*, **4**, 3003.
21. Durisic, N., Laparra-Cuervo, L., Sandoval-Alvarez, A., Borbely, J.S. and Lakadamyali, M. (2014) Single-molecule evaluation of fluorescent protein photoactivation efficiency using an in vivo nanotemplate. *Nat. Methods*, **11**, 156-162.
22. Annibale, P., Scarselli, M., Greco, M. and Radenovic, A. (2012) Identification of the factors affecting co-localization precision for quantitative multicolor localization microscopy. *Optical Nanoscopy*, **1**.
23. Thompson, R.E., Larson, D.R. and Webb, W.W. (2002) Precise Nanometer Localization Analysis for Individual Fluorescent Probes. *Biophys. J.*, **82**, 2775-2783.
24. Yildiz, A., Forkey, J.N., McKinney, S.A., Ha, T., Goldman, Y.E. and Selvin, P.R. (2003) Myosin V walks hand-over-hand: single fluorophore imaging with 1.5-nm localization. *Science*, **300**, 2061-2065.
25. Gebhardt, J.C., Suter, D.M., Roy, R., Zhao, Z.W., Chapman, A.R., Basu, S., Maniatis, T. and Xie, X.S. (2013) Single-molecule imaging of transcription factor binding to DNA in live mammalian cells. *Nat. Methods*, **10**, 421-426.
26. Cunha, S., Woldringh, C.L. and Odijk, T. (2005) Restricted diffusion of DNA segments within the isolated Escherichia coli nucleoid. *Journal of structural biology*, **150**, 226-232.
27. Mazza, D., Ganguly, S. and McNally, J.G. (2013) Monitoring dynamic binding of chromatin proteins in vivo by single-molecule tracking. *Methods Mol. Biol.*, **1042**, 117-137.
28. Golding, I. and Cox, E.C. (2006) Physical nature of bacterial cytoplasm. *Phys. Rev. Lett.*, **96**, 098102.
29. Chen, T.Y., Jung, W., Santiago, A.G., Yang, F., Krzeminski, L. and Chen, P. (2015) Quantifying Multistate Cytoplasmic Molecular Diffusion in Bacterial Cells via Inverse Transform of Confined Displacement Distribution. *J. Phys. Chem. B*, **119**, 14451-14459.
30. Yang, F., Chen, T.Y., Krzeminski, L., Santiago, A.G., Jung, W. and Chen, P. (2016) Single-molecule dynamics of the molecular chaperone trigger factor in living cells. *Mol. Microbiol.*, **102**, 992-1003.
31. Joshi, C.P., Panda, D., Martell, D.J., Andoy, N.M., Chen, T.Y., Gaballa, A., Helmann, J.D. and Chen, P. (2012) Direct substitution and assisted dissociation pathways for turning off transcription by a MerR-family metalloregulator. *Proc. Natl. Acad. Sci. U. S. A.*, **109**, 15121-15126.
32. Graham, J.S., Johnson, R.C. and Marko, J.F. (2011) Concentration-dependent exchange accelerates turnover of proteins bound to double-stranded DNA. *Nucleic Acids Res.*, **39**, 2249-2259.
33. Gibb, B., Ye, L.F., Gergoudis, S.C., Kwon, Y., Niu, H., Sung, P. and Greene, E.C. (2014) Concentration-dependent exchange of replication protein A on single-stranded DNA revealed by single-molecule imaging. *PLoS One*, **9**, e87922.
34. Geertsema, H.J., Kulczyk, A.W., Richardson, C.C. and van Oijen, A.M. (2014) Single-molecule studies of polymerase dynamics and stoichiometry at the bacteriophage T7 replication machinery. *Proc Natl Acad Sci U S A*, **111**, 4073-4078.
35. Lewis, J.S., Spenkelink, L.M., Jergic, S., Wood, E.A., Monachino, E., Horan, N.P., Duderstadt, K.E., Cox, M.M., Robinson, A., Dixon, N.E. *et al.* (2017) Single-molecule visualization of fast polymerase turnover in the bacterial replisome. *Elife*, **6**.
36. Chen, T.Y., Cheng, Y.S., Huang, P.S. and Chen, P. (2018) Facilitated Unbinding via Multivalency-Enabled Ternary Complexes: New Paradigm for Protein-DNA Interactions. *Acc. Chem. Res.*, **51**, 860-868.
37. Andoy, N.M., Sarkar, S.K., Wang, Q., Panda, D., Benitez, J.J., Kalininskiy, A. and Chen, P. (2009) Single-molecule study of metalloregulator CueR-DNA interactions using engineered Holliday junctions. *Biophys. J.*, **97**, 844-852.

38. Choi, S.H., Lee, K.L., Shin, J.H., Cho, Y.B., Cha, S.S. and Roe, J.H. (2017) Zinc-dependent regulation of zinc import and export genes by Zur. *Nat. Commun.*, **8**, 15812.
39. Brunauer, S., Emmett, P.H. and Teller, E. (1938) Adsorption of Gases in Multimolecular Layers. *J. Am. Chem. Soc.*, **60**, 309-319.
40. Hantke, K. (2005) Bacterial zinc uptake and regulators. *Curr. Opin. Microbiol.*, **8**, 196-202.
41. Hemm, M.R., Paul, B.J., Miranda-Rios, J., Zhang, A., Soltanzad, N. and Storz, G. (2010) Small stress response proteins in Escherichia coli: proteins missed by classical proteomic studies. *J. Bacteriol.*, **192**, 46-58.
